# Recurring Cholinergic Inputs Induce Local Hippocampal Plasticity through Feedforward Disinhibition

**DOI:** 10.1523/ENEURO.0389-21.2022

**Published:** 2022-09-01

**Authors:** Inês Guerreiro, Zhenglin Gu, Jerrel L. Yakel, Boris S. Gutkin

**Affiliations:** 1 Group for Neural Theory, LNC2 INSERM U960, Département d'études cognitives, Ecole Normale Superieure, PSL Université Paris, 75005 Paris, France; 2 Neurobiology Laboratory, National Institute of Environmental Health Sciences, National Institutes of Health, Department of Health and Human Services, Research Triangle Park, North Carolina 27709; 3 Center for Cognition and Decision Making, Institute for Cognitive Neuroscience, National Research University Higher School of Economics, Moscow 101000, Russia

**Keywords:** hippocampus, synaptic plasticity, disinhibition, Schaffer Collateral, nicotinic receptors, GABA neurons

## Abstract

The CA1 pyramidal neurons are embedded in an intricate local circuitry that contains a variety of interneurons. The roles these interneurons play in the regulation of the excitatory synaptic plasticity remains largely understudied. Recent experiments showed that recurring cholinergic activation of α7 nACh receptors expressed in oriens-lacunosum-moleculare (OLMα2) interneurons can directly induce LTP in Schaffer collateral (SC)–CA1 synapses. Here, we pair *in vitro* studies with biophysically based modeling to uncover the underlying mechanisms. According to our model, α7 nAChR activation increases OLM GABAergic activity. This results in the inhibition of the fast-spiking interneurons that provide feedforward inhibition onto CA1 pyramidal neurons. This disinhibition, paired with tightly timed SC stimulation, can induce potentiation at the excitatory synapses of CA1 pyramidal neurons. Our work details the role of cholinergic modulation in disinhibition-induced hippocampal plasticity. It relates the timing of cholinergic pairing found experimentally in previous studies with the timing between disinhibition and hippocampal stimulation necessary to induce potentiation and suggests the dynamics of the involved interneurons plays a crucial role in determining this timing.

## Significance Statement

We use a combination of experiments and mechanistic modeling to uncover the key role for cholinergic neuromodulation of feedforward disinhibitory circuits in regulating hippocampal plasticity. We found that cholinergic activation of α7 nAChR expressed in oriens-lacunosum-moleculare interneurons, when tightly paired with stimulation of the Schaffer collaterals, can cancel feedforward inhibition onto CA1 pyramidal cells, enabling the potentiation of the SC–CA1 synapse. Our work details how cholinergic action on GABAergic interneurons can tightly regulate the excitability and plasticity of the hippocampal network, unraveling the intricate interplay of the hierarchal inhibitory circuitry and cholinergic neuromodulation as a mechanism for hippocampal plasticity.

## Introduction

The hippocampal networks are characterized by a variety of locally connected GABAergic interneurons exerting robust control on network excitability. Previous work has detailed the importance of inhibitory inputs in modulating local hippocampal synaptic plasticity (Wigström and Gustafsson, 1985; Meredith et al., 2003; Chevalerye and Piskorowski, 2014; Saudargienė and Graham, 2015). Furthermore, several experimental studies show that disinhibition facilitates the induction of long-term potentiation (LTP) at excitatory synapses (Ormond and Woodin, 2009; Yang et al., 2016). However, how the disinhibition controlling hippocampal excitatory synapses is modulated (e.g., by neuromodulators) is not clearly understood, and the precise circuitry and its dynamics underlying this type of plasticity remains an open question.

GABAergic interneurons receive significant cholinergic innervation from the medial septum. They are endowed with various subtypes of nicotinic ACh receptors (nAChRs) that regulate excitability, plasticity, and cognitive functions (Pitler and Alger, 1992; Behrends and Ten Bruggencate, 1993; Hasselmo et al., 1995; Alkondon et al., 1997; Patil et al., 1998; McQuiston and Madison, 1999; Patil and Hasselmo, 1999; Levin, 2002; Hasselmo, 2006; Parikh et al., 2007; Bell et al., 2011, 2015; Griguoli and Cherubini, 2012; Yakel, 2012; Desikan et al., 2018; Nicholson and Kullmann, 2021). Moreover, alterations of cholinergic action on hippocampal GABAergic interneurons have been implicated in cognitive dysfunction in Alzheimer’s disease (AD; Schmid, et al., 2016). These studies, among others, furnish clear evidence that cholinergic inputs exert a powerful role in regulating hippocampal activity. Still, because of the abundance of cholinergic receptors (both muscarinic and nicotinic) and the complexity of the networks in which they are embedded, it is difficult to access the exact mechanisms through which cholinergic action on the hippocampus modulates its microcircuits.

Previous studies showed that activation of α7 nACh receptors expressed in oriens-lacunosum-moleculare (OLMα2) interneurons increases Schaffer collateral (SC)–CA1 transmission and suggest that this happens through disinhibition by reducing the activity of stratum radiatum (s.r.) interneurons that in turn provide feedforward inhibition onto pyramidal (PYR) neurons (Leão et al., 2012). Consistent with these studies, Gu et al. (2020) found that repeated coactivation of α7 nAChR on OLMα2 interneurons and a local SC pathway increased CA1 EPSCs and reduced IPSCs. However, the mechanisms through which the activation of the OLMα2 interneurons regulates the activity of inhibitory interneurons targeting the CA1 pyramidal cell, and how this facilitates the increase of SC-evoked EPSPs of the CA1 pyramidal cells remain elusive.

In the CA1 region, α7 nAChR can be found on both presynaptic and postsynaptic sites of GABAergic synapses (Fabian-Fine, 2001). For this reason, the outcome of α7 nAChR activation and how it modulates OLMα2 interneuron activity is difficult to address. Activation of postsynaptic α7 nAChRs could increase the spiking frequency of OLMα2 interneurons, although, to our knowledge, OLMα2 spiking by nAChRs has not been clearly characterized, while presynaptic α7 nAChRs regulate the release of neurotransmitter by activating calcium-dependent pathways that lead to the fusion of neurotransmitter vesicles with the membrane of the neuron (Desikan et al., 2018).

**Figure 1.**
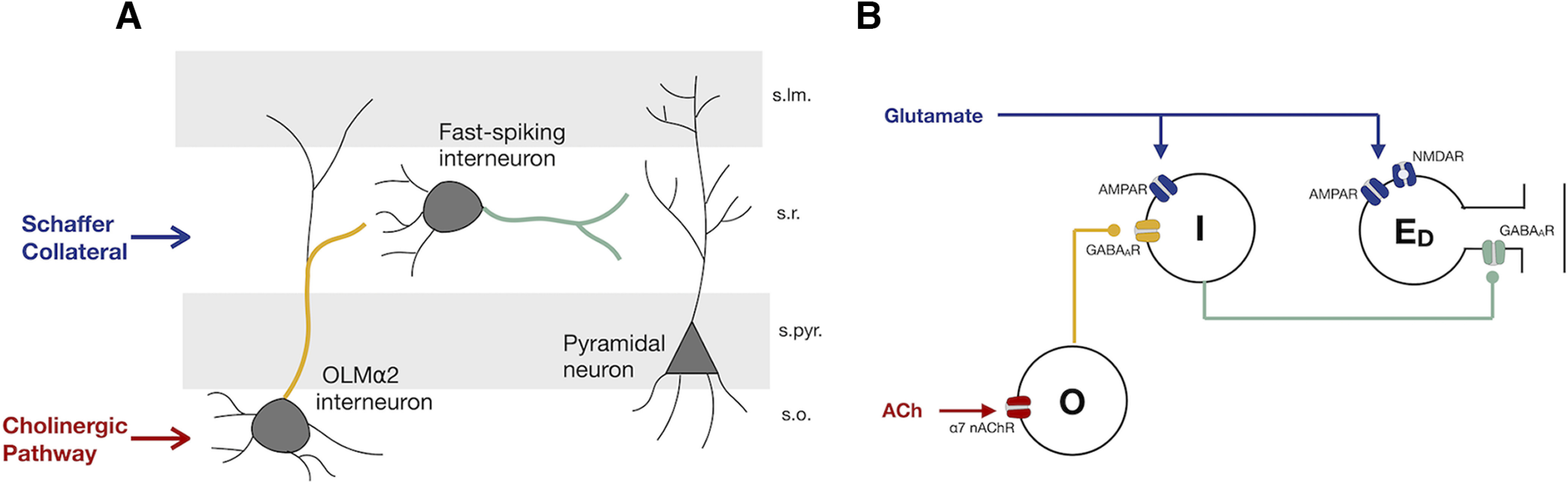
Disynaptic disinhibition circuit for nAChR-modulated long-term plasticity in the CA1. ***A***, Simplified wiring diagram of an interneuron network that mediates feedforward inhibition in the CA1 region of the hippocampus. Activation of the SC pathway leads to the activation of CA1 pyramidal cell dendrites and s.r. interneurons, which provide feedforward inhibition onto the pyramidal cell. Cholinergic activation of OLMα2 interneurons in s.o. leads to the inhibition of the s.r. interneurons, counteracting SC feedforward inhibition (Leão et al., 2012). ***B***, Minimal network to investigate plasticity induced by the pairing of cholinergic and SC activation. Glutamate activates postsynaptic AMPARs and NMDARs at the pyramidal cell E_D_ and postsynaptic AMPARs at I-cells, which in turn provide feedforward inhibition onto E_D_ by activating postsynaptic GABA_A_Rs. Cholinergic inputs act on presynaptic α7 nAChRs of O-cells, which results in GABA release of the O-cells that it is going to bind to postsynaptic GABA_A_Rs of the I-cell.

In this work, we use a minimal biophysical circuit model, driven quantitatively by *in vitro* data, to show how modulation of OLM cells (O-cells) influences the activity of fast-spiking interneurons whose GABAergic inputs are colocalized with the SC glutamatergic synapses onto a CA1 pyramidal cell dendrite, and how this promotes the induction of plasticity at the SC–CA1 synapse. We seek to determine how cholinergic activation of the OLM cells through presynaptic α7 nAChRs can downregulate the GABAergic signaling onto the pyramidal cells, and how recurrent decreased inhibitory inputs can indirectly enhance the plasticity of the excitatory SC–CA1 synapse. We thus constructed a minimal circuit consisting of a single compartment spiking model of an OLM interneuron (O-cell) with α7 nAChRs, a fast-spiking interneuron (I-cell) with AMPA and GABA_A_ receptors, and a pyramidal cell dendritic compartment (E_D_) with AMPA, NMDA, and GABA_A_ receptors. They are connected as schematically shown in Figure 1.

Overwhelming evidence suggests that most types of LTP involve calcium influx through NMDARs and subsequent changes in the properties of postsynaptic AMPA receptors (AMPARs), namely changes in their number and phosphorylation state (Collingridge et al., 1983; Barria et al., 1997; Lüscher and Malenka, 2012). To reflect these mechanisms, we use the calcium-based synaptic plasticity model (proposed by Shouval et al., 2002) to model synaptic plasticity of the SC–CA1 excitatory synapse.

In this study, we use a combination of experiments with computational modeling to put together a coherent picture of the multiple mechanisms through which concurrent disinhibition directly induces local SC–CA1 plasticity. More specifically, we show how repeated concurrent disinhibition induces LTP by mediating AMPAR trafficking. Our modeling results also put together all the pieces of the puzzle to lay out how nAChR cholinergic action on OLM interneurons, working through calcium-dependent regulation of GABA neurotransmission, can downregulate the GABAergic signaling onto CA1 pyramidal cells and induce potentiation of the SC–CA1 synapse.

## Materials and Methods

### Animals and materials

All procedures related to the use of mice followed protocols approved by the Institutional Animal Care and Use Committees of the NIEHS. ChAT-cre mice [B6;129S6-Chattm2(cre)Lowl/J], Sst-cre mice [Ssttm2.1(cre)Zjh], and floxed α7 nAChR knock-out mice [B6(Cg)-Chrna7tm1.1Ehs/YakelJ] were originally purchased from The Jackson Laboratory and then bred at the National Institute of Environmental Health Sciences (NIEHS). OLMα2-cre mice [Tg(Chrna2cre)OE29Gsat/Mmucd] were originally obtained from Mutant Mouse Resource and Research Centers and then bred at NIEHS. Mice (of either sex) were used for slice culture from day 6 to 8.

Culture media were from Sigma-Aldrich and Thermo Fisher Scientific. Adeno-associated virus (AAV) serotype 9 helper plasmid was obtained from James Wilson (University of Pennsylvania, Philadelphia, PA). The AAV vector containing floxed ChR2 (catalog #20297, Addgene) and floxed enhanced NpHR (eNpHR; catalog #26966, Addgene) were obtained from Karl Deisseroth (Stanford University, Palo Alto, CA; Gradinaru et al., 2010; Witten et al., 2010). AAV viruses were packaged with serotype 9 helper at the Viral Vector Core facility at the NIEHS.

### Brain slice culture and AAV infection

**Figure 2.**
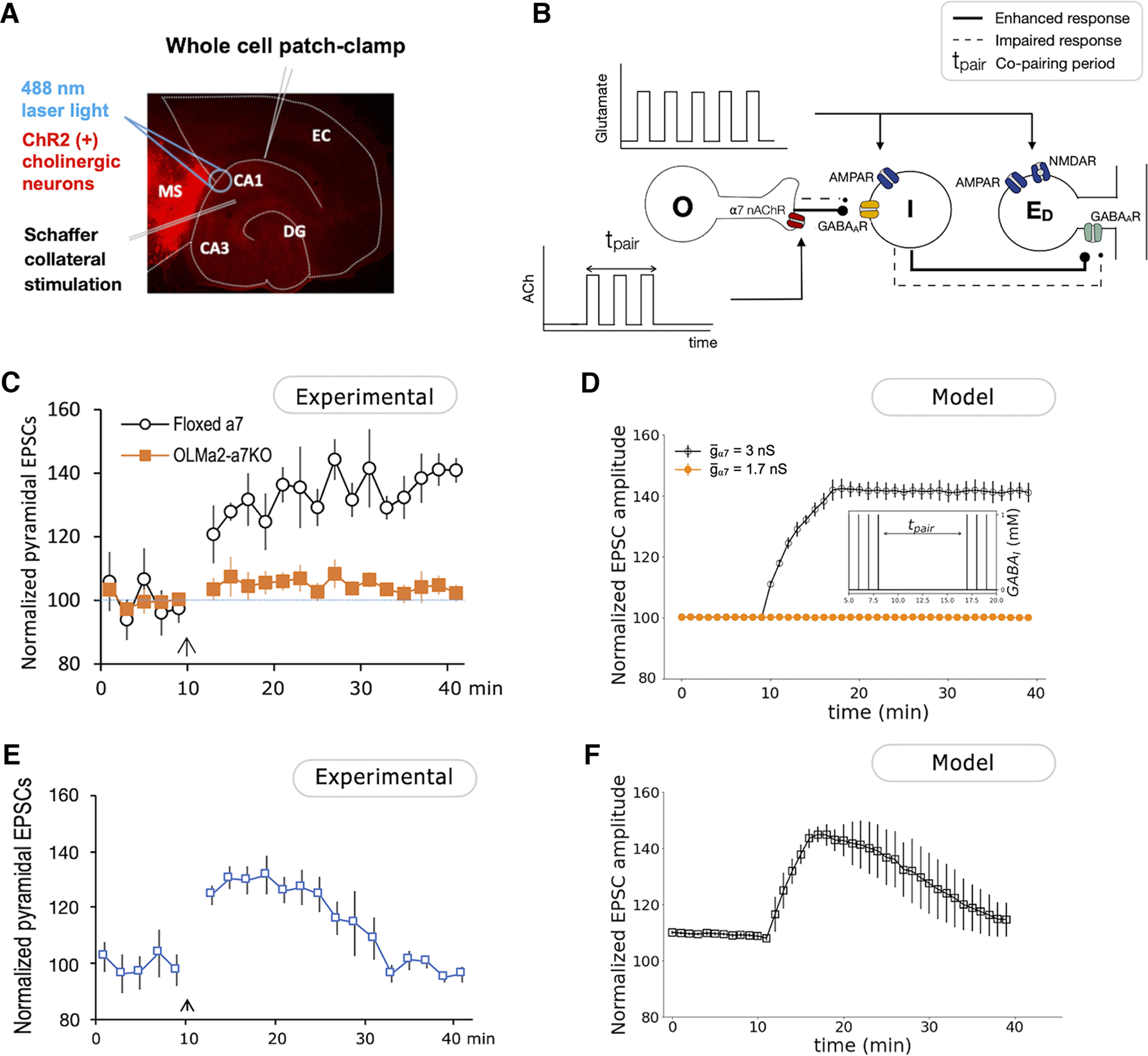
Cholinergic activation of OLM interneurons potentiates SC-evoked EPSCs. ***A***, Scheme of *in vitro* induction of cholinergic pairing-induced hippocampal synaptic plasticity. EPSCs were recorded from CA1 pyramidal neurons. Cholinergic neurons were activated via channelrhodopsin-2 that was specifically expressed in ChAT-positive neurons. The SC pathway was activated by a stimulating electrode. Adapted from the study by Gu et al. (2017). ***B***, Scheme of the minimal network used to study the role of cholinergic inputs in the potentiation of SC-evoked EPSCs. Glutamatergic inputs activate the pyramidal cell E_D_ and the fast-spiking I-cell that projects to it. Square pulses of ACh activate the O-cell during the copairing period. The neural response of O-cell, I-cell, and E_D_ when the system receives one pulse of glutamate paired or not with ACh is shown in Extended Data Figure 2-1. The release of GABA from the O-cells is calculated using the simplified model described by Equation 16. The Extended Data Figure 2-2 shows that the simplified neurotransmitter release model results in a similar synaptic activation function as the detailed model described in the study by Destexhe et al. (1998). Different ACh synaptic profiles are explored in Extended Data Figure 2-3. ***C***, Normalized SC-evoked EPSC responses from CA1 pyramidal neurons showing that the enhancement of EPSCs was impaired in hippocampal slices from mice with selective α7 nAChR knockout in OLMα2 interneurons. Adapted from the study by Gu et al. (2020). ***D***, Numerical simulation of normalized EPSC amplitude when glutamatergic inputs acting on the I-cell and E_D_ are paired with cholinergic inputs acting on the O-cell (from *t* = 10 min to *t* = 18 min). The EPSCs are calculated as the sum of postsynaptic AMPA and NMDA currents, *I*_AMPA_ and *I*_NMDA_, resulting from 10 simulations with white noise on the E_D_ membrane potential. Noisy membrane potentials of the O-cells and I-cells that induce spontaneous spiking are considered in Extended Data Figure 2-4. Normalization of the results was calculated according with the expression (100 + (EPSC – EPSCmin) · (150–100))/(EPSCmax – EPSCmin). Inset, Concentration of GABA released from fast-spiking interneurons (I), calculated according to Equation 15 (see Materials and Methods). ***E***, Normalized SC-evoked EPSC responses from CA1 pyramidal neurons showing that enhancement of EPSCs during a copairing period of 5 min. ***F***, Numerical simulation of normalized EPSC amplitude when glutamatergic inputs acting on the I-cell and E_D_ are paired with cholinergic inputs acting on the O-cell (from *t* = 10 min to *t* = 15 min).

To study the effects of cholinergic coactivation on the plasticity of SC–CA1 synapses (Fig. 2*C*,*E*), coronal septal slices (350 μm) from ChAT-cre mice and horizontal hippocampal slices from floxed α7 nAChR mice or OLMα2-cre/floxed α7 nAChR mice (350 μm) were cut with a vibratome (model VT1000S, Leica). Medial septal tissue containing cholinergic neurons was then dissected out and placed next to the hippocampus on a six-well polyester Transwell insert (Corning) and cultured there for ∼2 weeks before being used for experiments, similar to those previously described (Gu and Yakel, 2017). AAVs containing a double-floxed ChR2 construct (5 nl) were microinjected to the septal tissue with a microinjector (Drummond Scientific) on the second day of culture. To study the effects of disinhibition on the plasticity of SC–CA1 synapses (see Fig. 4*C*), horizontal hippocampal slices from Sst-cre mice were cultured and AAVs containing double-floxed eNpHR construct were microinjected to the hippocampus the next day.

10.1523/ENEURO.0389-21.2022.f2-1 Figure 2-1 ***A***, Before copairing, the α7 nAChR at OLM is not activated, and the OLM cell is not depolarized (dashed line). During copairing, OLM receives a square pulse of ACh with an amplitude of 1 mm and 5 ms of duration (solid line). The OLM is weakly depolarized (solid line). ***B***, Before copairing, there are no changes in the intracellular calcium concentration Ca*_i_* (dashed line). During copairing, calcium through α7 nAChR triggers CICR mechanisms that increase the intracellular calcium concentration of the O-cell (solid line). ***C***, An increase in intracellular calcium results in GABA release from the O-cell (GABA_O_). The neurotransmitter concentration is calculated according to the simplified model (solid line). ***D***, The release of GABA_O_ during copairing suppresses spiking of the I-cell evoked by glutamatergic activation (solid line). ***E***, Before copairing, the spiking of the I-cell is not suppressed and inhibits E_D_, which cannot depolarize a lot (dashed line). During copairing, E_D_ does not receive inhibition, only excitation from glutamatergic stimulation, and it depolarizes (solid line). Download Figure 2-1, TIF file.

10.1523/ENEURO.0389-21.2022.f2-2 Figure 2-2 Simplified neurotransmitter release model. ***A***, Square calcium pulse of 0.10 μm amplitude and 1 ms of duration. ***B***, GABA concentration elicited by a calcium pulse of 0.10 μm amplitude and 1 ms of duration computed using the detailed model of transmitter release described in the study by Destexhe et al. (1998) and using Equation 16. ***C***, Both models of GABA concentration elicit similar synaptic activation functions, *r*_G_ (described by Eq. 14 with α_G_ = 5 ms/m and β_G_ = 0.18 ms). Download Figure 2-2, TIF file.

**Figure 3.**
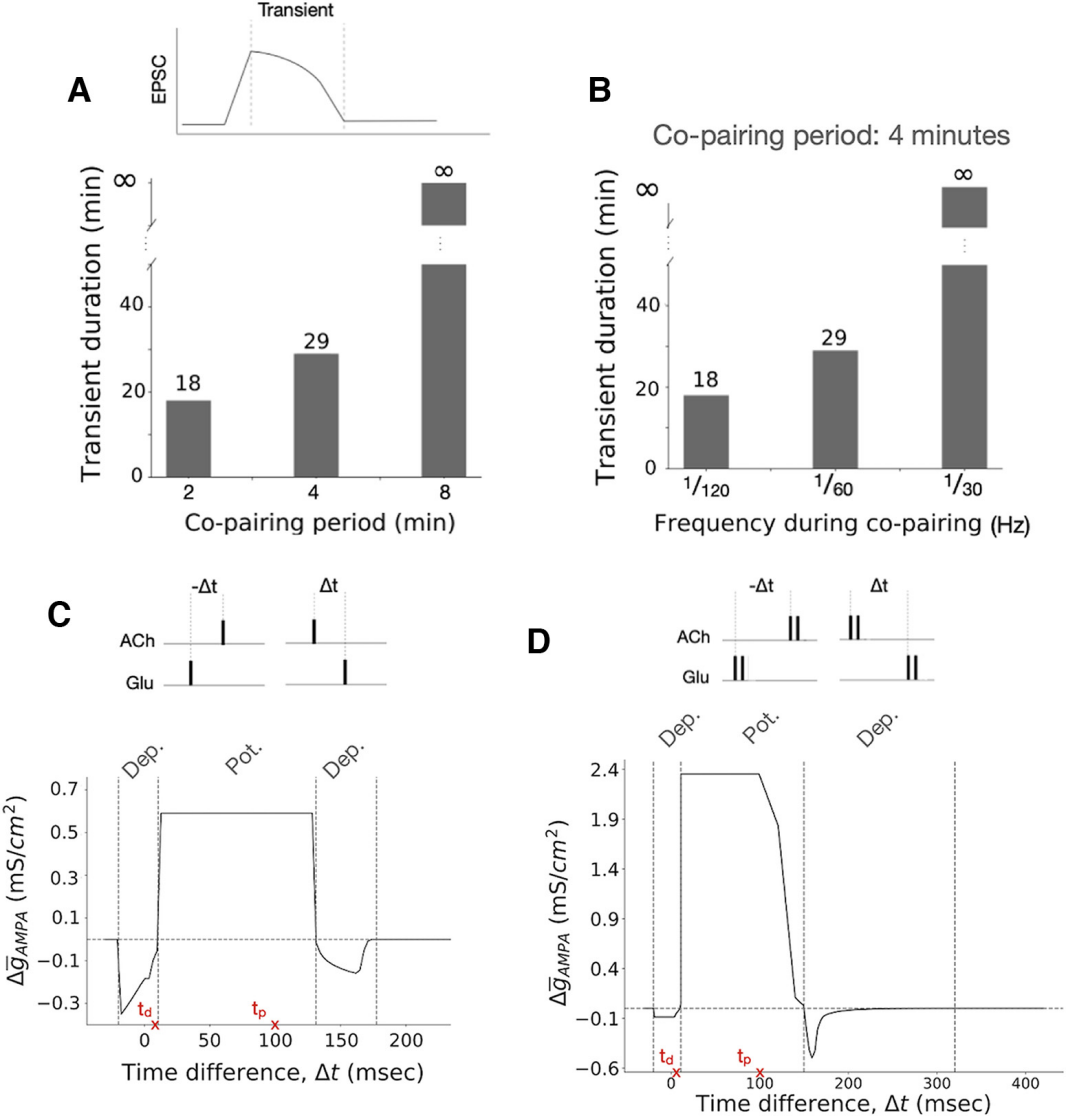
Copairing temporal parameters determines the duration and polarity of synaptic plasticity: relative timing among cholinergic and glutamatergic stimulation, the extent of the copairing period, and the frequency of stimulation (model results). ***A***, Synaptic strength transient duration is proportional to the extent of the pairing period. Here, the transient duration is defined as the time it takes the EPSC to go back to baseline after copairing is over. The I-cell and E_D_ receive a pulse of glutamate per minute. During the copairing period, the O-cell receives a pulse of ACh per minute, 100 ms before the glutamate pulses. ***B***, Synaptic strength transient duration is proportional to the ACh and glutamate pulses frequency during the copairing period. Before and after the copairing period, the I-cell and E_D_ receive a pulse of glutamate per minute. During the copairing period (4 min), the frequency changes to 1/20, 1/60, or 1/30 s, and the O-cell receives a pulse of ACh 100 ms before the glutamate pulses with the same frequency. ***C***, Relative pairing timing of single pulses provides a window of opportunity for plasticity. If glutamatergic inputs are administered within 10.4 ms *<* Δ*t* < 131.1 ms, the E_D_ excitatory synapse is potentiated. If glutamatergic inputs are administered within −19.9 ms < Δ*t* < 10.4 ms or 10.4 ms < Δ*t* < 131.1 ms, depression is induced. The change in the AMPAR conductance Δ $\bar{g}$_AMPA_ is measured 60 ms after one pairing. The relative time between cholinergic and glutamatergic activation of the network determines how efficiently the O-cells suppress spiking of the I-cells, as shown in Extended Data Figure 3-1. If noise is added to the membrane potential of E_D_, the window of depression and potentiation is not as well defined, as shown in Extended Data Figure 3-2. ***D***, Pairing multiple pulses of glutamate and ACh can change the window of opportunity for plasticity. Two pulses of glutamate and ACh with a frequency of 2 Hz are paired. If glutamatergic inputs arrive within −19.9 ms < Δ*t* < 10.9 ms or 149.9 ms < Δ*t* < 320 ms of the cholinergic inputs, depression is induced. If glutamatergic inputs are administered within 10.9 ms < Δ*t < *149.9 ms, the E_D_ excitatory synapse is potentiated. The change in the AMPAR conductance Δ $\bar{g}$_AMPA_ is measured 60 ms after one pairing. The pairing times of cholinergic and SC inputs found by Gu and Yakel (2011) to induce short-term depression and long-term potentiation at the SC–CA1 synapse (indicated with the red cross) are within our range of depression and potentiation.

10.1523/ENEURO.0389-21.2022.f2-3 Figure 2-3 Not much is known about the ACh profile in the synaptic cleft upon release from cholinergic neurons; more specifically, not much is known about the time it takes for ACh to be broken down by the cholinesterase and therefore, how long it is available to bind to the cholinergic receptors. We consider the observations made by Gu and Yakel (2011) that pairing cholinergic inputs 10 ms prior to SC stimulation induces depression of the SC–CA1 synapse, while if the cholinergic inputs are activated 100 ms prior to SC stimulation, potentiation is induced. ***A–D***, A square pulse of ACh followed by a pulse of glutamate 10 and 100 ms after will induce, respectively, depression or potentiation if the duration of the ACh pulse is equal or greater than the glutamate. ***E–H***, If ACh is described by an α function with an instantaneous rise time; the smaller the amplitude of the ACh pulse, the longer the decay time needs to be for the results to agree with those in the study by Gu and Yakel (2011). That being said, we model ACh as a square pulse with a duration of 5 ms and concentration of 1 mm, similar to glutamate. Please note that the decay and duration times, as well as the amplitude, of both the ACh and glutamate pulses serve merely as a guide to what types of neurotransmitter profiles we should consider. They are qualitative, and not quantitative, predictions of the synaptic profile of ACh. Copairing of one pulse of ACh (with different synaptic profiles) with one square pulse of glutamate (with a duration of 5 ms and amplitude of 1 mm) for a relative pairing time Δ*t* of 10 and 100 ms. ***A***, Left, One square pulse of ACh with a duration of 1 ms and concentration of 0.5 mm followed 10 ms after by a square pulse of glutamate produces no changes in the maximal conductance of AMPAR, ${\bar{g}}_{\text{AMPA}}$. Right, Similarly, If the pulse of glutamate arrives 100 ms after, no changes are induced. ***B***, Left, One square pulse of ACh with a duration of 5 ms and a concentration of 0.5 mm followed 10 ms after by a pulse of glutamate decreases the maximal conductance of AMPAR, ${\bar{g}}_{\text{AMPA}}$. Right, If the pulse of glutamate arrives 100 ms after, potentiation is induced. ***C***, Left, One square pulse of ACh with a duration of 1 ms and concentration of 1 mm followed 10 ms after by a square pulse of glutamate produces no changes in the maximal conductance of AMPAR, ${\bar{g}}_{\text{AMPA}}$. Right, Similarly, if the pulse of glutamate arrives 100 ms after, no changes are induced. ***D***, Left, One square pulse of ACh followed 10 ms after by a pulse of glutamate with the same characteristics (duration of 5 ms and 1 mm concentration) decrease the maximal conductance of AMPAR, ${\bar{g}}_{\text{AMPA}}$. Right, If the pulse of glutamate arrives 100 ms after, potentiation is induced. ***E***, Left, One pulse of ACh with an amplitude of 0.39 mm and a decay time constant of 1 ms followed 10 ms after by a square pulse of glutamate induces no changes in ${\bar{g}}_{\text{AMPA}}$. Right, Similarly, if the pulse of glutamate arrives 100 ms after, no changes are induced. ***F***, Left, One pulse of ACh with an amplitude of 0.39 mm and a decay time constant of 4 ms followed 10 ms later by a square pulse of glutamate depresses ${\bar{g}}_{\text{AMPA}}$. Right, If the pulse of glutamate arrives 100 ms after, potentiation is induced. ***G***, Left, One pulse of ACh with an amplitude of 1 mm and a decay time constant of 1 ms followed 10 ms later by a square pulse of glutamate provokes a decrease in ${\bar{g}}_{\text{AMPA}}$. Right, If the pulse of glutamate arrives 100 ms after, no changes are induced. ***H***, Left, One pulse of ACh with an amplitude of 1 mm and a decay time constant of 4 ms followed 10 ms later by a square pulse of glutamate depresses ${\bar{g}}_{\text{AMPA}}$. Right, If the pulse of glutamate arrives 100 ms after, potentiation is induced. Download Figure 2-3, TIF file.

**Figure 4.**
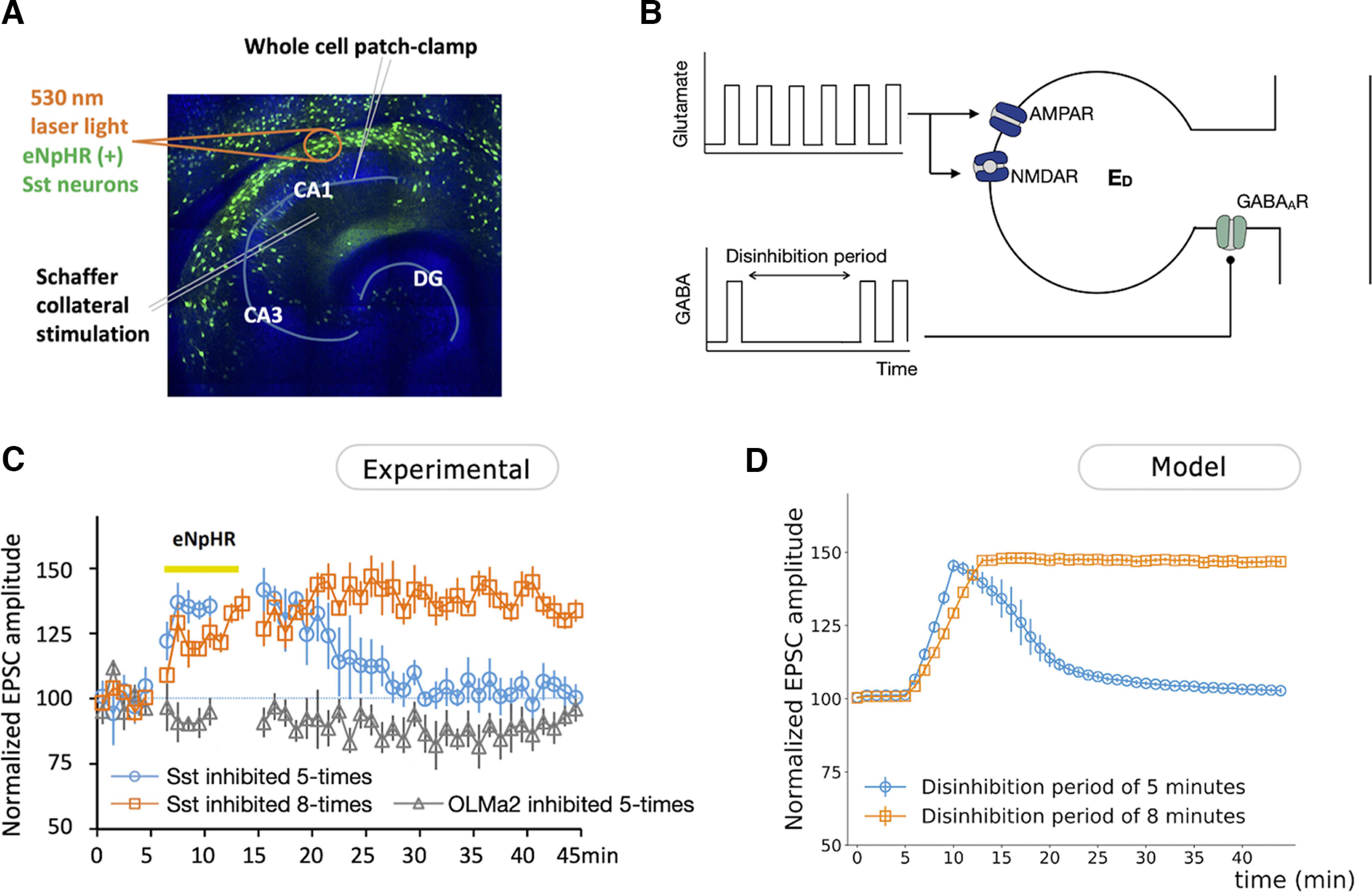
Disinhibition of CA1 pyramidal cell facilitates the induction of hippocampal synaptic plasticity. ***A***, Scheme of *in vitro* induction of hippocampal synaptic plasticity through concurrent Sst inhibition. EPSCs were recorded from CA1 pyramidal neurons. Sst neurons were inhibited via eNpHR that was specifically expressed in Sst-positive neurons. The SC pathway was activated by a stimulating electrode. ***B***, Schematic representation of a CA1 pyramidal neuron dendritic compartment E_D_ with postsynaptic GABA_A_, AMPA, and NMDA receptors used to study the disinhibitory mechanisms for induction of plasticity at the SC–CA1 excitatory synapse. The dendritic compartment of the pyramidal cell receives one pulse of both glutamate and GABA per minute, except during the disinhibition period, where it only receives pulses of glutamate. The GABA pulse, presumably from the I-cell, is described by a square function with similar amplitude and duration as the glutamate pulse (see Materials and Methods; Extended Data Figs. 4-1, 4-2, justification). Glutamate binds to the excitatory AMPA and NMDA receptors, while GABA binds to the inhibitory GABA_A_ receptor. The synaptic currents and membrane potential of E_D_ when a pulse of glutamate is paired (or not) with a pulse of GABA are shown in Extended Data Figure 4-3. ***C***, Experimental measurements showing the effects of inhibition of Sst and OLM*α*2 interneurons in s.o. on SC-evoked EPSCs (*n* = 5 slices for each group). Inhibition of Sst interneurons from *t* = 5 min to *t* = 10 min enhanced the SC-evoked EPSC amplitude of the CA1 pyramidal cell, followed by a return to the baseline after the inhibition period (blue line). Inhibition of Sst interneurons from *t* = 5 min to *t* = 13 min increased SC-evoked amplitude EPSCs, which remained potentiated after the inhibition period (orange line). ***D***, Numerical simulation of normalized EPSCs of E_D_ for a disinhibition period of 5 min (from *t* = 5 min to *t* = 10 min) and 8 min (from *t* = 5 min to *t* = 13 min). Normalization of the results was calculated according with the expression (100 + (EPSC – EPSCmin) · (150–100))/(EPSCmax – EPSCmin).

10.1523/ENEURO.0389-21.2022.f2-4 Figure 2-4 ***A***, Time evolution of the membrane potential of the O-cell, I-cell, and E_D_ with noisy background currents when cholinergic inputs are paired with SC inputs, and resultant EPSCs. ***B***, Mean trace of normalized EPSCs after 10 simulations. Adding a noisy background current to the O-cell and I-cell induces spontaneous spiking. Copairing cholinergic and glutamatergic inputs from *t* = 10 min to *t* = 18 min induces potentiation of the pyramidal cell EPSC. The O-cell releases GABA when the intracellular calcium concentration is high enough (Eq. 16) and when the cell spikes (Eq. 15). All the remaining parameters are identical to the ones used to produce Figure 6. Noise was incorporated by adding a stochastic term $\sqrt{dt}\zeta$, where ζ is a random Gaussian variable with a mean of μ = 0 and an SD of σ (=1.1, 0.1, and 0.2 for the O-cells, I-cells, and E_D_, respectively), to the Euler equations describing the *V*_x_. Normalization of the results was calculated according with the expression (100 + (EPSC – EPSCmin) · (150 – 100))/(EPSCmax – EPSCmin). Download Figure 2-4, TIF file.

### Whole-cell patch-clamp recordings

SC–CA1 EPSCs were recorded from hippocampal CA1 pyramidal neurons under whole-cell patch clamp, similar to recordings described in the studies by Gu and Yakel (2011, 2017). Briefly, 2–3 weeks after culturing, the slices were removed from transwell inserts and put into a submerged chamber, continuously perfused with 95% O_2_/5% CO_2_ balanced ACSF (in mm: 122 NaCl, 2.5 KCl, 2 MgCl_2_, 2 CaCl_2_, 1.2 NaH_2_PO_4_, 25 NaHCO_3_, and 25 glucose) at room temperature. EPSCs were recorded at −60 mV under voltage clamp through a glass pipette filled with an internal solution (in mm: 130 potassium gluconate, 2 MgCl_2_, 3 MgATP, 0.3 Na_2_GTP, 10 KCl, 10 HEPES, and 1 EGTA) at pH ∼7.2–7.3 and osmolarity of ∼280–290 mOsm. Whole-cell patch-clamp recordings were performed with a Multiclamp 700B amplifier (Molecular Devices). Data were digitized with an analog-to-digital signal converter (Digidata 1550) and collected with Clampex. The amplitudes of EPSCs were analyzed with Clampfit, and graphs were drawn with Excel. The amplitudes were normalized to the mean of the 10 min baseline recording before cholinergic pairing or disinhibition pairing. Values were presented as the mean ± SEM.

EPSCs were evoked every 60 s by stimulating the SC pathway with an electrode placed in the stratum radiatum through a stimulator (model S88X, Grass). The stimulation intensity was 1–10 μA for 0.1 ms. To study the effects of cholinergic coactivation on SC–CA1 synaptic plasticity (Fig. 2*C*,*E*), cholinergic terminals in the hippocampus were optogenetically activated (10 pulses at 10 Hz, 1 s before SC stimulation) through ChR2 that was selectively expressed in ChAT-cre-positive (cholinergic) neurons. ChR2 was activated with 488 nm laser light (5 mW, 20 ms) through a 40× objective over CA1 stratum oriens (s.o.) near the septum with an spinning disk confocal microscope (Andor Technology). To examine the effects of disinhibition on SC–CA1 synaptic plasticity (see Fig. 4*C*), somatostatin (Sst)-positive neurons were inhibited optogenetically through eNpHR which was activated through a 40× objective over CA1 stratum oriens with 530 nm laser light (20 mW) for 1 s flanking SC stimulation.

The amplitudes of EPSCs were analyzed with Clampfit, and graphs were drawn with Excel. The amplitudes were normalized to the mean of the 5 min baseline recording before cholinergic pairing or disinhibition pairing. Values were presented as the mean ± SEM EPSC amplitudes at 5 and 30 min after pairing were compared with the amplitude at 5 min before pairing. The effect at 5 min after pairing was considered to be a short-term effect, and the effect at 30 min after pairing was considered to be a long-term effect. Recordings were performed in five slices from three individual mice in each group. Statistical significance was tested by Student’s *t* test. The sample size was estimated by Student’s *t* test with an expected effect of 40% change, an expected SD of 15%, and an 80% confidence interval width.

### Model

The minimal network used in this study consists of an O-cell, a fast-spiking I-cell, and a pyramidal cell. All the cells in the network are modeled as point neurons. Since we are interested in the local changes at the SC–CA1 synapse, the pyramidal cell is represented by a dendritic compartment (E_D_). The cells of the network are connected through feedforward connections. Although recurrent connections from the CA1 pyramidal cell and the fast-spiking interneurons may exist, adding this connection did not change our results. Adding connections between the CA1 pyramidal cell and the OLM interneuron also did not significantly alter our results. Therefore, we did not include synapses between the CA1 pyramidal and the OLM cells in our model. Our modeling choice is further supported by experimental studies showing that the IPSC elicited by an OLM interneuron has a small amplitude at the soma of CA1 pyramidal cells since these synapses are on the distal parts of the dendritic tree (Maccaferri, et al., 2000), and that an action potential in CA1 pyramidal cells is insufficient to make the OLM cell membrane potential (*V*_m_) cross the action potential threshold (Ali et al., 1998; Müller and Remy, 2014). Although repeated firing of CA1 pyramidal cells with theta frequency can facilitate excitatory inputs onto OLM, a theta activation protocol of the CA1 cell is beyond the scope of this article.

### Neuron dynamics models

The O-cells and I-cells are modeled following the Hodgkin–Huxley formalism [Hodgkin and Huxley, 1952; transient (*I*_Na_), delayed rectifier potassium (*I*_K_), and leak (*I*_leak_)], with synaptic currents (*I*_syn_). Its *V*_m_ is described as follows:

(1) $$C_{\text{m}}\frac{dV_{\text{m}}}{dt}=-I_{\text{leak}}-I_{\text{K}}-I_{\text{Na}}-I_{\text{syn}},$$

where *C*_m_ is the membrane capacitance. The *I*_leak_, *I*_K_, and *I*_Na_ currents are given by:

(2) $$I_{\text{leak}}=g_{\text{leak}}\left(V_{\text{m}}-E_{\text{leak}}\right),$$

(3) $$I_{\text{K}}={\bar{g}}_{\text{K}}n^{4}\left(V_{\text{m}}-E_{\text{K}}\right),$$

(4) $$I_{\text{Na}}={\bar{g}}_{\text{Na}}m^{3}h(V_{\text{m}}-E_{\text{Na}}),$$

where ${\bar{g}}_{i}$ and *E_i_* are, respectively, the maximal conductance and reversal potential of channel *i* (*i* = leak, K, Na), and *m*, *h*, and *n* are gating variables that obey the following differential equation:

(5) $$\frac{dx}{dt}=\alpha_{x}\left(1-x\right)-\beta_{x}x,$$

where α*_x_* and β*_x_* are voltage-dependent rate constants.

Following Rotstein et al. (2005), we included an applied current (*I*_app_) = −260 pA, a persistent Na current (*I*_p_), and a hyperpolarization-activated inward current (*I*_h_*;* with a slow and fast component) on the O-cells, as follows:

(6) $$I_{\text{p}}={\bar{g}}_{\text{p}}p\left(V_{\text{m}}-E_{\text{Na}}\right),$$

(7) $$I_{\text{h}}={\bar{g}}_{\text{h}}\left(0.65h^{f}+0.35h^{s}\right)\left(V_{\text{m}}-E_{\text{h}}\right).$$

While the gate variable *p* obeys Equation 5, *h^f^* and *h^s^* are described by the following equation:

(8) $$\frac{dx}{dt}=\frac{x_{\infty }\left(V_{\text{m}}\right)-x}{\tau_{x}(V_{\text{m}})},$$

where *x*_∞_ is the voltage-dependent steady state and τ*_x_* is the time constant. Definitions for the *α_x_*, *β_x_*, *x_∞_*, and τ*_x_* for each of the dynamic variables are as follows.

For the O-cells:

$$\alpha_{n}=\frac{-0.01\left(V_{\text{m}}+27\right)}{\exp\left(-0.1\left(V_{\text{m}}+27\right)\right)-1},$$

βn=0.125 exp(−Vm + 3780),

$$\alpha_{\text{m}}=\frac{-0.1\left(V_{\text{m}}+23\right)}{\exp\left(-0.1\left(V_{\text{m}}+23\right)\right)-1},$$

$$\beta_{m}=4\text{ exp}(-\frac{V_{\text{m}}+48}{18}),$$

$$\alpha_{\text{h}}=0.07\text{ exp}(-\frac{V_{\text{m}}+37}{20}),$$

$$\beta_{\text{h}}=\frac{1}{\text{exp}(-0.1\left(V_{\text{m}}+7\right)+1)},$$

$$\alpha_{p}=\frac{1}{0.15(1+\text{exp}(-\frac{V_{\text{m}}+38}{6.5}))},$$

$$\beta_{p}=\frac{\exp\left(-\frac{V_{\text{m}}+38}{6.5}\right)}{0.15(1+\text{exp}(-\frac{V_{\text{m}}+38}{6.5}))},$$

$$h_{\infty }^{f}=\frac{1}{1+\exp\left(\frac{V_{\text{m}}+79.2}{9.78}\right)},$$

$$\tau_{h}^{f}=\frac{0.51}{\exp\left(\frac{V_{m}-1.7}{10}\right)+\text{exp}(-\frac{V_{\text{m}}+340}{52})}+1,$$

$$h_{\infty }^{s}=\frac{1}{{\left[1+\exp\left(\frac{V_{\text{m}}+2.83}{15.9}\right)\right]}^{58}},$$

$$\tau_{h}^{s}=\frac{5.6}{\exp\left(\frac{V_{m}-1.7}{14}\right)+\text{exp}(-\frac{V_{\text{m}}+260}{43})}+1.$$

For the I-cells:

$$\alpha_{\text{n}}=\frac{0.032\left(V_{\text{m}}+52\right)}{1-\text{exp}\left(-\frac{V_{\text{m}}+52}{5}\right)},$$

$$\beta_{\text{n}}=0.5\exp\left(-\frac{V_{\text{m}}+57}{40}\right),$$

$$\alpha_{\text{m}}=\frac{0.32\left(V_{\text{m}}+54\right)}{1-\text{exp}\left(-\frac{V_{\text{m}}+54}{4}\right)},$$

$$\beta_{\text{m}}=\frac{0.28\left(V_{\text{m}}+27\right)}{\exp\left(\frac{V_{\text{m}}+27}{5}\right)-1},$$

$$\alpha_{\text{h}}=0.128\text{exp}\left(-\frac{V_{\text{m}}+50}{18}\right),$$

$$\beta_{\text{h}}=\frac{4}{1+\text{exp}(-\frac{V_{\text{m}}+27}{5})}.$$

The parameter values used in the simulations are the ones presented in Table 1.

**Table 1:** Parameters of pyramidal cell, OLM interneuron, and fast-spiking interneuron dynamics

Parameter	Value
O-cells	
*C*_m_	100 pF
*g*_leak_	50 nS
*E*_leak_	−70 mV
${\bar{g}}_{\text{K}}$	1100 nS
*E*_K_	−90 mV
${\bar{g}}_{\text{Na}}$	5200 nS
*E*_Na_	55 mV
${\bar{g}}_{\text{p}}$	50 nS
${\bar{g}}_{\text{h}}$	145 nS
*E*_h_	−20 mV
I-cells	
*C*_m_	100 pF
*g*_leak_	10 nS
*E*_leak_	−67 mV
${\bar{g}}_{\text{K}}$	8000 nS
*E*_K_	−100 mV
${\bar{g}}_{\text{Na}}$	10,000 nS
*E*_Na_	50 mV
E_D_	
*C*_m_	100 pF
*g*_leak_	1 nS
*E*_leak_	−68 mV

All the parameter values and expressions here described were taken from the study by Rotstein et al. (2005), considering a surface area of 1 × 10^−4^ cm^2^, except for the reversal potential of the leakage current of the OLM, which was set to have the resting potential of the OLM cells at −60 mV, as reported in the study by Leão et al. (2012).

Since we are interested in studying local synaptic changes of the SC–CA1 synapse, we use the following equation to describe the activity of the pyramidal cell dendritic compartment:

(9) $$C\frac{dV_{\text{E}}{}_{\text{D}}}{dt}=-I_{\text{leak}}-I_{\text{syn}}.$$

The parameters *C*, *g*_leak_, and *E*_leak_ were set to 100 pF, 1 nS, and −68 mV, respectively.

For the simulations presented in Figure 2*D*, noise was added to the dendritic compartment E_D_ to allow direct comparison with the experimental results portrait in Figure 2*C*. In addition to E_D_, white noise was added to the O-cells and I-cells to study plasticity induction when these cells show spontaneous spiking (Extended Data Figs. 2-1, 2-2, 2-3, 3-1, 3-2, 4-1). Since we used the Euler method to solve the differential equations describing *V*_O_, *V*_I_, and *V*_ED_, ( $V_{x}\left[i+1\right]=V_{x}\left[i\right]+dt\frac{dV_{x}}{dt}$) noise was incorporated by adding a stochastic term $\sqrt{dt}\zeta$ ( $V_{x}\left[i+1\right]=V_{x}\left[i\right]+dt\frac{dV_{x}}{dt}+\sqrt{dt}\zeta$), where ζ is a random Gaussian variable with mean μ=0 and SD σ(=1.1, 0.1, and 0.3 for the O-cells, I-cells, and E_D_ cells, respectively).

### Synaptic models

The O-cell model includes a current mediated by α7 nAChR channels, which in the real OLM neurons are presynaptic to the O-cell to I-cell synapse. The description of the current used is an adaptation of the model proposed in the study by Graupner et al. (2013), and it is given by the following equation:

(10) $$I_{\alpha 7}={\bar{g}}_{\alpha 7}r_{\alpha 7}\left(V_{\text{m}}-E_{\alpha 7}\right),$$

where ${\bar{g}}_{\alpha 7}\;$ is the maximal conductance of the α7 nAChR channel, and *E*_α7_ is the reversal potential. The opening gate variable r_α7_ is described by Equation 8, with τ_rα7_ (= 5 ms) constant and r_(α_7)∞ given by the following:

(11) $$r_{\left(\alpha_{7}\right)\infty }=\frac{{\left[\text{ACh}\right]}^{n}}{{\text{EC}}_{50}^{n}+{\left[\text{ACh}\right]}^{n}},$$

where *n* is the Hill’s coefficient of activation.

The I-cell has excitatory AMPA and inhibitory GABA_A_ synaptic currents, described by the following set of equations:

(12) $$I_{\text{GABA}}{}_{{}_{{}_{\text{A}}}}={\bar{g}}_{G}r_{G}\left(V_{\text{m}}-E_{\text{G}}\right),$$

(13) $$I_{\text{AMPA}}={\bar{g}}_{\text{AMPA}}r_{A}\left(V_{\text{m}}-E_{\text{A}}\right).$$

The gating variable *r_x_* is, as described in the study by Destexhe et al. (1998), given by the following:

(14) $$\frac{dr_{x}}{dt}=\alpha_{x}\left[T\right]\left(1-r_{x}\right)-\beta_{x}r_{x},$$

where α*_x_* and β*_x_* are the opening and closing rates of the receptor channel, and [*T*] is the concentration of the neurotransmitter that is available for binding.

The GABA released by the I-cell is described by using the Destexhe et al. (1998) simplified neurotransmitter release model. The intervening reactions in the release process are considered to be fast: a presynaptic action potential elicits a rapid influx of calcium, leading to the activation of transmitter-containing vesicles and neurotransmitter release. A stationary relationship between presynaptic voltage and neurotransmitter release is deduced by fitting the model to experimental results. The following equation gives the neurotransmitter release as a function of the presynaptic voltage:

(15) $${\left[\text{GABA}\right]}_{I}=\frac{T_{\text{max}}}{1+\exp\left(-\frac{V_{\text{m}}-V_{\text{p}}}{K_{\text{p}}}\right)},$$

where *T*_max_= 1 mm is the maximal neurotransmitter concentration, *K*_p_ = 5 mV gives the steepness of the function, and *V*_p_ = 2 mV sets the value at which the function is half-activated. These parameters were directly taken from the study by Destexhe et al. (1998).

Spiking of the OLM cells directly because of the nAChR activation has not been clearly characterized. Experimentally measured nicotinic responses of OLM cells are small (Leão et al., 2012), and, although they may modulate the firing rate of the neuron, it is unlikely they are causing spiking on their own (Fig. 2*D*). For that reason, we consider that GABA release by the O-cell results from the activation of presynaptic α7 nAChR on O–I GABAergic synapses.

Experimental studies revealed that the activation of α7 nAChRs trigger intracellular calcium rise and calcium-dependent signaling pathways—in particular calcium-induced calcium release (CICR) from intracellular stores—that enhance the release of neurotransmitter at presynaptic terminals (Tsuneki et al., 2000; Dajas-Bailador et al., 2002; Griguoli and Cherubini, 2012). To avoid the detailed computation of the mechanisms whereby calcium leads to exocytosis, we assume a sigmoid relationship between intracellular calcium and transmitter concentration given by the following:

(16) $${\left[\text{GABA}\right]}_{O}=\frac{T_{\text{max}}}{1+\text{exp}(-\frac{{\text{Ca}}_{i}-{\text{Ca}}_{p}}{K_{(\text{Ca})p}})},$$

where *T*_max_= 1 mm is the maximal neurotransmitter concentration, *K*_(Ca)_*_p_* = 1 × 10^−6 mm^ gives the steepness of the function, and Ca*_p_* = 4 × 10^−5 mm^ sets the value at which the function is half-activated. These parameters were chosen so that a pulse of calcium elicits GABA release with approximately the same characteristics (amplitude and duration) as the detailed model of transmitter release in the study by Destexhe et al. (1998; Extended Data Fig. 2-2, compare detailed and simplified models of neurotransmitter release). Note that although Ca*_p_* is below the calcium resting values typically observed, in our model the calcium concentration decays to zero, similar to that in the studies by Graupner and Brunel (2005, 2012), Higgins et al. (2014), and Shouval et al. (2002).

The passive dendritic compartment of the pyramidal cell E_D_ is modeled using synaptic GABA_A_, AMPA, and NMDA currents. The GABA_A_ and AMPA currents are given by Equations 12 and 13, respectively. The following equation describes the NMDA current:

(17) $$I_{\text{NMDA}}={\bar{g}}_{N}r_{N}B\left(V_{\text{m}}\right)\left(V_{\text{m}}-E_{\text{N}}\right),$$

where *r_N_* is the gating variable described by Equation 14. Because of the presence of an Mg^2+^ block, the NMDA channels have an additional voltage-dependent term, *B*(*V*_m_), defined as follows:

(18) $$B\left(V_{\text{m}}\right)=\frac{1}{\begin{array}{c} 1+\exp\left(-0.062V_{\text{m}}\right)\frac{\left[{\text{Mg}}^{2}{}^{+}\right]}{3.57} \end{array}}.$$

The parameters α_A_, β_A_, E_A_, α_N_, β_N_, E_N_, [Mg^2+^], α_G_, β_G_, and E_G_ were estimated by Destexhe et al. (1998) by fitting the models of postsynaptic AMPA, NMDA and GABA_A_ currents to experimental data. Regarding the synaptic currents of E_D_, the maximal conductances of AMPA and NMDA receptors were chosen such that at *V* = −70 mV, a glutamate pulse of 1 mm and 10 ms duration evoked AMPA and NMDA currents with amplitudes of 240 and 40 pA, respectively (Andrásfalvy et al., 2003). The maximal conductance of GABA_A_ receptors was chosen such that at *V* = 0 mV a pulse of GABA with 1 ms duration and a concentration of 1 mm evokes a current with an amplitude of 500 pA (Schulz et al., 2018). For the I-cell, the AMPA receptor maximal conductance value is such that one pulse of glutamate coming from the SC evokes a volley of action potentials. Concerning the α7 nAChR postsynaptic current, the parameters EC_50_, τ_rα7_, and *n* were taken from the study by Graupner et al. (2013), where the kinetics of α7 nAChR is described. The parameter E_α7_ was deduced from the study by Castro and Albuquerque (1995), and gα7 was chosen such that activation of the α7 nAChR by a pulse of ACh evokes a current of 35 pA (Leão et al., 2012). The values of the parameters can be found in Table 2.

**Table 2:** Parameter values of synaptic currents *I*_AMPA_, *I*_NMDA_, *I*_GABAA_, and *I*_α7_

Parameter	Value	Reference
α_A_	1.1 ms^−1^ mM^−1^	Destexhe et al. (1998)
β_A_	0.19 ms^−1^	Destexhe et al. (1998)
${\bar{g}}_{\text{AMPA}}$	7*, 4^†^ nS	Andrásfalvy et al. (2003)
E_A_	0 mV	Destexhe et al. (1998)
[Mg^2+^]	1 mm	Destexhe et al. (1998)
α*_N_*	0.072 ms^−1^ mM^−1^	Destexhe et al. (1998)
β*_N_*	6.6 × 10^−3^ ms^−1^	Destexhe et al. (1998)
${\bar{g}}_{\text{N}}$	25 nS	Andrásfalvy et al. (2003)
*E*_N_	0 mV	Destexhe et al. (1998)
α*_G_*	5 ms^−1^ mM^−1^	Destexhe et al. (1998)
β*_G_*	0.18 ms^−1^	Destexhe et al. (1998)
${\bar{g}}_{\text{G}}$	14*, 7^†^ nS	Schulz et al. (2018)
E_G_	−80 mV	Destexhe et al. (1998)
*E*_α_ *_7_*	0 mV	Castro and Albuquerque (1995)
${\bar{g}}_{\text{α7}}$	3 nS	Leão et al. (2012)
EC_50_	80 × 10^–3 ^mm	Graupner et al. (2013)
τ*_rα7_*	5 ms	Graupner et al. (2013)
*n*	1.73	Graupner et al. (2013)
*T*_max_	1 mm	Destexhe et al. (1998)
*K*_p_	5 mV	Destexhe et al. (1998)
*V*_p_	2 mV	Destexhe et al. (1998)
*K*_(Ca)p_	1 × 10^–6^ mm	Materials and Methods
Ca_p_	4 × 10^–5^ mm	Materials and Methods

*Values refer to the conductance of postsynaptic channels on the fast-spiking interneurons.

^†^Values refer to the conductances of the dendritic compartment E_D_.

### CICR mechanism

Calcium entry through α7 nAChRs initiates calcium release from internal stores (Tsuneki et al., 2000; Dajas-Bailador et al., 2002; Griguoli and Cherubini, 2012). The calcium concentration in the cytosol of OLM cells (Ca*_i_*) is described by the following equation:

(19) $$\frac{d{\text{Ca}}_{i}}{dt}=-\xi \prime \alpha \prime I_{\alpha 7}+w_{\infty }^{3}({\text{Ca}}_{\text{IS}}-{\text{Ca}}_{i})-\frac{\text{Ca}}{\tau_{\text{Ca}}},$$

where ξ′=2.1 × 10^−6 mm^/(ms pA) is a parameter that converts current into concentration, α′ = 0.05 reflects the 5% calcium permeability of the α7 nAChRs (Vernino et al., 1994), and τ_Ca_ is the calcium decay constant. The parameter ξ′ was chosen so that the intracellular calcium concentration is of the same order of magnitude as observed experimentally in the study by Sabatini et al. (2002; i.e., in the 0.1 μm range). The parameter τ_Ca_ was taken directly from the same study. Ca_IS_ represents the calcium concentration of the internal stores given by the following:

(20) $$\frac{d{\text{Ca}}_{\text{IS}}}{dt}=-w_{\infty }^{3}({\text{Ca}}_{\text{IS}}-{\text{Ca}}_{i})-\frac{\left({\text{Ca}}_{\text{IS}}-0.44\text{ × }10^{-3}\right)}{\tau },$$

where τ (= 10 ms) is the calcium decay constant, and *w*_∞_ is the open probability of calcium-permeable channels on the internal store, given by the following:

(21) $$w_{\infty }=\frac{{\text{Ca}}_{i}}{{\text{Ca}}_{i}+k_{\text{d}}},$$

where *k*_d_ (= 2 × 10**^−^**^4 mm^) is the half-activation of the function. The model assumes three calcium binding sites (Young and Keizer, 1992) and a calcium concentration at the internal stores of 0.44 μm at rest (this value can be different as long as it is bigger than the intracellular calcium concentration Ca*_i_*). Please note that the CICR mechanism described is a simplification of the model proposed by Rinzel **(**1985), where we limit the model to account for the calcium activation sites of the calcium-permeable IP_3_ receptors on the endoplasmic reticulum.

### Model of synaptic plasticity

To study plasticity induction at the SC–E_D_ synapse, we use a calcium-based synaptic plasticity model based on the study by Shouval et al. (2002). We assume that changes in the AMPA receptor conductance reflect changes in the strength of the excitatory SC–CA1 synapse. Our synaptic plasticity model is formulated as follows:

(22) $$\frac{d{\bar{g}}_{\text{AMPA}}}{dt}=\eta \left(\text{Ca}\right)\left(\text{Ω}\left(\text{Ca}\right)-\sigma \left({\bar{g}}_{\text{AMPA}}-g_{0}\right)\right),$$

where *σ* is a decay constant and *g*_0_ (= 4 nS) is the value of the maximal conductance of the AMPAR at *t* = 0. The variable *η* is a calcium-dependent learning rate described by Equation 23, and Ω determines the sign magnitude of synaptic plasticity as a function of the intracellular Ca levels (Eq. 24).

(23) $$\eta \left(\text{Ca}\right)={\left(\frac{{\text{P}}_{1}}{{\text{P}}_{2}+{\text{Ca}}^{{\text{P}}_{3}}}+{\text{P}}_{4}\right)}^{-1},$$

(24) $$\text{Ω}\left(\text{Ca}\right)=\gamma_{↑}\frac{\exp\left(900\left(\text{Ca}-\theta_{↑}\right)\right)}{1+\exp\left(900\left(\text{Ca}-\theta_{↑}\right)\right)}-\gamma_{↓}\frac{\exp\left(900\left(\text{Ca}-\theta_{↓}\right)\right)}{1+\exp\left(900\left(\text{Ca}-\theta_{↓}\right)\right)}.$$

The parameters θ*_↑_* and θ*_↓_* define the potentiation and depression onset (i.e., the calcium levels that trigger the removal and insertion of AMPAR in the membrane, respectively), and ${\text{γ}}_{↑}$ and ${\text{γ}}_{↓}$ represent the maximal insertion and removal rate of the AMPARs from the membrane. Please note that on the original model, the parameters θ_↑_ and θ_↓_ are represented by θ_p_ and θ_d_, and define the potentiation and depression threshold, respectively, but, as will be evident in the Results section, we find that this terminology can be misleading (i.e., we show that crossing these levels is necessary but not sufficient for potentiation).

We assume that the primary source of Ca^2+^ in E_D_ is the calcium flux entering the cell through the NMDA receptor channels. The intracellular Ca^2+^ concentration evolves according to the following equation:

(25) $$\frac{d\text{Ca}}{dt}=-\xi \alpha I_{\text{NMDA}}-\frac{\text{Ca}}{\tau_{\text{Ca}}},$$

where ξ is a parameter that converts current into concentration, α = 0.1 refers to the fact that only ∼10% of the NMDA current is composed of calcium ions (Burnashev et al., 1995), and τ_Ca_ (= 12 ms) refers to the calcium decay constant. The parameter ξ was chosen so that the intracellular calcium concentration is of the same order of magnitude as observed experimentally in the study by Sabatini et al. (2002). The parameter τ_Ca_ was taken directly from the same study. P_1_, P_2_, P_3_, and P_4_ were chosen to have a calcium-dependent learning rate that increases monotonically with calcium levels (Shouval et al., 2002). The parameters θ*_↑_* and θ*_↓_* were determined such that before the copairing period the calcium concentration does not cross either while crossing the potentiation onset θ_↑_ when pairing starts (with θ_↑_ > θ_↓_). The parameters σ, $\gamma_{↑}$ and $\gamma_{↓}$ were chosen to reproduce the experimental results concerning potentiation of CA1 PYR cell EPSC during coactivation of SC and disinhibition/cholinergic inputs (with $\gamma_{↑}$ > $\gamma_{↓}$).

### Stimulation protocol

#### ACh–SC pairing

We constructed a minimal feedforward circuit with an O-cell, a fast-spiking I-cell, and the pyramidal cell s.r. E_D_ connected, as schematically shown in Figure 2*B*, to examine mechanistically how pairing cholinergic activation of the O-cell with glutamatergic activation of the I-cell and E_D_ can potentiate the EPSCs of E_D_. We look at how the EPSC of E_D_, modeled as the sum of the postsynaptic AMPA current (*I*_AMPA_) and NMDA current (*I*_NMDA_), changes when the glutamatergic inputs acting on the I-cell and E_D_ are paired with the cholinergic inputs that act on the presynaptic α7 nAChR of the O-cell during a copairing period of 5 and 8 min, identical to the experimental protocol. The I-cell and E_D_ receive one glutamate pulse per minute before, during, and after the copairing period. During the copairing period, the O-cell gets a pulse of ACh per minute, 100 ms before each glutamate pulse (Δ*t* = 100 ms). Not much is known about the concentration profile of ACh *in vivo*, but it is believed that it can be cleared from the synaptic cleft within milliseconds. After testing different ACh profiles, we decided to model ACh as a square pulse with a duration of 5 ms and concentration of 1 mm, similar to glutamate, although similar results were obtained for a variety of profiles of ACh (Extended Data Fig. 2-3, for more details).

We explore the copairing temporal parameters that regulate plasticity by fixing the frequency of stimulations at 1 pulse/min while varying the copairing period *t*_pair_ (Figs. 2, 3*A*, results). We also study how the frequency of stimulation modulates synaptic plasticity by fixing *t*_pair_ at 4 min while changing the frequency of copaired stimulation (Fig. 3*B*). Finally, we consider different pairing times of ACh and glutamate (Δ*t*; Fig. 3*C*,*D*).

10.1523/ENEURO.0389-21.2022.f3-1 Figure 3-1 Tightly timed pairing of cholinergic to glutamatergic inputs can cancel the I-cell feedforward inhibition. For Δ*t* = –30 ms (Region I), a pulse of glutamate activates the I-cell. When the OLM cell receives a pulse of ACh 30 ms after and releases GABA, the I-cell already emitted two spikes and inhibit E_D_, no plasticity is induced. For Δ*t* =0 ms (Region II), the I-cell and OLM receive a pulse of glutamate and ACh, respectively, simultaneously. Due to its fast dynamics, the I-cell manages to emit one spike before being inhibited by GABA_O_. The I-cell inhibits E_D_ only moderately and depression is induced. For Δ*t* = 100 ms (Region III), OLM receives an ACh pulse at *t* = 0 ms and releases GABA_O_ into the I-cell. When the I-cell receives glutamate 100 ms after, it is hyperpolarized and cannot spike; potentiation is induced. For Δ*t* = 150 ms (Region IV), the hyperpolarization of the I-cell is starting to wear off and the cell manages to emit one spike, sending moderate inhibition to E_D_; depression is induced. For Δ*t* = 300 ms (Region V), the I-cell can emit two spikes when it receives glutamate 300 ms after cholinergic activation; no plasticity is induced. Download Figure 3-1, TIF file.

10.1523/ENEURO.0389-21.2022.f3-2 Figure 3-2 Mean relative pairing timing of single pulses of ACh and glutamate with noisy membrane potential of E_D_ after 10 simulations. Noise was incorporated by adding a stochastic term $\sqrt{dt}\zeta$, where ζ a random Gaussian variable with a mean of μ = 0 and an SD of σ = 0 to the Euler equations describing the *V*_ED_. The mean trace of normalized EPSCs after 10 simulations. When a noisy membrane potential is considered, the transition between the depression and potentiation windows is less sharp (Fig. 3*C*, comparison). Download Figure 3-2, TIF file.

#### Disinhibition–SC pairing

To study the disinhibitory mechanism of plasticity induction, we consider the dendritic compartment E_D_ subjected to glutamate and GABA pulses, as schematically shown in Figure 4*B*. Both GABA and glutamate are modeled as square pulses with a duration of 1 ms and 1 mm of amplitude (Extended Data Fig. 4-2*A*, different durations and amplitudes of glutamate and GABA reproduce the same results), and a frequency of 1 pulse/min, with glutamate preceding GABA by 2 ms.

10.1523/ENEURO.0389-21.2022.f4-1 Figure 4-1 I-cell GABA release evoked can be approximated by a square function. ***A***, Membrane potential of the I-cell when it receives two pulses of glutamate (with an amplitude of 1 mm and a duration of 3 ms) with a frequency of 0.2 ms. ***B***, GABA release from I-cell when it receives the action potentials described in ***A***, calculated using Equation 15. Download Figure 4-1, TIF file.

10.1523/ENEURO.0389-21.2022.f4-2 Figure 4-2 Sets of parameters that qualitatively reproduce Figure 4*D*. ***A***, Numerical simulations of normalized EPSCs of E_D_ for varying the amplitude and duration of the glutamate and GABA pulses. ***B***, Parameters of maximum depression (γ_↓_), maximum potentiation (γ_↑_), synaptic plasticity decay constant (σ), and potentiation threshold (θ_↑_) from the shaded areas qualitatively reproduce Figure 4*D*. The quality of EPSC traces generated with different parameters was evaluated by measuring the relative variations of EPSC amplitude (in non-normalized and non-noisy simulations) from 5 to 30 min after the disinhibition period was over for a 5 and 8 min disinhibition period. Simulations were the variation (percentage of plasticity) was<4% and >22% for the long and short disinhibition periods, respectively, and were considered to conserve the shape of the experimental EPSC trace. This ensures that, for the long disinhibition period, the EPSCs do not decay faster than the experimental EPSCs observed, or slower, for the case of the short period, and therefore have a similar shape. Experimental measures describe the relative increase in EPSC amplitude from the baseline value to 5 min (%_(5-B)_) and 30 min (%_(30-B)_) after the disinhibition period is over (see the Results section for the values of %_(5-B)_ and %_(30-B)_ for 5 and 8 min disinhibition periods). This allows us to derive the relative changes from 5 to 30 min [%_(30-5)_ = (%_(30-B)_ – %_(5-B)_)/(100 + %_(5-B)_) × 100]. By considering the relative changes between 5 and 30 min after the disinhibition period instead of the changes between the baseline and 5 and 30 min, we decrease the number of conditions to evaluate and the computational cost of performing the parameter exploration. The gray and beige areas represent the parameter space where both conditions are met. Note that increasing the synaptic plasticity decay constant σ decreases the robustness of the model to variations of the maximum depression and potentiation, γ_↓_ and γ_↑_ (***B***, beige area). On the other hand, increasing the potentiation threshold θ_↑_ changes the robustness of the model to changes in γ_↑_. As θ_↑_ approaches the depression threshold θ_↓_ or the maximum calcium amplitude Ca_max_, the robustness in γ_↑_ decreases. b1, Gray and beige area: parameter space γ_↓_ – γ_↑_ where the percentage of plasticity is<4% for an 8 min disinhibition period and >22% for a 5 min disinhibition period for σ = 0.004 and σ = 0.005, respectively. b2, Relative variation of EPSC amplitude from 5 to 30 min after disinhibition period (percentage plasticity) for a disinhibition period of 5 min and  σ = 0.005 for different values of γ_↓_ and γ_↑_. b3, Relative variation of EPSC amplitude from 5 to 30 min after disinhibition period (percentage plasticity) for a disinhibition period of 8 min and σ = 0.005 for different values of γ_↓_ and γ_↑_. b4, Relative variation of EPSC amplitude from 5 to 30 min after disinhibition period (percentage plasticity) for a disinhibition period of 5 min and σ = 0.004 for different values of γ_↓_ and γ_↑_. b5, Relative variation of EPSC amplitude from 5 to 30 min after disinhibition period (percentage plasticity) for a disinhibition period of 8 min and  σ = 0.004 for different values of γ_↓_ and γ_↑_. b6, Gray area: parameter region γ_↑_ – θ_↑_ where the percentage plasticity is<4% for an 8 min disinhibition period and >22% for a 5 min disinhibition period for σ = 0.004. b7, Relative variation of EPSC amplitude from 5 to 30 min after the disinhibition period (percentage plasticity) for a disinhibition period of 5 min for different values of γ_↑_ and θ_↑_. b8, Relative variation of EPSC amplitude from 5 to 30 min after the disinhibition period (percentage plasticity) for a disinhibition period of 8 min for different values of γ_↑_ and θ_↑_. b9, Numerical simulations of normalized EPSCs of E_D_ for different points of the parameter space γ_↓_ – γ_↑_ and γ_↑_ – θ_↑_. Download Figure 4-2, TIF file.

10.1523/ENEURO.0389-21.2022.f4-3 Figure 4-3 A square GABA pulse with 1 mm amplitude and 1 ms of duration evokes a GABA_A_ current at E_D_, and decrease NMDA current and depolarization. ***A***, One square pulse of GABA with 1 mm amplitude and 1 ms of duration evokes an inhibitory GABA_A_ current at E_D_ (IGABA_A_). ***B***, When E_D_ receives a GABA square pulse, glutamatergic activation of E_D_ only evokes a depolarization of –63.56 mV (dashed line). ***C***, When E_D_ does not receive GABA inputs, glutamate inputs evoke a depolarization of –58.25 mV (solid line). When E_D_ does not receive GABA inputs, glutamatergic activation evokes a NMDA current of 7.90 pA (solid line). When it receives a GABA square pulse, the evoked NMDA current is 6.75 pA (dashed line). Download Figure 4-3, TIF file.

#### Parameters of the model

We used experimentally determined values or values from previous modeling studies for most of the parameters. Parameters that could not be set experimentally were determined by experimental constraints imposed on the model, namely, the maximal conductances $\bar{g}$*_x_*, and the synaptic plasticity model parameters are indicated with a dash in Table 3.

**Table 3:** Parameter values for calcium dynamics and synaptic plasticity

Parameter	Value	Reference
σ	0.0040 ms^−1^	Materials and Methods
P_1_	1.5e-6	Shouval et al. (2002)
P_2_	P_1_ × 10^−4^	Shouval et al. (2002)
P_3_	13	Shouval et al. (2002)
P_4_	1	Shouval et al. (2002)
θ_↑_	0.34 μm	Materials and Methods
θ_↓_	0.31 μm	Materials and Methods
γ_↑_	0.0687,* 0.0699^†^ nS/ms	Materials and Methods
γ_↓_	0.0375 nS/ms	Materials and Methods
α	0.1	Burnashev et al. (1995)
ξ	0.006,* 0.045^†^ μm/(ms/pA)	Sabatini et al. (2002)
τ_Ca_	12 ms	Sabatini et al. (2002)
ξ′	2.1 × 10^–6^ mm/(ms pA)	Materials and Methods
α′	0.05	Vernino et al. (1994)
τ	10 ms	Materials and Methods
*k*_d_	2 × 10^–4^ mm	Materials and Methods

*Values used to reproduce Figures 2 and 3.

^†^Values used to reproduce the remaining figures.

All the parameter values are defined in Tables 1, 2, and 3. We strived to constrain the parameters to physiological values based on literature, those parameters that we could not directly constrain, were optimized to ranges that ensure that our simulations showed that the measurable variables used are within the physiological range.

We note that different sets of parameters can reproduce our results (Extended Data Fig. 4-2), and that they can be more finely tuned as more experimental data are collected and more constraints are imposed on the model. This also applies for the description of the neurotransmitters ACh, GABA, and glutamate. Despite not having access to data regarding their profile in the synaptic cleft during the experiments performed, we note that the profiles of different neurotransmitters can reproduce our results. In some cases, it may require that free parameters such as ξ and ξ′, the parameters that convert currents into the calcium concentration, are readjusted to keep calcium within the electrophysiological range. In addition, for the particular case of the ACh dynamics, much higher concentrations than the ones considered here may require a more detailed description of the CICR mechanism by, for example, adding a calcium pump to the membrane of the internal stores and the OLM neuron to control the calcium flux into the intracellular medium.

We approximate the solutions of the differential equations with the Euler’s method. We use a step size of 0.02 ms, which is the biggest value for which we have nonerratic solutions. To ensure the stability of our numerical method, we ran a number of pilot simulations with a smaller time step. We found that, for example, a timestep of 0.01 ms did not produce different results, while increasing considerably the time of computation.

### Code accessibility

The code described in the article is freely available at https://github.com/inesCompleto/Hippocampal_Plasticity.

### Data availability

The data that support the findings of this study (Gu et al., 2020) are available from the corresponding author on reasonable request.

## Results

### Coactivation of cholinergic and glutamatergic inputs modifies the SC-CA1 synaptic transmission

First, we set out to study the cholinergic mechanisms by which activation of α7 nAChRs on OLMα2 neurons facilitates the potentiation of SC–CA1 synapses. We designed a biophysical model to reproduce the experimental results reported in the study by Gu et al. (2020; Fig. 2*A*,*C*) using the minimal network scheme presented in Figure 2*B*.

In our model, similar to what was reported in the study by Gu et al. (2020), repeated coactivation of cholinergic and glutamatergic inputs potentiates the SC–CA1 synapse (Fig. 2*D*). The longer the coactivation period, the longer lasting are these changes.

From Figure 2*D*, we see that during the copairing period (from *t* = 10 min to *t* = 18 min), the EPSC is increased. This increase in our model is maintained for an extended period after the copairing period is over (black line), matching experimental results. We also see that GABA release from the I-cells, GABA_I_, decreases significantly (Fig. 2*D*, inset). Before the copairing period, glutamatergic inputs activate the I-cell. This results in the inhibition of the pyramidal cell dendritic compartment E_D_, which shows an SC-evoked depolarization immediately followed by hyperpolarization of its membrane potential. During the copairing period, activation of α7 nAChRs 100 ms before SC stimulation results in a flux of calcium into the OLM cell that will initiate CICR from internal stores exerting a positive feedback. The increase in intracellular calcium concentration induces the release of GABA, as described by Equation 16. GABAergic inputs from the OLM cell disable the SC-evoked activation of the I-cell. As a result, E_D_ does not receive GABAergic inputs (Extended Data Fig. 2-1).

If we reduce the maximal conductance of the α7 nAChR, $\bar{g}$_α7_ from 3 to 1.7 nS as an approximation of the effect of α7 knockout, copairing no longer potentiates the EPSC of E_D_ (Fig. 2*D*, orange line). These observations are in accordance with experimental results showing that this form of EPSC boost was abolished by knockout of the α7 nAChR in OLMα2 interneurons (Fig. 2*C*).

We then examined how the key parameters of the copairing protocol influence the plasticity of the SC–CA1 EPSCs. According to our model, the duration of the copairing period, the relative time between the cholinergic and glutamatergic inputs, as well as their frequency during the copairing period can modulate the efficiency and direction of plasticity. Our simulations show that the longer the copairing period, the longer it takes the EPSCs to return to the baseline value once the copairing period is over (Figs. 2*D*,*F*, 3*A*). We observe a positive relationship between the frequency of the glutamatergic and cholinergic inputs during a fixed period of paring protocol and the potentiation transient duration (Fig. 3*B*). Interestingly, our simulations suggested that while changing the copairing period and the frequency of stimulation modulates the efficiency of the induction of potentiation, it does not change the direction of plasticity. Only when varying the relative time between the ACh and glutamate pulses could we induce a change in the plasticity direction. For single-pulse pairing, potentiation will be induced if the glutamatergic inputs arrive at I and E_D_ within 10.4< Δ*t < *131.1 ms following the ACh pulse. If −19.9< Δ*t < *10.4 ms or 131.1< Δ*t < *177.4 ms, depression is induced (Fig. 3*C*). If we pair doublets of glutamate and ACh with a frequency of 2 Hz instead of single pulses, the potentiation window is 10.9< Δ*t < *149.9 ms, while the depression window is −19.9< Δt < 10.9 ms and 149.9< Δt < 320 ms (Fig. 3*D*). In both cases, the potentiation and depression window are well defined. These results agree with experimental findings by Gu and Yakel (2011) showing that the activation of cholinergic inputs 100 and 10 ms before SC stimulation induced SC to CA1 long-term potentiation and short-term depression, respectively.

In the simulations performed to reproduce Figure 3, *C* and *D*, we do not consider noisy membrane potentials. As a result, we obtain sharp transition between the regions of depression and potentiation—the timing at which GABA_O_ is released from the O-cell finely tunes the number of spikes emitted by the I-cell. As we show in Extended Data Figure 3-2, adding a noisy background induces spontaneous spiking of the O-cells and I-cells, which results in smoother transitions.

### Disinhibition of the CA1 pyramidal cell dendritic compartment enables potentiation of the SC–CA1 synaptic transmission

Our model shows a decrease in GABA release from I-cells during the copairing period (Fig. 2*D*, inset) that results in disinhibition of the E_D_. To study the role of this disinhibition in the potentiation of the SC–CA1 excitatory synapse, we used a model of E_D_ submitted to a pulse of glutamate followed by a pulse of GABA, except during a disinhibition period when it only receives pulses of glutamate (Fig. 4*B*, scheme). This corresponds to experiments where we paired, *in vitro*, the inhibition of Sst interneurons (analogous to the I-cells in the model) with SC stimulation that provides the glutamatergic inputs (Fig. 4*A*).

We would like to note that, according to our model, the rise and decay time of GABA concentration release that results from the spiking of the I-cells is almost instantaneous (Extended Data Fig. 4-1). Therefore, in this section, the GABAergic inputs into E_D_ are modeled as square pulses. For simplicity, both glutamate and GABA release pulses are modeled as square pulses with a duration of 1 ms and 1 mm of amplitude. It is important to note that pulses with amplitudes and durations different from the ones considered here would reproduce the same results, as long as the duration and amplitude of glutamate and GABA match each other (Extended Data Fig. 4-2*A*). E_D_ receives one pulse of glutamate per minute, followed by a pulse of GABA 2 ms after, except during a disinhibition period when it only receives pulses of glutamate. We note that this simulated stimulation and pairing choice directly follows the experimental protocol (see Materials and Methods).

In our model simulations, we observe that before the disinhibition period, there were no changes in the simulated EPSC amplitude of E_D_. During the disinhibition period, the EPSC amplitude increases, and the longer the disinhibition period lasts, the longer these changes last. More specifically, for a disinhibition period of 5 min, the EPSC returns to baseline once the disinhibition period is over. For a longer disinhibition period of 8 min, the EPSC remains potentiated long after the disinhibition period is over (Fig. 4*D*). These results hold for different values of the plasticity parameters (Extended Data Fig. 4-2*B*). After 5 min of E_D_ disinhibition, the EPSC amplitude was increased from 169.40 to 285.34 pA. After 8 min of disinhibition, the EPSC amplitude increased to 361.33 pA. This is in accordance with experimental results, where inhibition of Sst interneurons projecting to CA1 pyramidal cells was paired with SC stimulation for a short and long period (Fig. 4*C*). Inhibition of Sst interneurons via eNpHR resulted in increased SC–CA1 EPSC amplitude not only during the Sst inhibition but also after the end of Sst inhibition. The EPSC enhancement after the Sst inhibition lasted ∼10 min after 5 min of Sst inhibition and >30 min after 8 min of Sst inhibition. After 5 min of Sst inhibition, the EPSC amplitude was significantly increased at 5 min after the end of Sst inhibition (31.8% increase compared with baseline, *p* = 0.0003) but returned to baseline at 30 min after Sst inhibition (2.8% increase compared with baseline, *p* = 0.79). After 8 min of Sst inhibition, the EPSC amplitude was significantly increased at both 5 min after the end of Sst inhibition (37.3% increase compared with baseline, *p* < 0.0001) and 30 min after Sst inhibition (32.5% increase compared with baseline, *p* < 0.0001). Experiments showed that the inhibition of OLMα2 interneurons via eNpHR did not change the amplitude of SC–CA1 EPSC, indicating that the Sst interneurons inducing potentiation do not include OLM (Fig. 4*C*, gray line).

**Figure 5.**
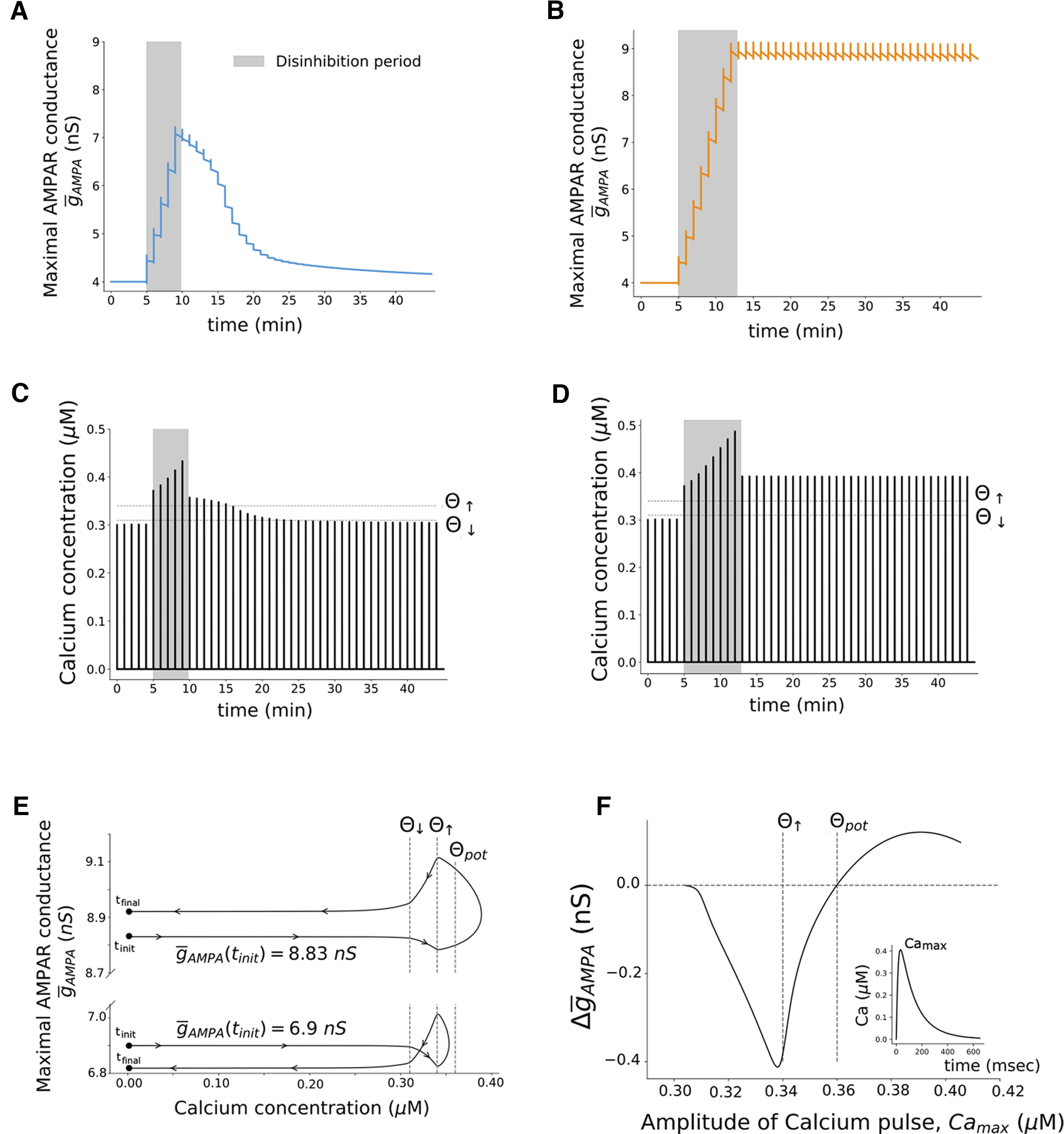
Calcium dynamic is key for the induction of synaptic plasticity. ***A***, Time course of maximal AMPAR conductance, $\bar{g}$_AMPA_, when the dendritic compartment is disinhibited for a short period (from *t* = 5 min to *t* = 10 min). The maximal AMPAR conductance increases from its initial value $\bar{g}$_AMPA_ = 4 nS to $\bar{g}$_AMPA_ = 6.9 nS during the disinhibition period (gray area). ***B***, Time course of $\bar{g}$_AMPA_ when the dendritic compartment is disinhibited for a long period (from *t* = 5 min to *t* = 13 min). It increases from $\bar{g}$_AMPA_ = 4 nS to $\bar{g}$_AMPA_ = 8.83 nS during the disinhibition period. Changes in the AMPAR conductance, $\bar{g}$_AMPA_, are described by Equation 22. ***C***, Time course of intracellular calcium concentration when E_D_ is disinhibited for a short period (from *t* = 5 min to *t* = 10 min), where θ_↓_ is the depression onset, and θ_↑_ is the potentiation onset. ***D***, Time course of intracellular calcium concentration when the dendritic compartment is disinhibited for a long period (from *t* = 5 min to *t* = 13 min). The calcium dynamics is described by Equation 25 (see Materials and Methods). ***E***, Trajectories of the system in the $\bar{g}$_AMPA_–Ca plane when a pulse of glutamate is paired with a pulse of GABA for $\bar{g}$_AMPA_ = 6.9 nS and $\bar{g}$_AMPA_ = 8.83 nS, where θ_pot_ is the potentiation threshold as defined in the Results section. ***F***, Changes in the maximal AMPAR conductance, Δ $\bar{g}$_AMPA_, as a function of the amplitude of intracellular calcium pulse, Ca_max_. Each point of the graph was obtained by submitting E_D_ to a glutamate pulse for different initial values of $\bar{g}$_AMPA_. This induced different depolarization levels and, consequently, different activation levels of NMDARs and calcium pulses of different amplitudes.

AMPARs are known to play an important role in regulating and expressing synaptic plasticity in the hippocampus (Barria et al., 1997). From Figure 5, we see that there is an increase of $\bar{g}$_AMPA_ during the disinhibition period. The longer the disinhibition period, the more significant the increase. For a disinhibition period of 5 min, there is an increase of $\bar{g}$_AMPA_ from 4 to 6.9 nS during disinhibition. Afterward, $\bar{g}$_AMPA_ gradually goes back to its baseline value (Fig. 5*A*). For a disinhibition period of 8 min, $\bar{g}$_AMPA_ increases from 4 to 8.83 nS. When the disinhibition period is over, $\bar{g}$_AMPA_ remains potentiated (Fig. 5*B*). It is important to note that without regular synaptic stimulation, $\bar{g}$_AMPA_ decays back to its resting value after the disinhibition period (i.e., $\bar{g}$_AMPA_ has only one stable fixed point and is not bistable).

In this study, we focused on a calcium-based synaptic plasticity model to describe changes in the excitatory SC–CA1 synapse. To gain a more detailed understanding on how the evolution of the calcium levels relate to the changes in the synaptic strengths, we can examine the calcium dynamics before, during, and after the disinhibition period.

Figure 5, *C* and *D*, shows that the calcium concentration increases significantly during the disinhibition period, crossing the potentiation onset θ_↑_ with a significant margin. Immediately after the end of the disinhibition period, the calcium levels decrease, yet they remain above θ_↑_. We can see a clear difference in calcium dynamics for the short and the long disinhibition periods. In the case of a short disinhibition period, each pairing of GABA and glutamate after the disinhibition period will elicit a calcium pulse with a smaller amplitude than the previous one. Eventually, at *t* = 25 min, the calcium concentration from the pairing is not enough to cross the potentiation onset θ_↑_. By *t* = 30 min, calcium does not cross either the potentiation (θ_↑_) or the depression onset (θ_↓_), having a similar amplitude as before the disinhibition period. In the case of a long disinhibition period, each pairing performed after the disinhibition period evokes a calcium pulse with a constant amplitude. In other words, long disinhibition periods ensure that the consequent pairings yield calcium responses that do not drop below the onset thresholds.

To better visualize the synaptic and calcium dynamics immediately after the disinhibition period in both cases, we plot the trajectory of the system in the Ca- $\bar{g}$_AMPA_ plane. We do so for $\bar{g}$_AMPA_(*t*_init_) = 6.9 nS and for $\bar{g}$_AMPA_(*t*_init_) = 8.83 nS, which are the values of $\bar{g}$_AMPA_ at the end of the disinhibition period for the short and long disinhibition durations (Fig. 5*E*). For $\bar{g}$_AMPA_(*t*_init_) = 6.9 nS, the calcium concentration crosses the potentiation onset θ_↑_ (Ca_max_ = 0.353 μm), but there is a decrease of_<_ $\bar{g}$_AMPA_ from 6.9 to 6.8 nS. For $\bar{g}$_AMPA_(*t*_init_) = 8.83 nS, the calcium concentration crosses θ_↑_ to a larger extent (Ca_max_ = 0.389 μm) and there is an increase of $\bar{g}$_AMPA_ from 8.83 to 8.92 nS. These results suggest that it is necessary but not sufficient for calcium concentration to cross the potentiation onset to induce potentiation. To verify this, we looked at changes in maximal conductance of the postsynaptic AMPAR (Δ $\bar{g}$_AMPA_), as a function of the amplitude of the intracellular calcium (Ca_max_). From Figure 5*F*, we see that as Ca_max_ increases we only start to have potentiation (Δ $\bar{g}$_AMPA_ *>* 0) when Ca_max_ crosses not the potentiation onset θ_↑_, but a higher level, which we term as the potentiation threshold θ_pot_, 0.36 μm.

**Figure 6.**
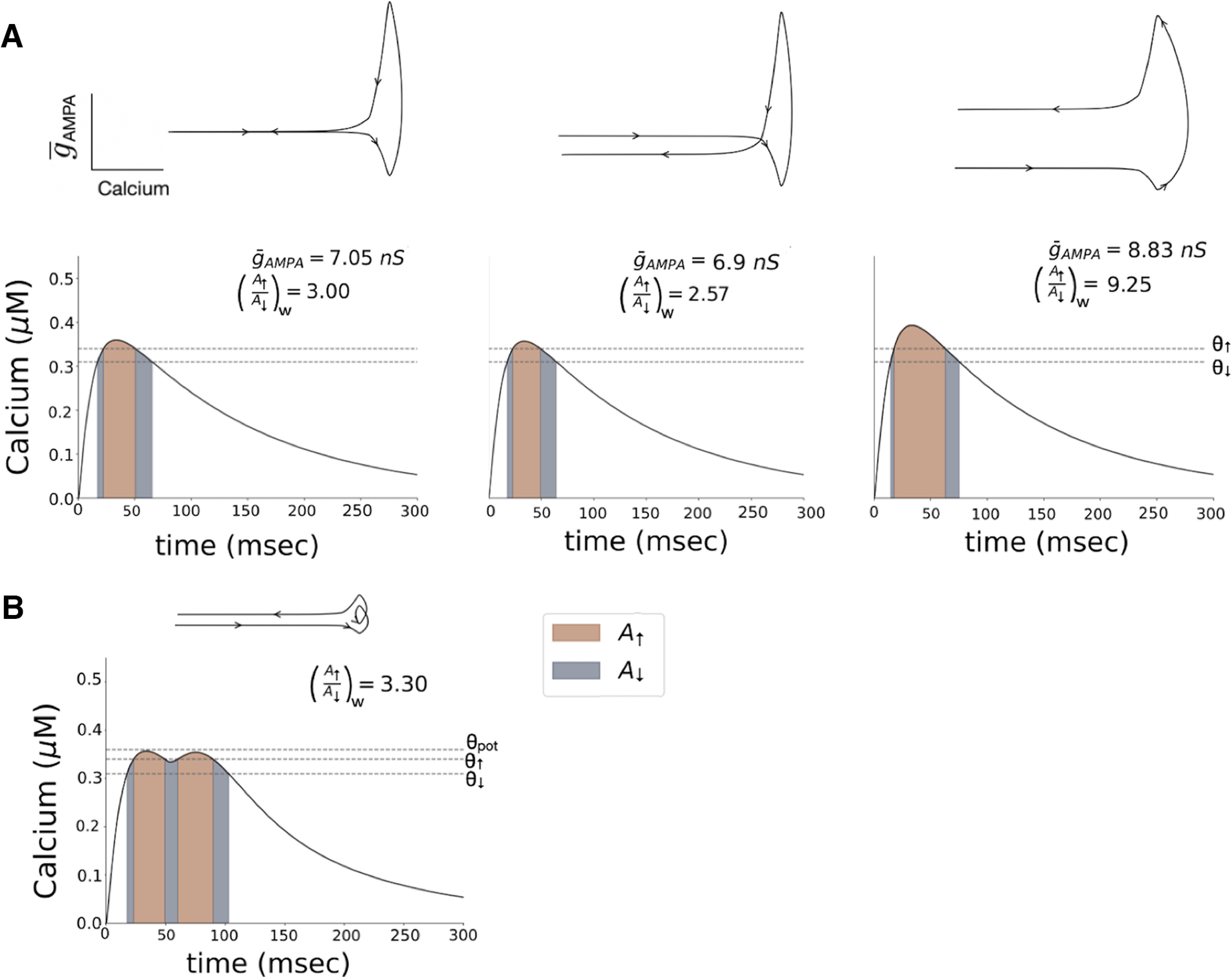
The weighted ratio (A_↑_/A_↓_)_w_ can accurately be used as a predictor of induction of depression or potentiation. The depression and potentiation areas A_↓_ and A_↑_ are as defined in Extended Data Figure 6-1. ***A***, Different values of $\bar{g}$_AMPA_ evoke different levels of depolarization and, consequently, different intracellular calcium concentrations. For a weighted ratio between the calcium area of AMPAR insertion and removal at<3.00, depression is induced. For a value >3.00, potentiation is induced. ***B***, By adding a second source of calcium that becomes activated at *t* = 80 ms, it is possible to have situations where the calcium never crosses the potentiation threshold θ_pot_ but potentiation is induced. The (A_↑_/A_↓_)_w_ accurately identifies these cases as potentiation. In these numerical simulations, E_D_ receives a pulse of glutamate followed by a pulse of GABA 2 ms after, each with an amplitude of 1 mm and a duration of 1 ms.

We do note that the fixed potential threshold θ_pot_ is not an ideal indicator of potentiation, as it may need to be recalculated depending on a specific case of calcium dynamics timescales and/or the induction protocol. As seen in Figure 5*E*, the dynamics of calcium is important in the induction of plasticity. Therefore, changing these by, for example, changing the calcium decay rate, can alter the θ_pot_ by changing the time calcium spends in the depression/potentiation onset region. This kind of analysis can also fail to identify mechanisms of the induction of potentiation. As shown in Figure 6*B*, if we consider a second calcium source that becomes activated at *t* = 80 ms, neither of the two pulses of calcium generated crosses θ_pot_; however, the synapse is potentiated. These examples suggest that it is not the peak calcium concentration that is a key indicator of potentiation, but a measure that is based on the total amount of calcium that exceeds the onset levels. We suggest that a better quantity that can be used more generally as an indicator of plasticity is the ratio between the integral of calcium when its concentration is above the potentiation onset θ_↑_, which we will call the area of AMPAR insertion (Extended Data Fig. 6-1, orange area, Fig. 6, corresponding graphs) and the integral of calcium when its concentration is above the depression onset θ_↓_ and below the potentiation onset θ_↑_, the area of AMPAR removal (Extended Data Fig. 6-1, gray area, Fig. 6, corresponding graphs) weighted by the calcium-dependent learning rate η, which we named (A_↑_/A_↓_)_w_ (for more details, see Materials and Methods). If this ratio is<3.0, depression is induced in our model; if the ratio is >3.0, potentiation is induced.

10.1523/ENEURO.0389-21.2022.f6-1 Figure 6-1 Area of potentiation (orange) and area of depression (gray) considered to calculate the (A_↑_/A_↓_)_w_. For the description of the labels, please refer to Figure 6 in the main text. From *t*_0_ to *t*_1_ and *t*_2_ to *t*_3_, calcium is above θ_↓_ and below θ_↑_. These regions constitute the area of depression A_↓_. From *t*_1_ to *t*_2_, calcium is above θ_↑_. This region constitutes the area of potentiation A_↑_. While the calcium concentration is above the depression onset θ_↓_ (but below the potentiation onset θ_↑_), the maximal conductance of the AMPARs $\bar{g}$_AMPA_ is decreasing. On the other hand, when the calcium concentration is above θ_↑,<_ $\bar{g}$_AMPA_ is increasing. The induction of plasticity at the excitatory synapse depends on the net result of these changes of $\bar{g}$_AMPA_. The more time calcium spends above θ_↑_/θ_↓_, the more likely it is that potentiation/depression is induced at the synapse. Furthermore, the more time calcium spends above θ_↑_/θ_↓_, the bigger the area underneath the calcium curve in this region of insertion/removal of AMPARs. Therefore, the ratio between the area of insertion and the area of removal (A_↑_/A_↓_) can be used as a measure of induction of plasticity (Fig. 6, main text). There is an optimal ratio for which the decrease of $\bar{g}$_AMPA_ resultant from time spent in the removal region and the increase of $\bar{g}$_AMPA_ resultant from time spent in the insertion region will cancel each other and no plasticity is induced. If the ratio A_↑_/A_↓_ is below this value, depression is induced; if the ratio is above this value, potentiation is induced. The ratio A_↑_/A_↓_ is given by $\;\frac{\int_{t1}^{t2}\text{Ca}\;dt}{\int_{t0}^{t1}\text{Ca}\;dt+\;\int_{t2}^{t3}\text{Ca}\;dt}$. Because the decrease and increase of $\bar{g}$_AMPA_ is not the same in the whole removal and insertion region, we need to calculate the calcium integral weighted by the calcium-dependent learning rate η. The (A_↑_/A_↓_)_w_ is then given by $\;\frac{\int_{t1}^{t2}\text{Ca}.\eta \;dt}{\int_{t0}^{t1}\text{Ca}.\eta \;dt+\;\int_{t2}^{t3}\text{Ca}.\eta \;dt}$ . To calculate (A_↑_/A_↓_)_w_, we use the trapezoidal rule to perform numerical integration of the potentiation and depression area. Download Figure 6-1, TIF file.

### GABA amplitude and Glu–GABA pairing timing control membrane potential

**Figure 7.**
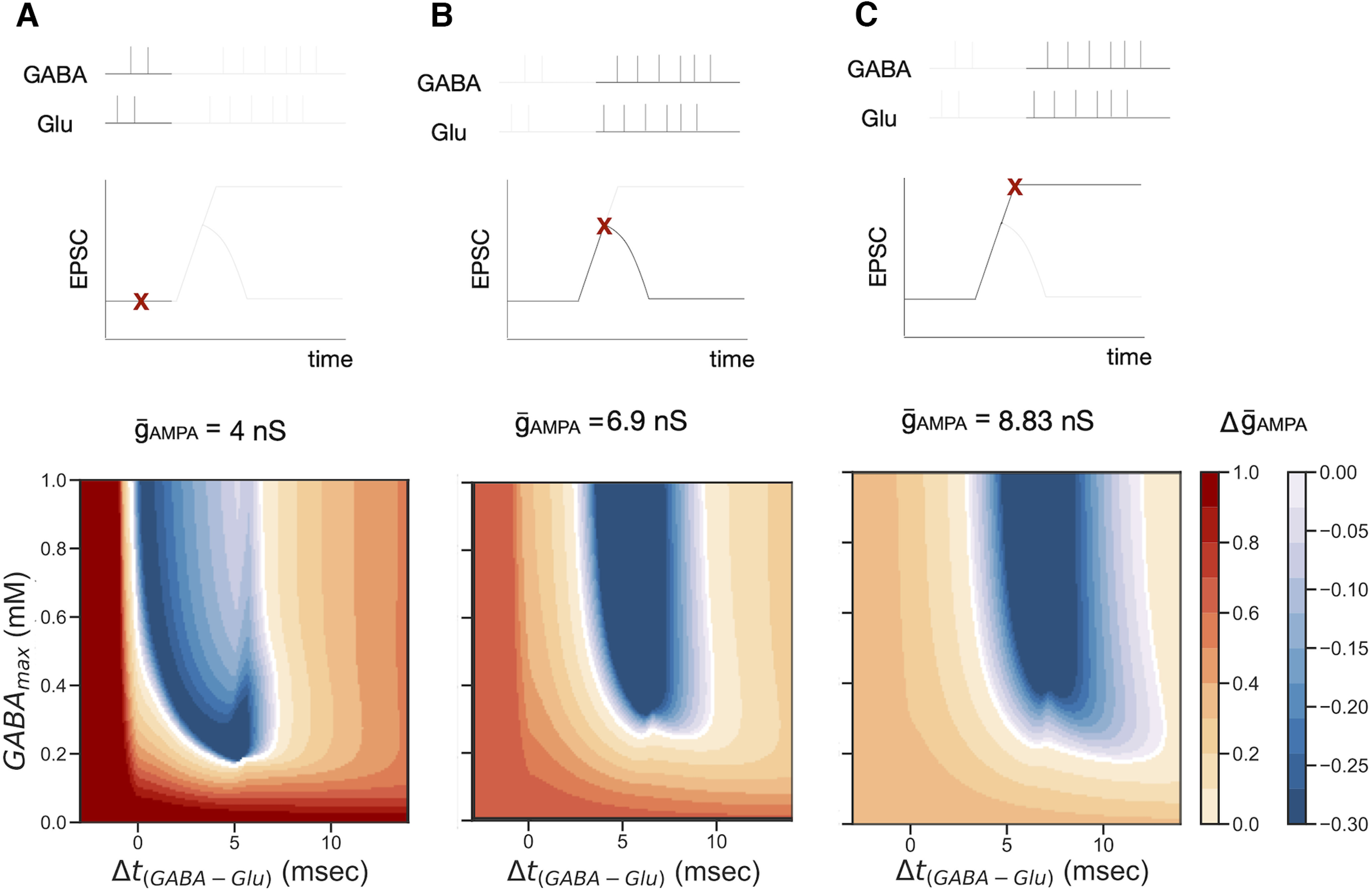
Amplitude of GABA pulse, GABA_max_, and relative time between GABA and glutamate pulses, Δ*t*_(GABA−Glu)_, control the direction and efficiency of the induction of synaptic plasticity. ***A***, Depression and potentiation regions for $\bar{g}$_AMPA_ = 4 nS. This is the maximal conductance value of the AMPAR used in our simulations before the disinhibition period starts. ***B***, Depression and potentiation regions for $\bar{g}$_AMPA_ = 6.9 nS, which represents the state of phosphorylation of the AMPAR at the end of the short disinhibition period. ***C***, Depression and potentiation regions for $\bar{g}$_AMPA_ = 8.83 nS, which is the state of phosphorylation of the AMPAR at the end of the long disinhibition period. For each plot in ***A***, ***B***, and ***C***, we pair one pulse of glutamate (with a concentration of 1 mm and 1 ms of duration) with one pulse of GABA with a duration of 1 ms and varying concentrations and initial time, and measure the resultant change in $\bar{g}$_AMPA_ for each case.

Disinhibition of the pyramidal cell (i.e., reduction of GABAergic inputs), can facilitate the depolarization of the cell, which can control plasticity, as we have shown in the previous section. Therefore, we hypothesize that the amplitude of the GABA pulse, GABA_max_, and the relative time between the glutamate and GABA pulses, Δ*t*_(GABA-Glu)_, can modulate plasticity. To explore this hypothesis, we pair glutamatergic inputs with GABAergic inputs into E_D_. We vary the relative time between the inputs, Δ*t*_(GABA-Glu)_, and the amplitude of the GABAergic inputs, GABA_max_, to measure changes induced in $\bar{g}$_AMPA_. Simulations were repeated for different values of ${\bar{g}}_{AMPA}$ to understand why pulses of glutamate and GABA with the same characteristics (same amplitude and same duration) have different outcomes when administered after the short or long disinhibition periods. Simulations were performed with three initial values of ${\bar{g}}_{AMPA}$: ${\bar{g}}_{AMPA}$= 4 nS, ${\bar{g}}_{AMPA}$= 6.9 nS, and ${\bar{g}}_{AMPA}$= 8.83 nS. We identified well defined regions of potentiation and depression in the Δ*t*_(GABA-Glu)_–GABA_max_ parameter space (Fig. 7). We also saw that the regions change with the value of $\bar{g}$_AMPA_. More specifically, the depression region moves toward the right of the plot as $\bar{g}$_AMPA_ increases. In other words, as $\bar{g}$_AMPA_ increases, the GABAergic inputs need to arrive with a longer delay relative to the glutamatergic inputs to induce depression. It is important to note that the level of potentiation or depression induced also changes as we increase $\bar{g}$_AMPA_. Generally, the magnitude of potentiation decreases, and the magnitude of depression increases. This is because the system saturates as $\bar{g}$_AMPA_ increases (i.e., $\bar{g}$_AMPA_ cannot increase indefinitely). This is a restriction imposed by the model. These results suggest that the same induction protocol may induce either potentiation or depression more or less efficiently, depending on the current phosphorylation state of the AMPA receptors (i.e., $\bar{g}$_AMPA_), and on the decrease of GABA during disinhibition. In other words, the net effect of a pairing protocol is state dependent.

### Model predictions and implications

Results of model simulations and analysis make several testable predictions. First, while experiments so far have not identified precisely the exact type of s.o. interneurons that provide the feedforward inhibition to the CA1 pyramidal cell, our model predicts that it should be an interneuron type with fast dynamics (i.e., with dynamics comparable to the pyramidal cells). More specifically, we expect that EPSC on the hippocampal parvalbumin (PV)-positive interneurons in the stratum radiatum would decrease during cholinergic pairing because of the inhibition provided by the OLM neurons. Consequently, GABA_A_-mediated IPSCs on the proximal dendrites of CA1 pyramidal cells would also decrease.

In this work (both in modeling and experimentally), modulation of the OLM cells is because of cholinergic activation of α7 nAChRs. Our model more specifically suggests that the GABA release by the OLM cells is regulated by activating α7 nACh receptors, without necessarily altering the OLM firing. However, GABA release can also be controlled by the depolarization of the OLM cells and/or by modulation of their spiking activity by somatic nAChRs.

Our model predicts a relationship between the relative timing of the septal and hippocampal stimulus pairing and the synaptic plasticity direction at the SC–PYR synapse. According to our simulations, increasing the frequency of septal and hippocampal paired stimulation can induce plasticity more efficiently (i.e., fewer pairings would be required to induce LTP). At the same time, we predict that changing the relative time between septal and hippocampal activation can induce long-term depression instead of LTP.

Finally, our modeling results suggest that for the plasticity to be induced, the excitatory NMDA and AMPA receptors and the inhibitory GABA_A_ receptors should be located sufficiently proximal to each other in the pyramidal dendritic compartment.

## Discussion

This work set out to explain how nicotinic cholinergic modulation of hippocampal OLM interneurons paired with hippocampal stimulation can potentiate CA1 pyramidal cell EPSC responses. Our modeling results suggest that copairing cholinergic activation of α7 nAChRs on the OLM interneurons results in disinhibition of CA1 pyramidal cells. We also show by mathematical analysis how synaptic plasticity is controlled by the disinhibition of the postsynaptic pyramidal membrane through a disynaptic GABAergic circuit. To our knowledge, this is the first report to reveal how repeated disinhibition can directly induce short-term or long-term potentiation, depending on the duration of the disinhibition period (both experimentally and computationally). It is also the first computational study that explicitly shows how cholinergic action on OLM interneurons can directly induce SC–CA1 plasticity through disinhibition.

OLM cells are a major class of GABAergic interneurons located in the stratum oriens hippocampal layer that inhibit pyramidal cells dendritic compartment located in the stratum lacunusom-moleculare layer, reducing the strength of EC inputs. OLM cells also target bistratified interneurons, expressing PV and somatostatin (Sst), that receive feedforward excitatory inputs from the Schaffer collaterals (Müller and Remy, 2014). Recent findings show that activation of OLM cells can facilitate LTP in the SC–CA1 pathway, likely by inhibiting s.r. interneurons that synapse on the same dendritic compartment as the SC, counteracting SC feedforward inhibition (Leão et al., 2012). We found that repeated pairing of cholinergic inputs with hippocampal stimulation can induce plasticity if the inputs are tightly timed. The time window for potentiation depends significantly on the dynamics of the O-cells and I-cells, and calcium dynamics. This agrees with experimental findings showing that activating cholinergic inputs to the hippocampus can directly induce different forms of synaptic plasticity depending on the input context of the hippocampus, with a timing precision in the millisecond range (McKay et al., 2007; Gu and Yakel, 2011). Our model also shows that the longer the copairing period and the higher the frequency of stimulation during the copairing period, the longer lasting is the potentiation of the synapse.

**Figure 8.**
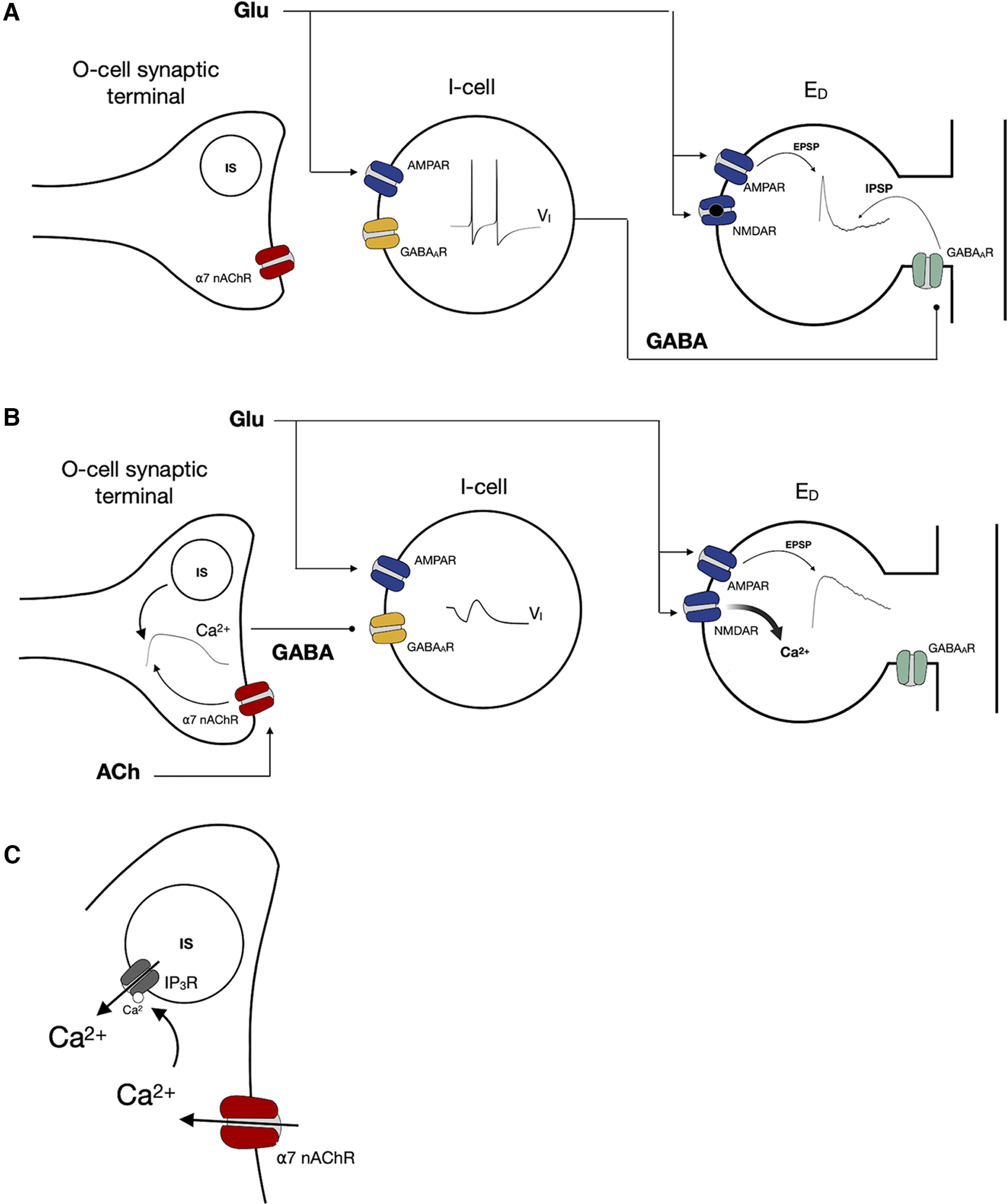
Scheme of the cholinergic and disinhibitory mechanisms that drive SC–CA1 potentiation. ***A***, Glutamatergic activation of I-cells lead to spiking activity and consequent GABA release. Subsequently, glutamate inputs acting on E_D_ evoke an EPSP mediated by AMPAR immediately followed by an IPSP-mediated GABA acting on GABA_A_ receptors. ***B***, Cholinergic activation of α7 nAChR on OLM interneuron initiates a CICR process mediated by calcium internal stores (IS). This result in GABA release that inhibits the I-cell. The dendritic compartment does not receive GABAergic inhibition. The dendritic compartment can depolarize enough—and remain depolarized for long enough—to relieve Mg^2+^ block from NMDA receptors, allowing calcium to permeate through the receptor channel. ***C***, CICR mechanism. The entry of calcium through α7 nAChRs induces calcium release form internal stores by activating IP_3_ receptors.

According to our model, the key mechanism behind paired cholinergic induction of synaptic plasticity is the disinhibition of the pyramidal cell dendritic compartment. Cholinergic activation of the O-cell synapses inhibits the fast-spiking I-cell that projects to the dendritic compartment E_D_. The disinhibition of E_D_ paired with glutamatergic stimulation allows for the depolarization of the pyramidal dendritic compartment. This increases NMDAR activation and intracellular calcium concentration sufficient to upregulate postsynaptic AMPAR permeability and potentiate the excitatory synapse. Our model puts together all the elements to give the following sequence of events: SC stimulation results in the activation of CA1 fast-spiking interneurons, I, and the subsequent release of GABA. At the same time, it evokes an EPSP mediated by AMPAR on the CA1 pyramidal cell dendritic compartment, E_D_. Since I and E_D_ have comparable dynamics, the EPSP is closely followed by a GABA_A_-mediated IPSP. Because of slow kinetics and voltage dependence, at that time, the NMDAR receptors are not in the open state and there is no significant influx of calcium. When the SC inputs are tightly timed with cholinergic inputs acting on OLM interneurons, GABA release from I-cells is suppressed. The pyramidal cell membrane at (or sufficiently near to) the glutamatergic synapse can depolarize enough to relieve the Mg^2+^ block from the NMDA receptors, allowing calcium to permeate through the receptor channel (Fig. 8). Therefore, every time the pyramidal cell receives a glutamate pulse during the disinhibition period, the intracellular calcium concentration crosses the potentiation outset θ_↑_, and $\bar{g}$_AMPA_ increases.

Downregulation of the GABAergic signaling during disinhibition leads to increased NMDAR activation. We see that when we reduced GABA concentration, glutamatergic activation of E_D_ results in postsynaptic NMDA currents with 7.90 pA of amplitude—with depolarization of −58.25 mV, as opposed to the 6.75 pA that results from the pairing of glutamate and GABA inputs—with depolarization of −63.56 mV (Extended Data Fig. 4-3). Because of the high calcium permeability of receptor, there is an elevation in intracellular calcium concentration large enough to initiate molecular mechanisms that result in the insertion/phosphorylation of the AMPAR. In our model, this translates into an increase in the AMPAR maximal conductance $\bar{g}$_AMPA_. Moderate calcium concentrations, on the other hand, result in the removal of AMPARs. Because changes in calcium concentration are not instantaneous, the potentiation/depression of the synapse results from a balance between the insertion/removal of AMPARs during the period in which Ca concentration is above the potentiation/depression threshold. During disinhibition, this balance is positive and there is a total increase in $\bar{g}$_AMPA_.

The more times we pair disinhibition with SC stimulation (i.e., the longer the disinhibition period), the higher the value of $\bar{g}$_AMPA_ by the end of the disinhibition period. After the disinhibition period, if the increase of $\bar{g}$_AMPA_ was large enough, the calcium resultant from glutamatergic and GABAergic stimulation is such that there is a balance between potentiation and depression close to zero. That is, $\bar{g}$_AMPA_ stabilizes by oscillating around the value of $\bar{g}$_AMPA_ at the end of the disinhibition period (8.83 nS). Therefore, the synapse remains potentiated long after the disinhibition period is over. If there is no stimulation after the disinhibition period, $\bar{g}$_AMPA_ slowly decays to its initial value (i.e., its value before the disinhibition period). Supposing that the increase of the AMPAR permeability is high enough, the potentiation of the excitatory synapse is maintained when the disinhibition period is over through repeated stimulation of the SC that keeps a balance between the downregulation and upregulation of the AMPARs. This is in accordance with experimental results that show repeated pairing of the inhibition of Sst interneurons (that were not OLM) that target the CA1 pyramidal cell with SC stimulation can induce plasticity.

We asked how the results of our simulations depend on the parameters chosen. We found that our model remains robust to changes of parameters as long as we maintain the same ratio of insertion/removal of AMPARs. Thus, for example, for different values of the $\gamma_{↑}$, there is (at least) a pair of ${\text{γ}}_{↓}$ for which our results remain the same (Extended Data Fig. 4-2*B*).

In our modeling study, we strived to ensure that parameters for which physiological ranges can be identified agree with these ranges. At the same time, there were a number of them that could not be constrained directly, and we chose to optimize them to obtain model responses that qualitatively agreed with our data. For example, the calcium amplitude in our model is of the same order of magnitude as measured in the dendritic spines in the studies by Sabatini et al. (2002) and Rubin et al. (2005). While we do see that the optimized Ca*_p_* parameter is below the calcium resting value, we consider that calcium concentration decays to zero, similar to what is done in the studies by Graupner and Brunel (2005, 2012), Higgins et al. (2014), Rubin et al. (2005), and Shouval et al. (2002). Despite not being an exhaustively detailed description of what happens in the dendritic spine, changing the resting value of the calcium does not alter our results as long as it is below the depression threshold. Concerning the K_(Ca)_*_p_* parameter, it determines the steepness of the GABA_O_(Ca) function. The vesicular release of neurotransmitter has a steep dependence on the intracellular calcium concentration Schneggenburger and Forsythe (2006). Thus, we believe it to be appropriate to consider a steep relationship between the intracellular calcium and the concentration of GABA available for binding, and that these two parameters are examples for functionally optimized values.

It is worth noting that the parity of the synaptic plasticity induced depends on the value of maximal conductance of the postsynaptic AMPAR, $\bar{g}$_AMPA_, as shown in Figure 7. Therefore, our model indicates that future changes in synaptic strength depend on previous plasticity events and how these changed $\bar{g}$_AMPA_. This explains why, after the disinhibition period, glutamate–GABA pulse pairs with the same characteristics will induce different results when the disinhibition period was short or long.

Earlier studies pointed out that reduced inhibition (disinhibition) can facilitate LTP induction under various conditions (Wigström and Gustafsson, 1985; Ormond and Woodin, 2009; Yang et al., 2016). Our results show that repeated temporally precise concurrent disinhibition can directly induce SC to CA1 LTP, providing a novel mechanism for inhibitory interneurons to modify glutamatergic synaptic plasticity directly. This expands the original spike timing-dependent plasticity that concerns the concurrent activation of two excitatory pathways to include the interneuron network. Furthermore, our modeling work implies that GABAergic neurotransmission should control the local pyramidal voltage in the vicinity of the glutamatergic synapses, thereby the inhibitory synapses critically modulate excitatory transmission and the induction of plasticity at excitatory synapses. This points toward the importance of dendritic GABA and glutamate colocation in shaping local plasticity rules. Our work also suggests a cholinergic mechanism for controlling GABA release at the pyramidal dendrites and the subsequent potentiation of excitatory synapses, unraveling the intricate interplay of the hierarchal inhibitory circuitry and cholinergic neuromodulation as a mechanism for hippocampal plasticity.

Previous work by Gu et al. (2017) showed that copaired activation of the cholinergic input pathway from the septum to the hippocampus with stimulation of the Schaffer collateral pathway could readily induce theta oscillations in a coculture septal–hippocampal–entorhinal preparation. Moreover, after performing copaired activation several times, not only was the SC–PYR synapse potentiated, but it became easier to evoke the theta rhythm in the preparation (one pulse stimulus of the SC is sufficient to generate theta oscillations in the circuit with the same characteristics as before; Gu and Yakel, 2017; Gu et al., 2017). Therefore, induction of hippocampal plasticity, particularly potentiation of the CA1 EPSPs, appears to facilitate the generation of the theta rhythm. Moreover, previous studies directly linked OLMα2 interneurons to theta oscillations (Rotstein, et al., 2005; Mikulovic, et al., 2018) and suggest that OLM cells can regulate the robustness of the hippocampal theta rhythm (Chatzikalymniou and Skinner, 2018). Thus, we may speculate that the action of ACh on the α7 nAChRs at the OLMα2 neurons potentiates the SC–CA1 synapses to close the critical link in the synaptic chain of events, enabling recurrent reverberation excitation in the hippocampal–entorhinal theta-generating circuit. Understanding the mechanisms underlying the induction of hippocampal plasticity by this copairing mechanism will allow future studies of how changes on the synaptic level can propagate to the network level and change the mechanisms of theta generation.

Our results are also relevant to understanding the neural circuit origins of pathologic conditions and uncovering potential targets for therapeutic intervention in disorders linked to memory deficits. For example, the hippocampus is one of the earliest brain structures to develop neurodegenerative changes in AD (Arriagada et al., 1992). Furthermore, numerous studies suggest that cognitive deficits in AD, such as memory impairment, are caused in part by the dysfunction of cholinergic action on hippocampal GABAergic interneurons (Schmid, et al., 2016; Haam and Yakel, 2017). Here, we have shown that a decrease in the conductance of cholinergic α7 nAChRs on OLM interneurons caused the impairment of induction of hippocampal synaptic plasticity.

### Model caveats

As with any modeling studies we had to compromise between a realistic description of the neural networks and the simplicity of the model that allows for computational analysis. While some of the aspects could be performed using simplified integrate-and-fire neuron models, we felt that multiple aspects focused on biophysical mechanisms (e.g., the ability of the cholinergic activation of OLM cells to suppress spiking of fast-spiking interneurons in a tightly timed manner). The simplified one-compartment biophysical model used in this study allows us to analyze how detailed biophysical mechanisms, such as CICR and the dynamics of neurotransmitter release, control cholinergic induction of plasticity, while maintaining the simplicity and flexibility necessary to carry out computational analysis and study similar mechanisms in other neural networks.

The assumptions and ad hoc simplifications made in this study introduce some limitations in the model. Namely, the description of the ACh pulse that, because of a lack of knowledge of the neurotransmitter profile in the synaptic cleft, is described as a square with 1 mm magnitude and a duration of 5 ms. We have shown that our results still hold up when considering different cholinergic profiles (Extended Data Fig. 2-3); however, a magnitude or duration considerably higher (lower) than what is considered here can lead to calcium concentrations that are too high (low). This can lead to nonphysiological calcium concentrations and, consequently, unrealistic GABA profile. In that case, one would have to consider a more detailed description of the CICR mechanism with calcium pumps on the internal stores and the membrane of the neuron. The same adjustment would be necessary if the resting calcium concentrations in the internal stores and intracellular medium induce a greater calcium flux.

## Synthesis

Reviewing Editor: Arvind Kumar, KTH Royal Institute of Technology Decisions are customarily a result of the Reviewing Editor and the peer reviewers coming together and discussing their recommendations until a consensus is reached. When revisions are invited, a fact-based synthesis statement explaining their decision and outlining what is needed to prepare a revision will be listed below. The following reviewer(s) agreed to reveal their identity: NONE.

Synthesis:

In this paper authors have combined in vitro experiments with computational modelling and describe how EI balance in CA1 can be modulated by cholinergic input to the OLM neurons. Activation of nicotinic receptors on OLM neurons causes GABA release which inhibits the FSI and thereby modulates the EI balance. Disinhibition of pyramidal cells in turn affects calcium levels and the LTP at the SC-CA1 synapses. Overall, both the reviewers thought that the work addresses an interesting question and the detailed analysis of results could be relevant to future experimental work on SC-CA1 plasticity and role of neuromodulation in the hippocampus.

However, the reviewers have raised many substantial concerns such as :

At a number of places several crucial details are missing (see reviewers’ comments).

The model design choices and the choice of parameters should be properly justified and discussed. For example, the direct release of GABA by nicotinic induction of calcium influx is an unusual feature of the model and should be explained in more detail. Or why, such a complex model is necessary when entire analysis could have been done with simple alpha-function synapses and integrate-fire neurons. Many model parameters are missing and in some instances, text in the methods section is not consistent with that in the results section.

In the manuscript it is often not clear which results come from in vitro experiments and which from the computer model.

A number of citations are missed. Please refer to the reviewers’ comments for details.

Overall the paper is rather difficult to read and need proof-reading

Besides, the reviewers have made several important suggestions to improve the manuscript.

It would be good to put in blockers to confirm the affected pathways; and to vary the ACh and electrical stimulus parameters. As it stands this is a single observation which does not provide strong constraints for the modeling.

Authors should perform the sensitivity analyses on the model results.

Separately show the short term acute increase in EPSCs (perhaps with a shorter simulated stimulation protocol) and the long term potentiation of EPSCs.

The time difference cut-off points in Figure 3C and D are very abrupt and seem unrealistic, probably due to lack of noise in the calcium concentrations. Authors should discuss this and be clear about whether they would predict smoother transitions in experimental data if this were tested. [see Point 6 in the comments by reviewer 2]

The authors report a sharp change in system properties when disinhibition duration is changed from 5 min to 8 min. Please explain and explore more completely. Is this a phase transition or an analogue effect?

A more detailed schematic figure to better illustrate the complexity of the dynamics of calcium and of synaptic release.

Please show all the results. ‘Simulation not shown’ is not consistent with journal policies.

Please provide the simulation code.

Given this, the manuscript needs a substantial revision. The detailed comments of the reviewers are appended. Please revise the manuscript and provide a detailed point-by-point reply.

Comments from the reviews

Reviewer 1

This study takes on the interesting and important question of how EI balance in the hippocampus CA1 can be modulated by cholinerging input to OLM interneurons. This modulation can affect plasticity on the SC-CA1 synapses. The paper sets out to combine in-vitro experiments and models, which is a desirable thing, but the emphasis is clearly on the modeling.

General points.

1. The paper is quite difficult to read. For example, the first 10 or so lines in the results section are actually background that should be covered in the introduction. The next 20 or more lines are model choices, most of which

belong in the methods. The results sections seem to make a lot of different points each. Summaries and section breaks might help. Also see point 9, about separation between experiments and models.

2. The authors have picked a potentially interesting combination of detailed channel and calcium dynamics, with very simplified neuronal and connectivity models. I was not able to find a clear motivation for this particular model configuration. I felt that the entire analysis could have been done with simple alpha-function synapses and integrate-fire neurons. Can the authors justify their model design choices?

3. Figure 4 C seems to be the crucial experiment to infer a sharp difference between EPSC responses for different durations of inhibition. This could greatly benefit from more follow up and characterization, as well as a clear statement of number of observations (which might be 5 and 8 respectively, but it isn’t clear). Among other things, it would be good to put in

blockers to confirm the affected pathways; and to vary the ACh and electrical stimulus parameters. As it stands this is a single observation which does not provide strong constraints for the modeling.

4. Have the authors performed any parameter sensitivity analyses? Most of their data sources are actually other simulations, not from the primary experimental data. It is difficult to understand whether the results of the study are

robust or fragile.

5. The authors do not seem to have provided source code nor a link to a GitHub repository. I am therefore unable to assess the software. The provision of code for modeling studies is essential.

Specific points.

6. Line 187: The authors “assume that the ⍺alpha-7 nAChR current is not strong enough to elicit action potentials directly” Since they have the cell model, why not calculate this? The introduction of equation 16 seems unnecessary and is fraught with assumptions.

7. Cap of 40 nM (4e-5 mM, line 193) is well below resting calcium, and K(Ca)p of 1nM (line 192) seems extraordinarily steep. Can the authors give a physiological justification for the use of such numbers?

8. There is a rather complex internal Ca model in alpha-7 cells, involving CICR. Again, I don’t see why this level of detail contributes to the understanding. If one has to put in complex calcium dynamics it should be for the synaptic

plasticity model.

9. Can the authors make a clear distinction between modeling and experimental results? Is Figure 3 modeling or experimental? From the text it seems to be modeling, but the figure legend does not make this clear. The text is also confusing in this regard for Figure 4, though the legend is clearer.

10. Can the authors provide a more detailed schematic of their model? In particular, there is considerable complexity in the dynamics of calcium and of synaptic release, which is invisible in the schematics provided.

11. Line 329: “GABAergic inputs to ED are modeled as a square pulse” If you have such a complex set of cells in the model, why is GABA being treated in this oversimplified way? There is an equation 16 for synaptic release, that one could apply to this synapse, or one could incorporate the GABA term with the GABAR dynamics in the usual alpha-function formulation.

12. Around line 331 the authors say that other pulse shapes would give the same results, but they say “simulations not shown”. The simulations should be shown.

13. The authors report a sharp change in system properties when disinhibition duration is changed from 5 min to 8 min. Please explain and explore more completely. Is this a phase transition or an analogue effect?

14. On line 362 the authors say that gAMPA has only one stable fixed point without synaptic simulation. However, it isn’t stated whether it would become bistable under conditions of regular synaptic stimulation.

15. The authors chose to use the Euler method, which is not numerically ideal. How did they ensure accuracy? What timestep did they use? What validation did they carry out to ensure that it worked as expected?

Reviewer 2:

This paper presents a biophysical model describing how direct activation of cholinergic terminals can activate nicotinic receptors in OLM cells that cause GABA release that inhibits fast-spiking interneurons, thereby allowing increased depolarization and higher calcium levels in pyramidal cell dendritic compartments that trigger long-term potentiation. Overall, the detailed analysis of results could be relevant to future experimental work on this physiological phenomenon. However, there are a number of aspects that need to be presented much more clearly.

MAJOR COMMENTS:

1. Figure 1 shows the nAChR receptors on the presynaptic terminals of OLM cells, but this localisation of receptors is not discussed much in the text. They should mention this in the text and they must provide citations for this anatomical location of nAChR on OLM cells.

2. The direct release of GABA by nicotinic induction of calcium influx is an unusual feature of the model. They need to provide more detail and description about the experimental data supporting this property of the model (not just a citation) and must describe why they do not think the nicotinic receptors are modulating spiking induced release of GABA.

3. They need to be clearer in the methods about the timing and duration of cholinergic stimulation. Currently the description in the methods seems to not match the description in the results. In the methods, they should mention both the 5 min and the 8 min stimulation period and the intervals between stimulation (once per min). (The duration looks shorter than 5 or 8 mins in Figure 2C. Also, the comments about one pulse per minute seem inconsistent with the higher rates (e.g. 1/3) described in Figure legend 3B. Basically, they need to give a clearer overview of the stimulation in the Methods and at the start of the Results section (which could use elements of the description from Figure legend 3B.

4. They need to be much clearer in the figures about what results are from the model simulations and what results are from the experimental work. I suggest adding clear labels of “MODEL” and “DATA” where relevant.

5. In the figures, they should separately show the short term acute increase in EPSCs (perhaps with a shorter simulated stimulation protocol) and the long term potentiation of EPSCs. They primarily show the long term potentiation, but don’t seem to show examples of the acute increase without long-term potentiation.

6. The time difference cut-off points in Figure 3C and D are very abrupt and seem unrealistic, probably due to lack of noise in the calcium concentrations. They should discuss this and be clear about whether they would predict smoother transitions in experimental data if this were tested. They incorporate noise in other simulations, but they don’t seem to be including it in these plots? I liked supplementary figure 7 as this explains how the generation of spiking induces the depression dynamics at low and high time differences. However, if multiple examples similar to this figure were combined with different levels of noise, I would think that noise would change the position of the locus of this temporal transition into spike induction and thereby give less sharp transitions.

7. The authors leave out a lot of relevant citations. They ought to cite earlier work on cholinergic modulation of interneurons, and cholinergic modulation of LTP. For the cholinergic modulation of interneurons, they should cite work by Pitler and Alger, 1992 J. Physiol. 450: 127; Behrends and ten Bruggencate, 1993; J. Neurophysiol. 69:626; Patil, J. Neurophysiol 1999, 81: 2103-2118; Bell et al. 2011 Neuropharmacol 61:1379; Bell ,Bell, McQuiston 2015, J. Physiol. 593: 197.

8. The description is ungrammatical in many places. They should have someone with strong English writing skills proofread the paper.

9. They should be clear about whether the experiments described here were entirely reported in previous papers, or if some component is novel. I believe the experimental data is all previously presented, so this should be stated more clearly in the methods and results sections.

SPECIFIC COMMENTS

There are many things that they should cite in this paper.

Page 2 - There are a number of previous studies that they should cite. Some examples are provided in the major comments. They should also cite work from groups like the McQuiston and Alkondon groups for effects of nicotinic receptors on interneurons.

Page 2 - Should cite Hannah Monyer lab paper in entorhinal cortex showing inhibition induced by cholinergic stimulation. Desikan, Koser, Neitz, Monyer PNAS 115: E2644.

Page 2 - Increased SC to CA1 EPSCs - They should provide more details from Gu et al. 2020. They should mention if this was tested by recording of intracellular or field potentials?? They should also specify clearly if this is a short term acute effect or is it a long term potentiation effect. The later mention of “potentiation” suggests it is long term, but they need to be clear about this and should always specify “long term potentiation” or “short term potentiation”.

Page 2 - “how this facilitates the potentiation” - This sounds like they are always referring to long term potentiation, but the short term disinhibition could cause acute increases in EPSCs without causing long term potentiation. They need to use clear language.

Page 3 - “reduced inhibitory inputs can directly induce the plasticity” - this is incorrect - they do NOT show that reduced inhibition directly causes plasticity? What they show is that the effect is indirect by allowing increased calcium influx through NMDA channels, thus they should say “inhibitory inputs can indirectly enhance the plasticity"

Page 4 - “similar as described in (Gu and Yakel, 2011, 2017) = are they presenting new data? They need to be clear about whether this is new or a review of previous data -

Page 4 - Before pairing by student t-test - this is vague - They should say: “testing of statistical significance by students t-test”?

Page 4- They should itemize what time periods are used for generating the mean and variance in the baseline and experimental effect used in the t-test.

Page 6- an action potential in CA1 pyramidal cells is insufficient to make the OLM cell membrane potential cross.... But what about multiple CA1 cells?

Line 136 - what simulation package was used? Was it Neuron? It is unlikely that they generated this model from scratch. They need to specify the simulation package or describe how it was programmed from scratch.

Lin 172 - what is the value of tau r alpha 7?? They should specify the table where this is presented later.

Lne 183 - as a function of presynaptic voltage - does this mean the shape depends primarily on the action potential? What about all the synaptic release mechanisms and diffusion?

Line 189 - calcium concentration sufficient to activate vesicular release (Griguoli and Cherubini, 2012) - they should provide more description of the experimental results supporting this model as this is unusual - is there really enough calcium through nicotinic receptors - are they on the presynaptic terminals of the GABA cells??

Line 210 - tau alpha 7 - They give the source of the value, but when will they actually give the value itself - they should give the table.

Lone 222 - same order of magnitude as observed experimentally - they should show this

Lin 252 - tau Ca - should provide the numerical value

Line 267 - previously - should give citation after first sentence

Line 217 - OLMalpha2 interneuronal ...- interneuronal what? A word seems to be missing.

Line 110 says ACh was activated by 10 pulses at 10 Hz -

Line 268 - “8 times” and line “8 minutes” - the description in the methods seems incomplete or inconsistent relative to the discussion in the Results - 8 times is not equivalent to 8 minutes and neither matches 10 pulses as described in the methods. As noted in major comments, the methods should describe the details as presented in the figure.

Line 270 - The induction of potentiation - is this long term or short term potentiation - the term potentiation is ambiguous and could mean short term acute increase or a longer term sustained increase

Line 280 - on the postsynaptic nAChR - again are these on the presynaptic terminals of the OLM cells - otherwise it’s hard to see how they could cause calcium influx that would cause direct release of GABA

Line 285 - duration of 5 msec - this seems inconsistent with the amperometry studies of Parikh et al. 2007, Neuron 56: 141-154. showing durations of ACh increases around 2 seconds long.

They should discuss this explicitly as their duration seems too short.

Line 291 - results in ED inhibition - should be clearer by saying inhibition of the pyramidal cell dendritic compartment -

Line 302 - co-paring

Line 312 and 316 - They need to be clear about the direction of the subtraction yielding delta t- otherwise the results about 100 msec versus 10 msec can be confusing - make clear that 10msec=

Line 329 - sup figure 6 - why is this the first one shown? Ordering of supplementary figures seems odd

Line 370 - smaller amplitude - is this due to changes in release from internal stores or changes in NMDA channel conductance?

Line 399 - ‘Given that this..., indicates...- this is not grammatical

Page 18 - They should insert paragraphs breaks somewhere on this page

Line 428 - ‘during cholinergic pairing = not only would EPSCs decrease, but they seem to predict a direct IPSC in fast spiking interneurons caused by nicotinic activation after cholinergic simulation

Line 448 - “first report” - should avoid trying to claim primacy. There are lots of earlier studies of LTP showing that blockade of inhibition enhances LTP.

Line 450 - “first computational study” - Other studies have analyzed effects of cholinergic modulation though not these specific timing effects of nicotinic receptors (e.g. Hasselmo and Barkai, 1995; Barkai et al. 1994, J. Neurophysiol.).

Line 454 - expressing SST - the OLM cells also express SST in addition to the bistratified cells - so it is not clear why they can separately manipulate SST cells

Line 460 - “activating cholinergic inputs...synaptic plasticity” - they should cite the many earlier studies showing cholinergic enhancement of LTP in hippocampus and other structures - For effects on LTP, they should cite Burgard and Sarvey 1990 Neurosci. Lett 116: 34; Hasselmo, Barkai 1995, J. Neurophysiol. 15: 6592; Patil, J. Neurophysiol. 1998, 80:2467 showed timing dependent effects.

Line 499 - “SST interneurons...that were not OLM” - how was this determined? This is confusing.

Line 526 - inducing theta oscillations - the influence of septum on theta oscillations has also been shown by Toth, Freund and Miles J. Physiol. 1997 500: 463 and by the Sylvain Williams group (Sotty et al. 2003 J Physiol 551:927.)- these should be cited.

It’s good that they cite the work on the role of OLM cells in theta oscillations (Mikulovic et al., 2018, 9: 3638), but there is other relevant work. They should also cite here the work by Rotstein et al. 2005 on the role of OLM cells in theta rhythm here which they cite in the methods section but should be cited here.

Line 535 - “change theta generation’s mechanisms” - this is an awkward phrase. It is also very implausible as the long term potentiation is slow, whereas the induction of theta can be very rapid, so it is likely that it is due to modulation of the GABAergic input coupled with direct cholinergic depolarization of neurons. The text should be altered to reduce the emphasis on long-term potentiation and mention the mechanisms of rapid modulation of theta (e.g. Buzsaki, 2002; Goutagny, Jackson, Williams , 2009 Nat. Neuro 12: 1491.

Line 540 - “cholinergic dysfunction action” - this is ungrammatical

Figures 2 and 3 - They need to be very clear about what results in these figures are simulations from the model and what sections are showing experimental data (adding labels of “data” and “model” would help).

Page 36 - “sst and OLMalpha2” - I always understood OLM cells to be SST positive. How are they able to differentiate OLM versus SST cells? This is very confusing.

Page 39 - “by adding a second source” -I don’t recall this being mentioned in the methos and results - this should be stated more clearly in the results at least.

Page 43 - Do they have data on inhibition in interneurons induced by ACh activation? They should definitely cite the paper from the Monyer lab (Desikan et al. citation provided above) in which activation of cholinergic terminals was shown to cause brief inhibition in pyramidal cells (and discuss why they did not include this in their model and instead put the inhibition into fast spiking interneurons).

Supplemental figure 4 - “two spikes” - they should note that this is shown with the dashed line and then note what the solid line represents.

Page 49 - “membrane potential of the I cell” - this is a very non-physiological time course for membrane potential. Usually stimulation of axonal input causes a dual-exponential time course, not a square wave (even if the presynaptic release is modeled as a square wave).

## Author Response

Dear Editor,

Thank you for taking the time to assess our manuscript and for the insightful suggestions. We would also like to thank the reviewers for their detailed comments.

After carefully reading the reviews, we have addressed almost all of the comments and it is our belief that the revised manuscript is substantially improved (see revised manuscript attached where modifications are highlighted). You can find bellow a detailed reply to each of the points raise by the reviewers.

We note that in order to align the structure of the manuscript with the formal requirements of eNeuro publications, the Supplementary Figures and Information of the original manuscript are submitted as Extended Data. We have also revised the title to Temporally recurrent cholinergic inputs induce local hippocampal plasticity through feedforward disinhibition (in the original manuscript: Recurrent cholinergic inputs induce local hippocampal plasticity through feedforward disinhibition) as we feel the term recurrent may be wrongfully associated to recurrent circuit connections.

Reviewer 1

General points:

1. The paper is quite difficult to read. For example, the first 10 or so lines in the results section are actually background that should be covered in the introduction. The next 20 or more lines are model choices, most of which belong in the methods. The results sections seem to make a lot of different points each. Summaries and section breaks might help. Also see point 9, about separation between experiments and models.

- We took the reviewer’s advice to move the first part of the results section to the introduction (page 2, lines 47-49) and methods (page 15, lines 291-299). We have added clear labels on the figures and respective captions indicating if the nature of the results (experimental or model). We have also made several changes in the structure of the manuscript to make it easier to follow, which include a new section in the Methods where we detail the stimulation protocols used to address questions (pages 15-16, lines 290-313), an account of the model’s ad hoc assumptions and limitations as well as their impact in our work (page 16, lines 315-334 and page 27, lines 622-638).

2. The authors have picked a potentially interesting combination of detailed channel and calcium dynamics, with very simplified neuronal and connectivity models. I was not able to find a clear motivation for this particular model configuration. I felt that the entire analysis could have been done with simple alpha-function synapses and integrate-fire neurons. Can the authors justify their model design choices?

- We thank the reviewer for this comment, indeed choosing what level of detail to use is an endemic choice for modelling. There is always a delicate balance between the biophysical detail, that limits model clarity, and simplified models that are amenable to analysis but may not capture the necessary biological data. While some aspects of the model could be done with integrate-and-fire neurons, we feel like important results of the model could be lost with such approach. Namely, the tight control exerted by cholinergic activation of the â▫o7 nAChR, where a detailed description of the release of neurotransmitter and CICR mechanism provides insight into these mechanisms that would be hard to get with integrate-and-fire neurons. We recognize that this justification should be mentioned in the paper, so we added a â€˜Model caveatsâ€{trade mark, serif} part in the discussion where we justify our modeling choices and their limitations (see page 27, lines 622-638).

3. Figure 4 C seems to be the crucial experiment to infer a sharp difference between EPSC responses for different durations of inhibition. This could greatly benefit from more follow up and characterization, as well as a clear statement of number of observations (which might be 5 and 8 respectively, but it isn’t clear). Among other things, it would be good to put in blockers to confirm the affected pathways; and to vary the ACh and electrical stimulus parameters. As it stands this is a single observation which does not provide strong constraints for the modeling.

- The number of observations in figure 4C is 5 slices for each group. We have added â€œn = 5 slices for each groupâ€▫ in the figure legend. This is a direct optogenetic inhibition of Sst or OLM interneurons. There are no appropriate blockers for this manipulation. For the cholinergic pairing study in Figure 2, we have taken the reviewerâ€{trade mark, serif}s suggestion into consideration and considered a shorter cholinergic stimulation period (see Figure 2E and F), which further constraints the model. The parameters Î¸â†”, Î¸â†’ and ð▫›3/4â†’ were readjusted (from 0.28, 0.35 and 0.0783 to 0.31, 0.34 and 0.0675, respectively) to have a better agreement between the shape of the curves 2E and 2F. We note that this produced slight changes in the depression and potentiation time window for single pulses (potentiation time window: [10.4, 131.1] msec instead of [10.3,120.5] msec; depression time window: [-19.9, 10.4] and [131.1, 177.4] msec instead of [-10.6, 10.3] and [120.5, 205] msec ) and doublets (potentiation time window: [10.9, 149.9] msec instead of [10.3,299.3] msec; depression time window: [-19.9, 10.9] and [149.9, 320] msec instead of [-10.7, 10.3] and [299.3, 375] msec ) (lines 372-377).

4. Have the authors performed any parameter sensitivity analyses? Most of their data sources are actually other simulations, not from the primary experimental data. It is difficult to understand whether the results of the study are robust or fragile.

- We agree with the reviewer that further elaborating on this point would be helpful. The revised version of the manuscript includes a subsection that describes which parameter may require more fine tuning as more experimental data is collected and more constraints are imposed on the model (page 16, lines 315-334). We also explain that the model is robust to changes of parameters as long as the ratio of insertion/removal of AMPARs is maintained, and give examples in Extended Data Figure 4-2.

5. The authors do not seem to have provided source code nor a link to a GitHub repository. I am therefore unable to assess the software. The provision of code for modeling studies is essential.

- The code used to simulate our results can be found in : https://github.com/inesCompleto/Hippocampal_Plasticity

Specific points:

6. Line 187: The authors “assume that the alpha-7 nAChR current is not strong enough to elicit action potentials directly” Since they have the cell model, why not calculate this? The introduction of equation 16 seems unnecessary and is fraught with assumptions.

- We have included a plot with the voltage traces of the OLM cells during and before cholinergic pairing (see Extended Data Figure 2-1 and respective caption). The plot shows that the â▫o7 nAChR current is not strong enough to induce spiking. The model can be adapted to consider â▫o7 nAChR induced spiking. However, we feel like advancements in the field, such as a detailed characterization of the impact â▫o7 nAChR stimulation on OLM ongoing activity, are required to justify such model adaptation.

7. Cap of 40 nM (4e-5 mM, line 193) is well below resting calcium, and K(Ca)p of 1nM (line 192) seems extraordinarily steep. Can the authors give a physiological justification for the use of such numbers?

- The parameters Cap and K(Ca)p are such that a pulse of 0.10 uM of amplitude and 1 msec of duration elicits release of GABA and a GABAA synaptic activation function with the same characteristics (amplitude and duration) as described in Destexhe et al., 1998 (see Extended Data Figure 2-2).

8. There is a rather complex internal Ca model in alpha-7 cells, involving CICR. Again, I don’t see why this level of detail contributes to the understanding. If one has to put in complex calcium dynamics it should be for the synaptic plasticity model.

- Extensive work has been done to describe the calcium-induced plasticity mechanisms in great detail (Cummings et al, 1996; Lisman, 1989; Artola and Singer, 1993; Bear and Malenka, 1994; Dudek and Bear, 1991; Luscher and Malenka, 2012). The present work aims at studying the cholinergic mechanisms of LTP in the Schaffer-collateral pathway. Despite being uncommon to consider CICR mechanisms of neurotransmitter release, it is known that the activation of â▫o7 nAChR located on the presynaptic terminal triggers calcium-dependent signalling pathways that enhance glutamate release (Griguoli and Cherubini, 2012; Tsuneki et al, 2000; Dajas-Balador et al, 2002). Since OLM spiking by â▫o7 nAChR activation it has not been clearly characterized experimentally, we believe that the fact the modelling and experimental work agree when considering CICR is of great interest. Moreover, we would like to highlight the accordance of the co-pairing timing of induction of potentiation and depression (as shown in Figure 3 and Extended Data Figures 3-1, 3-2). We expect the detailed description of the CICR dynamics is underlying these good results.

9. Can the authors make a clear distinction between modeling and experimental results? Is Figure 3 modeling or experimental? From the text it seems to be modeling, but the figure legend does not make this clear. The text is also confusing in this regard for Figure 4, though the legend is clearer.

- We have added labels (â€˜Experimentalâ€{trade mark, serif} and â€˜Modelâ€{trade mark, serif}) to help distinguish the experimental and modeling results. We have also clarified the nature of the results of Figure 3 on the respective caption and corresponding main text of Figure 4 (line 391-395, 404-405).

10. Can the authors provide a more detailed schematic of their model? In particular, there is considerable complexity in the dynamics of calcium and of synaptic release, which is invisible in the schematics provided.

- We have altered Figure 8 to include a detailed schematic of the CICR mechanism considered.

11. Line 329: “GABAergic inputs to ED are modeled as a square pulse” If you have such a complex set of cells in the model, why is GABA being treated in this oversimplified way? There is an equation 16 for synaptic release, that one could apply to this synapse, or one could incorporate the GABA term with the GABAR dynamics in the usual alpha-function formulation.

- Our simulations are performed over 40 mints, with a time step in the milliseconds range. This results in very costly computations. By modeling the neurotransmitters as square pulses, we decrease significantly the computation times. Moreover, we show on Extended Data Figure 4-1 (Sup. Fig. 6 in the original manuscript) that a square pulse is a reasonable simplification of the GABA release that results from I-cell spiking. Thus, we feel that changing over to an alpha-function formulation would not change the results of the study.

12. Around line 331 the authors say that other pulse shapes would give the same results, but they say “simulations not shown”. The simulations should be shown.

- We have included simulations of Figure 4 using glutamate and GABA pulses with different characteristics (see Extended Data Figure 4-2).

13. The authors report a sharp change in system properties when disinhibition duration is changed from 5 min to 8 min. Please explain and explore more completely. Is this a phase transition or an analogue effect?

- We detail in the Discussion that the longer the disinhibition period, the higher the increase in gAMPA and, therefore, the longer lasting the changes in synaptic strength (see page 25 of the revised manuscript, lines 568-571). In other words, there is no sharp transition between 5 and 8 minutes from short to long-term plasticity.

14. On line 362 the authors say that gAMPA has only one stable fixed point without synaptic simulation. However, it isn’t stated whether it would become bistable under conditions of regular synaptic stimulation.

- In our simulations and in the experiments, we perform regular synaptic stimulation (the Schaffer-collateral pathway is stimulated once per minute, to which we pair stimulation of the cholinergic pathway during a co-pairing period). In the Results section, we explain that without regular synaptic stimulation, gAMPA decays back to its resting value (page 20, lines 429-431). So there is no bistability observed neither in the model, nor in the experiments.

15. The authors chose to use the Euler method, which is not numerically ideal. How did they ensure accuracy? What timestep did they use? What validation did they carry out to ensure that it worked as expected?

- Although we understand that the Euler method can carry some inaccuracies - in particular when a big timestep is used - this method is widely used to perform computation of noisy ODEâ€{trade mark, serif}s. In our simulations, we used a step size of 0.02 msec. We did the perfunctory checks by using smaller timesteps (for example, 0.01 msec) and observed that it does not produce different results. We provide a detailed justification in the Methods section (page 16, line 331-334).

Reviewer 2

Major comments:

1. Figure 1 shows the nAChR receptors on the presynaptic terminals of OLM cells, but this localisation of receptors is not discussed much in the text. They should mention this in the text and they must provide citations for this anatomical location of nAChR on OLM cells.

- The nAChR can be found on both pre- and postsynaptic synapses of GABAergic interneurons. To our knowledge, â▫o7 nAChR-induced spiking of OLMâ▫o2 interneurons has yet to be reported. Given the lack of experimental evidence to support OLM spiking by nAChR activation, we consider the action of pre-synaptic â▫o7 nAChR. We agree that it would be useful to include a more detailed justification of our choice and further citations, which we have done in the revised manuscript (see page 3, line 53-58; page 11, line 210-218).

2. The direct release of GABA by nicotinic induction of calcium influx is an unusual feature of the model. They need to provide more detail and description about the experimental data supporting this property of the model (not just a citation) and must describe why they do not think the nicotinic receptors are modulating spiking induced release of GABA.

- Even though CICR is not usual considered in modeling work as a mechanism of neurotransmitter release, it is a mechanism that has been broadly described and considered in electrophysiological studies (Griguoli and Cherubini, 2012; Tsuneki et al. 2000; Dajas-Bailador et al, 2002). However, we agree with the reviewer that further elaborating on this point would be helpful. We have revised the methods section where the OLM cell GABA release mechanism is introduced to include more information and relevant citations that support our modeling choices (page 11, line 210-218).

3. They need to be clearer in the methods about the timing and duration of cholinergic stimulation. Currently the description in the methods seems to not match the description in the results. In the methods, they should mention both the 5 min and the 8 min stimulation period and the intervals between stimulation (once per min). (The duration looks shorter than 5 or 8 mins in Figure 2C. Also, the comments about one pulse per minute seem inconsistent with the higher rates (e.g. 1/3) described in Figure legend 3B. Basically, they need to give a clearer overview of the stimulation in the Methods and at the start of the Results section (which could use elements of the description from Figure legend 3B.

- We have revised the methods and results section and hope that the stimulation protocols are now clearer. In particular, we added a â€{trade mark, serif}Stimulation Protocolâ€{trade mark, serif} subsection in the Methods where we detail the stimulation period, frequency, pulse relative timing, and neurotransmitter profiles used in the different section of the manuscript (see pages 15, lines 290-313). We have also labelled Figure 3B, where the period and frequency used in the simulations seem to be confusing to the reader.

4. They need to be much clearer in the figures about what results are from the model simulations and what results are from the experimental work. I suggest adding clear labels of “MODEL” and “DATA” where relevant.

- We followed the reviewerâ€{trade mark, serif}s suggestion and added labels â€˜Experimentalâ€{trade mark, serif} and â€˜Modelâ€{trade mark, serif} to Figure 2 and 4 to distinguished between the model and experimental results. We have also indicated in the figures caption if the results shown were model simulations or experimental data.

5. In the figures, they should separately show the short term acute increase in EPSCs (perhaps with a shorter simulated stimulation protocol) and the long term potentiation of EPSCs. They primarily show the long term potentiation, but don’t seem to show examples of the acute increase without long-term potentiation.

- We have followed the reviewerâ€{trade mark, serif}s advice and included a separate figure with an experimental and simulated shorter co-pairing period (see Figure 2E and F) to verify the acute increase in potentiation.

6. The time difference cut-off points in Figure 3C and D are very abrupt and seem unrealistic, probably due to lack of noise in the calcium concentrations. They should discuss this and be clear about whether they would predict smoother transitions in experimental data if this were tested. They incorporate noise in other simulations, but they don’t seem to be including it in these plots? I liked supplementary figure 7 as this explains how the generation of spiking induces the depression dynamics at low and high time differences. However, if multiple examples similar to this figure were combined with different levels of noise, I would think that noise would change the position of the locus of this temporal transition into spike induction and thereby give less sharp transitions.

- We agree that the transition times between the potentiation and depression regions in Figures 3C and 3D are unrealistically abrupt. As point out by the reviewer, this is indeed due to the lack of noise during these simulations. We repeated the simulations with noisy membrane potential of ED for one-pulse pairing and show how this leads to smoother transitions (see Extended Data Figure 3-2).

7. The authors leave out a lot of relevant citations. They ought to cite earlier work on cholinergic modulation of interneurons, and cholinergic modulation of LTP. For the cholinergic modulation of interneurons, they should cite work by Pitler and Alger, 1992 J. Physiol. 450: 127; Behrends and ten Bruggencate, 1993; J. Neurophysiol. 69:626; Patil, J. Neurophysiol 1999, 81: 2103-2118; Bell et al. 2011 Neuropharmacol 61:1379; Bell ,Bell, McQuiston 2015, J. Physiol. 593: 197.

- The existing literature on cholinergic modulation of interneurons and LTP is incredibly extensive (if not overwhelming). For that reason, we have only considered previous work that directly addresses the induction of plasticity by activation of nAChR in hippocampal interneurons. That being said, we agree that relevant citations are missing. The revised manuscript cites the work by Pitler and Alger, 1992 J. Physiol. 450: 127; Behrends and ten Bruggencate, 1993; J. Neurophysiol. 69:626; Bell et al. 2011 Neuropharmacol 61:1379; Bell ,Bell, McQuiston 2015, J. Physiol. 593: 197; McQuiston and Alkondon, (page 2, lines 36-38 ). We didnâ€{trade mark, serif}t include the work of Patil as it refers to cholinergic modulation of GABAergic interneurons in the piriform cortex and we feel it is out of the scope of this project. Please let us know if there is a specific result from this paper you think it is relevant to our work that we might have missed.

8. The description is ungrammatical in many places. They should have someone with strong English writing skills proofread the paper.

- We have revised the text to address your concerns and hope that it is now clearer. See the specific comments for a detailed description of the changes made.

9. They should be clear about whether the experiments described here were entirely reported in previous papers, or if some component is novel. I believe the experimental data is all previously presented, so this should be stated more clearly in the methods and results sections.

- Figures 2A and C have been previously reported. The remaining experimental results (Figures 2E, 4A and 4C) are novel. The results that have been reported in previous papers include a citation in their caption of the original work.

Specific comments:

Page 2 - There are a number of previous studies that they should cite. Some examples are provided in the major comments. They should also cite work from groups like the McQuiston and Alkondon groups for effects of nicotinic receptors on interneurons.

- As mentioned in the major comments, we have adopted the reviewerâ€{trade mark, serif}s suggestion and included more citations (see Reviewer #2, comment 7).

Page 2 - Should cite Hannah Monyer lab paper in entorhinal cortex showing inhibition induced by cholinergic stimulation. Desikan, Koser, Neitz, Monyer PNAS 115: E2644.

- We appreciate the reviewerâ€{trade mark, serif}s insightful suggestion and agree that there is more work that puts into evidence the role of cholinergic inputs in the inhibition of different types of neurons. However, due to the extensive literature, we restrict our citations to work done on the CA1 neural network that we consider in this study.

Page 2 - Increased SC to CA1 EPSCs - They should provide more details from Gu et al. 2020. They should mention if this was tested by recording of intracellular or field potentials?? They should also specify clearly if this is a short term acute effect or is it a long term potentiation effect. The later mention of “potentiation” suggests it is long term, but they need to be clear about this and should always specify “long term potentiation” or “short term potentiationâ€▫.

- We apologize for the lack of clarity. These are whole cell intracellular recordings (see lines 109-138). We took the reviewers advice and specify in the text if we refer to long or short-term potentiation, and use the term â€˜increaseâ€{trade mark, serif} when we are not referring specifically to short or long term effects (page 3, line 51).

Page 2 - “how this facilitates the potentiation” - This sounds like they are always referring to long term potentiation, but the short term disinhibition could cause acute increases in EPSCs without causing long term potentiation. They need to use clear language.

- We have made the change, and the new sentence reads as follows: â€œâ€¦ how this facilitates the increase of SC-evoked EPSPs of the CA1 pyramidal cells remain elusiveâ€▫ (page 3, line 51).

Page 3 - “reduced inhibitory inputs can directly induce the plasticity” - this is incorrect - they do NOT show that reduced inhibition directly causes plasticity? What they show is that the effect is indirect by allowing increased calcium influx through NMDA channels, thus they should say “inhibitory inputs can indirectly enhance the plasticityâ€▫

- We took the reviewers suggestion (see lines 63-64).

Page 4 - “similar as described in (Gu and Yakel, 2011, 2017) = are they presenting new data? They need to be clear about whether this is new or a review of previous data -

- As mentioned in major comment 9, Figures 2A and C are reported and cited. Figures 2E, 4A and 4C are novel.

Page 4 - Before pairing by student t-test - this is vague - They should say: “testing of statistical significance by students t-testâ€▫?

Page 4- They should itemize what time periods are used for generating the mean and variance in the baseline and experimental effect used in the t-test.

- We have modified the experimental protocol to include the reviewerâ€{trade mark, serif}s suggestion (see page 6, lines 131-136)

Page 6- an action potential in CA1 pyramidal cells is insufficient to make the OLM cell membrane potential cross.... But what about multiple CA1 cells?

- We refer to multiple pyramidal cells, and specify that repeated firing of CA1 pyramidal cells with theta frequency can induce spiking of OLM cells, although we consider a CA1 pyr theta stimulation to be beyond the scope of this paper (lines 151-155).

Line 136 - what simulation package was used? Was it Neuron? It is unlikely that they generated this model from scratch. They need to specify the simulation package or describe how it was programmed from scratch.

- The code used to simulate our results can be found in : https://github.com/inesCompleto/Hippocampal_Plasticity. There you can find the detailed description of the programs used, which were designed from scratch.

Lin 172 - what is the value of tau r alpha 7?? They should specify the table where this is presented later.

- We have included the value of tau r alpha 7 in the text, where it is first introduced (see line 194), as well as in the Table 2.

Lne 183 - as a function of presynaptic voltage - does this mean the shape depends primarily on the action potential? What about all the synaptic release mechanisms and diffusion?

- Similarly to what is done in Destexhe et al, 1998, we consider a simplified neurotransmitter release model. This allows us to considerably decrease the computation time of our model simulations without impact on the results.

Line 189 - calcium concentration sufficient to activate vesicular release (Griguoli and Cherubini, 2012) - they should provide more description of the experimental results supporting this model as this is unusual - is there really enough calcium through nicotinic receptors - are they on the presynaptic terminals of the GABA cells??

- As mentioned in the major comment 2 (Reviewer #2), even though CICR is not usual considered in modeling work as a mechanism of neurotransmitter release, it is a mechanism that has been broadly described and considered in electrophysiological studies. That being said, we took the reviewerâ€{trade mark, serif}s comment into consideration and included more citations and a more detailed justification of our modeling choices (page 11, line 210-218).

Line 210 - tau alpha 7 - They give the source of the value, but when will they actually give the value itself - they should give the table.

- We have included a reference to the Table with the parameter values when they are first mentioned (see line 243).

Lone 222 - same order of magnitude as observed experimentally - they should show this

- We believe that adding a new figure would be unnecessary, as we show the dynamics of calcium in Extended Data Figure 2-1. However, we agree that further description of the results we based our model in should be given, and we added a sentence where we explicit the order of magnitude observed experimentally (line 252).

Lin 252 - tau Ca - should provide the numerical value

- We have included the value of tau Ca in the text (line 280).

Line 267 - previously - should give citation after first sentence

- We have included the reviewerâ€{trade mark, serif}s suggestion in the revised manuscript. The new sentence now reads: â€{trade mark, serif}Similarly to what was reported in Gu et al, 2020â€¦â€{trade mark, serif} (line 345).

Line 217 - OLMalpha2 interneuronal ...- interneuronal what? A word seems to be missing.

- Due to modifications made to address other comments, in particular the major comment 1 (reviewer #1), this sentence no longer appears in the revised manuscript.

Line 268 - “8 times” and line “8 minutes” - the description in the methods seems incomplete or inconsistent relative to the discussion in the Results - 8 times is not equivalent to 8 minutes and neither matches 10 pulses as described in the methods. As noted in major comments, the methods should describe the details as presented in the figure.

- In this particular case, 8 times is equivalent to 8 minutes since stimulation of the pathway is done once per minute. However, we have taken the reviewerâ€{trade mark, serif}s comment into account and, as described in major comment 3 we have included a new subsection in the Methods that describes the different simulation protocols used in the different studies (see pages 15, lines 290-313).

Line 270 - The induction of potentiation - is this long term or short term potentiation - the term potentiation is ambiguous and could mean short term acute increase or a longer term sustained increase

- As mentioned in a previous specific comment, the new revised manuscript as suffer structural modifications where this sentence is not included.

Line 280 - on the postsynaptic nAChR - again are these on the presynaptic terminals of the OLM cells - otherwise it’s hard to see how they could cause calcium influx that would cause direct release of GABA

- This is a typo. We thank the reviewer for pointing this out. We have revised the manuscript to now mention presynaptic nAChR (line 296).

Line 285 - duration of 5 msec - this seems inconsistent with the amperometry studies of Parikh et al. 2007, Neuron 56: 141-154. showing durations of ACh increases around 2 seconds long.

They should discuss this explicitly as their duration seems too short.

- In Parikh et al. 2007, there is a different pattern of activation of the cholinergic neurons, which can then result in different duration of the neurotransmitter. Moreover, this study is performed in the mPFC and motor cortex - although it can be used as a reference, it is not clear that ACh as the same profile in the synapses of the hippocampal network. That being said, we clarify in the Methods how changes in the neurotransmitter concentration change our results, and how the model can be modified as more data concerning the profile of ACh in the synaptic cleft is collected (lines 323-330).

Line 291 - results in ED inhibition - should be clearer by saying inhibition of the pyramidal cell dendritic compartment -

- We have made the change and the new sentence reads as follows: ‘This results in the inhibition of the pyramidal cell dendritic compartment ED â€˜ (lines 351-352).

Line 302 - co-paring

- We have correct the spelling (line 363).

Line 312 and 316 - They need to be clear about the direction of the subtraction yielding delta t- otherwise the results about 100 msec versus 10 msec can be confusing - make clear that 10msec=

- Figure 3 includes a schematic representation of the neurotransmitter arrival time, where it is explicitly represented the direction of -âˆ†t and âˆ†t.

Line 329 - sup figure 6 - why is this the first one shown? Ordering of supplementary figures seems odd

- We have revised the order of the figures to follow the correct order. We note that due to the journalâ€{trade mark, serif}s submission policies the supplementary figures are referenced as Extended Data in the revised version.

Line 370 - smaller amplitude - is this due to changes in release from internal stores or changes in NMDA channel conductance?

- In this section, we study the disinhibitory mechanisms of plasticity induction. As mentioned in the subsection introductory paragraph (line 390-394), and in the caption of Figure 4, we are analyzing the dendritic compartmentâ€{trade mark, serif}s response to pulse of Glutamate and GABA. Therefore, we are not referring to changes from internal stores since the activity of OLM cells is not being explicitly analyzed.

Line 399 - ’Given that this..., indicates...- this is not grammatical

- We thank the reviewerâ€{trade mark, serif}s for pointing this out - we have corrected the sentence (line 471-472).

Page 18 - They should insert paragraphs breaks somewhere on this page

- We have taken the reviewerâ€{trade mark, serif}s advice into account and added paragraph breaks in this page (page 19-20 of revised manuscript).

Line 428 - ’during cholinergic pairing = not only would EPSCs decrease, but they seem to predict a direct IPSC in fast spiking interneurons caused by nicotinic activation after cholinergic simulation

- Yes, this is a prediction of our model. We thank the reviewer for pointing this out.

Line 448 - “first report” - should avoid trying to claim primacy. There are lots of earlier studies of LTP showing that blockade of inhibition enhances LTP.

- We agree that there are extensive studies that show that blockade of inhibition facilitates LTP. This is however, the first experimental and modeling work to show how the duration of the disinhibition period modulates this form of plasticity induction. As it was written in the original manuscript, this was not clear. We then changed the sentence to ‘To our knowledge, this is the first report to reveal how repeated disinhibition can directly induce short or long-term potentiation, depending on the duration of the disinhibition period (both experimentally and computationally)â€{trade mark, serif} (lines 523-525).

Line 450 - “first computational study” - Other studies have analyzed effects of cholinergic modulation though not these specific timing effects of nicotinic receptors (e.g. Hasselmo and Barkai, 1995; Barkai et al. 1994, J. Neurophysiol.).

- The full sentence reads ‘the first computational study that explicitly shows how cholinergic action on OLM interneurons can directly induce SC-CA1 plasticity through disinhibitionâ€{trade mark, serif}. To our knowledge, this is the first computational study that shows how activation of the septal cholinergic pathway disinhibits the CA1 pyramidal cells.

Line 454 - expressing SST - the OLM cells also express SST in addition to the bistratified cells - so it is not clear why they can separately manipulate SST cells

- OLMâ▫o2 interneurons constitute a subset of SST neurons (about 30%) and are selectively identified with a2-nAChR expression. We can target OLMâ▫o2 cells by using the interneurons specific marker (LeÃ£o et al, 2012; Mikulovic et al 2015).

Line 460 - “activating cholinergic inputs...synaptic plasticity” - they should cite the many earlier studies showing cholinergic enhancement of LTP in hippocampus and other structures - For effects on LTP, they should cite Burgard and Sarvey 1990 Neurosci. Lett 116: 34; Hasselmo, Barkai 1995, J. Neurophysiol. 15: 6592; Patil, J. Neurophysiol. 1998, 80:2467 showed timing dependent effects.

- The studies here mentioned analyze results obtained in other brain regions (namely the dentate gyrus and piriform cortex). As mentioned in the major comment 7, the existing literature on cholinergic enhancement of LTP is extensive. For that reason, we focus solely on the studies performed in the CA1 hippocampal region. We did however, included the work of MacKay, Placzek and Dani, 2007 (line 538).

Line 499 - “SST interneurons...that were not OLM” - how was this determined? This is confusing.

- As mentioned above, specific marker for OLM interneurons have been previously identified by LeÃ£o et al. (2012) and Mikulovic et al. (2015).

Line 526 - inducing theta oscillations - the influence of septum on theta oscillations has also been shown by Toth, Freund and Miles J. Physiol. 1997 500: 463 and by the Sylvain Williams group (Sotty et al. 2003 J Physiol 551:927.)- these should be cited.

It’s good that they cite the work on the role of OLM cells in theta oscillations (Mikulovic et al., 2018, 9: 3638), but there is other relevant work. They should also cite here the work by Rotstein et al. 2005 on the role of OLM cells in theta rhythm here which they cite in the methods section but should be cited here.

- We have taken the reviewerâ€{trade mark, serif}s comment into account and added more citations (line 606-608).

Line 535 - “change theta generation’s mechanisms” - this is an awkward phrase. It is also very implausible as the long term potentiation is slow, whereas the induction of theta can be very rapid, so it is likely that it is due to modulation of the GABAergic input coupled with direct cholinergic depolarization of neurons. The text should be altered to reduce the emphasis on long-term potentiation and mention the mechanisms of rapid modulation of theta (e.g. Buzsaki, 2002; Goutagny, Jackson, Williams , 2009 Nat. Neuro 12: 1491.

- We have fixed the error and the sentence now reads â€˜â€¦ and change the mechanisms of theta generationâ€{trade mark, serif} (line 613). Here, we are not referring to the mechanisms of rapid modulation of theta, as it has been done in previous studies, but in the slow modulation of the mechanisms of theta generation. Moreover, we imply that the form of induction of LTP here studied can be at the origin of the observations made by Gu and Yakel, 2017 (lines 599-606).

Line 540 - “cholinergic dysfunction action” - this is ungrammatical

- We have fixed the error (line 618)

Figures 2 and 3 - They need to be very clear about what results in these figures are simulations from the model and what sections are showing experimental data (adding labels of “data” and “model” would help).

- We have added labels to Figures 2 and 4, and mention in the caption of Figure 3 the nature of the results.

Page 36 - “sst and OLMalpha2” - I always understood OLM cells to be SST positive. How are they able to differentiate OLM versus SST cells? This is very confusing.

- We can use the OLMâ▫o2 specific marker to distinguish it from the other SSt interneurons (see cited LeÃ£o et al, 2012).

Page 39 - “by adding a second source” -I don’t recall this being mentioned in the methods and results - this should be stated more clearly in the results at least.

- This is mentioned in the Results section (line 462-463)

Page 43 - Do they have data on inhibition in interneurons induced by ACh activation? They should definitely cite the paper from the Monyer lab (Desikan et al. citation provided above) in which activation of cholinergic terminals was shown to cause brief inhibition in pyramidal cells (and discuss why they did not include this in their model and instead put the inhibition into fast spiking interneurons).

- As mentioned in LeÃ£o et al, 2012, cholinergic activation of OLM causes inhibition of the distal dendrites of pyramidal cells, while causing disinhibition of the proximal dendrites. Since the SC synapse at the proximal dendrites, and not on the distal, we only consider the effect of cholinergic activation on the proximal dendrites of the CA1 pyramidal cell. This is discussed in the introduction (line 45-49), in the methods (line 143-155), and in the discussion (line 528-533).

Supplemental figure 4 - “two spikes” - they should note that this is shown with the dashed line and then note what the solid line represents.

- We have implemented this suggestion (see caption of Extended Data Figure 2-1 â€” Supplementary Figure 4 in the original manuscript).

Page 49 - “membrane potential of the I cell” - this is a very non-physiological time course for membrane potential. Usually stimulation of axonal input causes a dual-exponential time course, not a square wave (even if the presynaptic release is modeled as a square wave).

- Even though a square pulse for the GABA release from the I-cell is non-physiological, we show that this simplified description of the neurotransmitter release elicits a synaptic activation function with the same shape as a detailed model of neurotransmitter release (see Extended Data Figure 2-2).

We would like to thank the referees again for taking the time to review our manuscript.

Best wishes,

InÃªs Guerreiro (in behalf of the authors)
