# Supplementary figures and images for: Recurring Cholinergic Inputs Induce Local Hippocampal Plasticity through Feedforward Disinhibition
Source: eNeuro. 2022 Sep 1;9(5):ENEURO.0389-21.2022. doi: 10.1523/ENEURO.0389-21.2022 (PMC9463983; doi:10.1523/ENEURO.0389-21.2022)

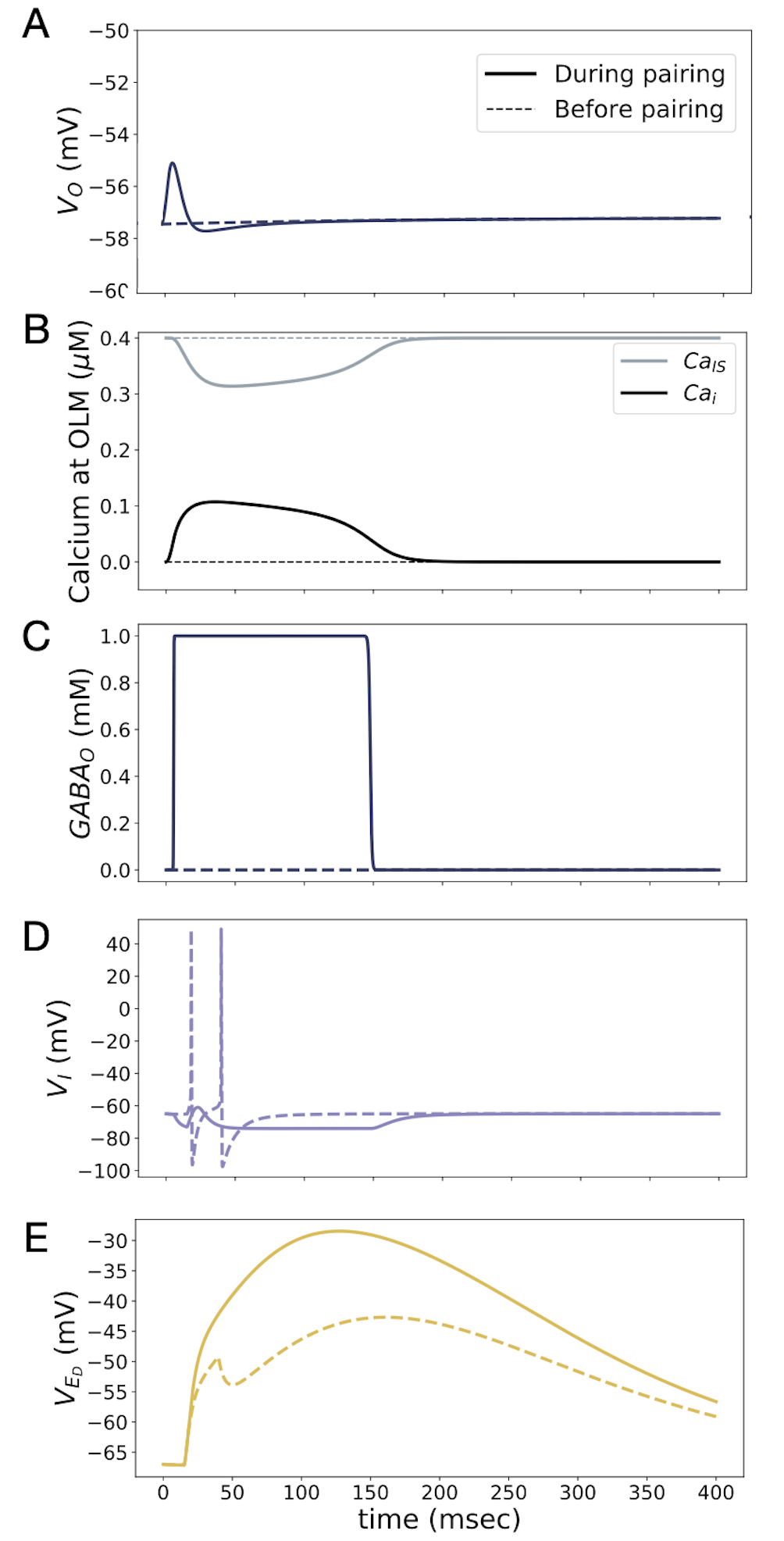

Supplement: Figure 2-1 — A, Before copairing, the α7 nAChR at OLM is not activated, and the OLM cell is not depolarized (dashed line). During copairing, OLM receives a square pulse of ACh with an amplitude of 1 mm and 5 ms of duration (solid line). The OLM is weakly depolarized (solid line). B, Before copairing, there are no changes in the intracellular calcium concentration Cai (dashed line). During copairing, calcium through α7 nAChR triggers CICR mechanisms that increase the intracellular calcium concentration of the O-cell (solid line). C, An increase in intracellular calcium results in GABA release from the O-cell (GABAO). The neurotransmitter concentration is calculated according to the simplified model (solid line). D, The release of GABAO during copairing suppresses spiking of the I-cell evoked by glutamatergic activation (solid line). E, Before copairing, the spiking of the I-cell is not suppressed and inhibits ED, which cannot depolarize a lot (dashed line). During copairing, ED does not receive inhibition, only excitation from glutamatergic stimulation, and it depolarizes (solid line). Download Figure 2-1, TIF file. [file enu-eN-NWR-0389-21-s02.tif]

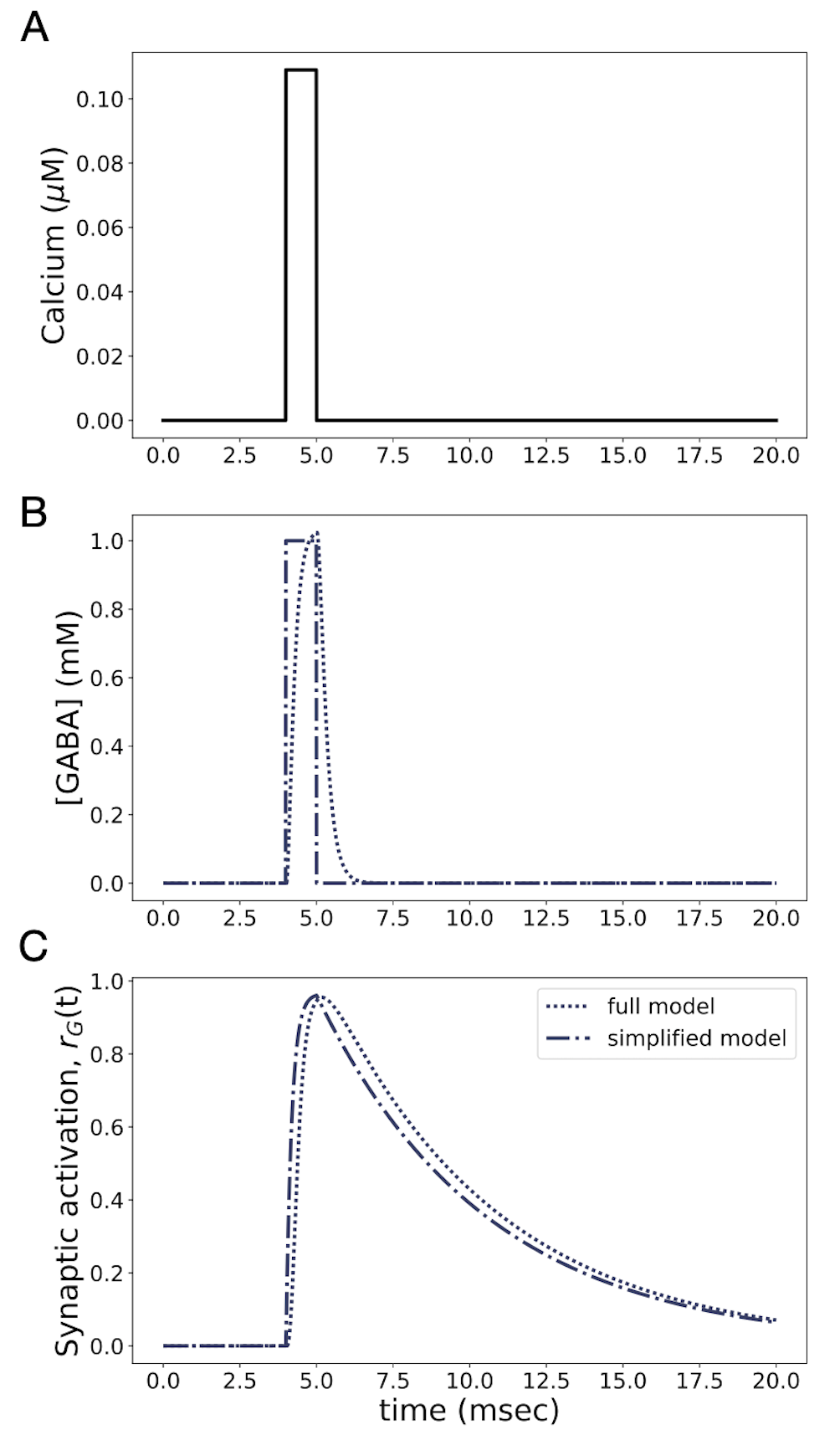

Supplement: Figure 2-2 — Simplified neurotransmitter release model. A, Square calcium pulse of 0.10 μm amplitude and 1 ms of duration. B, GABA concentration elicited by a calcium pulse of 0.10 μm amplitude and 1 ms of duration computed using the detailed model of transmitter release described in the study by Destexhe et al. (1998) and using Equation 16. C, Both models of GABA concentration elicit similar synaptic activation functions, rG (described by Eq. 14 with αG = 5 ms/m and βG = 0.18 ms). Download Figure 2-2, TIF file. [file enu-eN-NWR-0389-21-s03.tif]

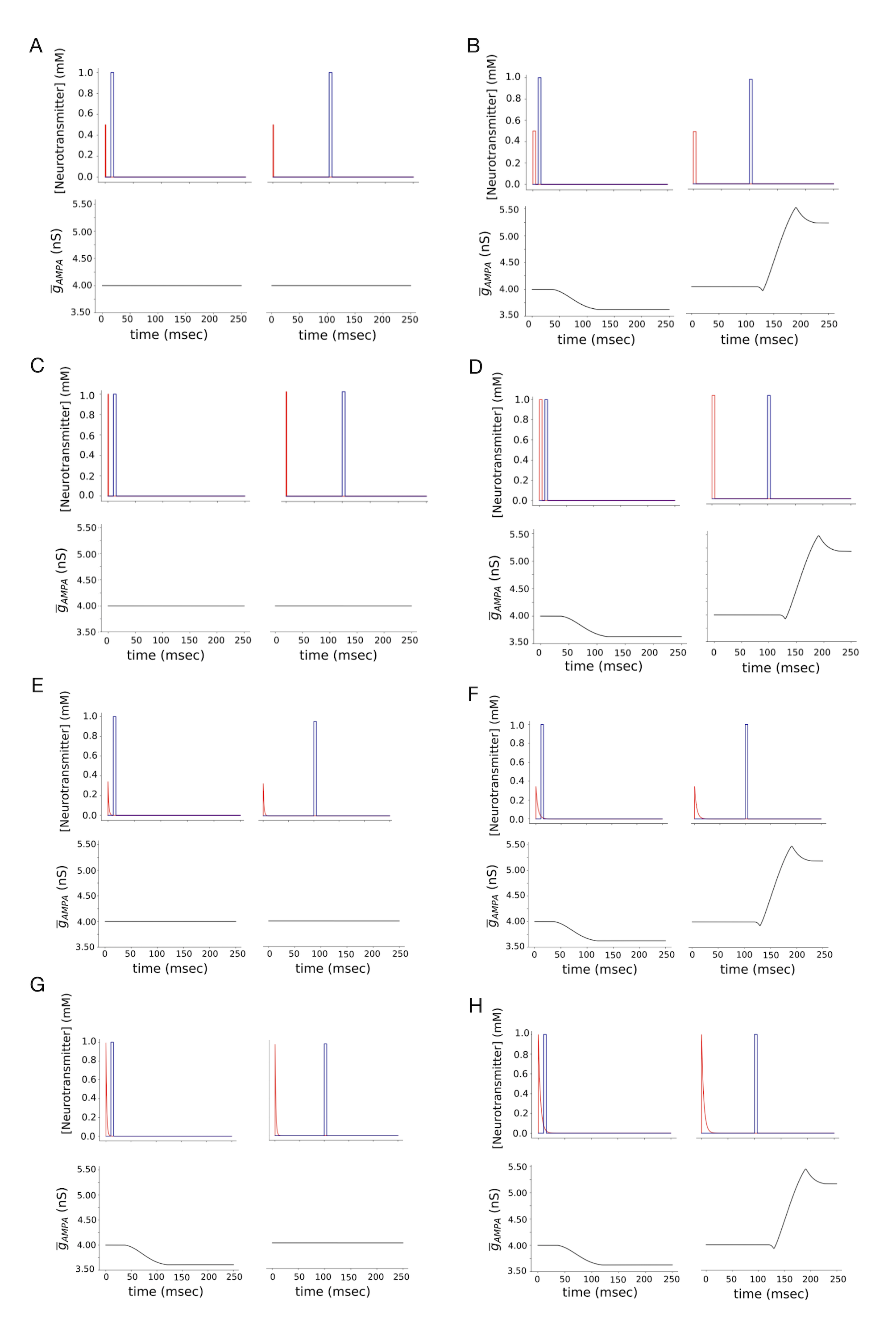

Supplement: Figure 2-3 — Not much is known about the ACh profile in the synaptic cleft upon release from cholinergic neurons; more specifically, not much is known about the time it takes for ACh to be broken down by the cholinesterase and therefore, how long it is available to bind to the cholinergic receptors. We consider the observations made by Gu and Yakel (2011) that pairing cholinergic inputs 10 ms prior to SC stimulation induces depression of the SC–CA1 synapse, while if the cholinergic inputs are activated 100 ms prior to SC stimulation, potentiation is induced. A–D, A square pulse of ACh followed by a pulse of glutamate 10 and 100 ms after will induce, respectively, depression or potentiation if the duration of the ACh pulse is equal or greater than the glutamate. E–H, If ACh is described by an α function with an instantaneous rise time; the smaller the amplitude of the ACh pulse, the longer the decay time needs to be for the results to agree with those in the study by Gu and Yakel (2011). That being said, we model ACh as a square pulse with a duration of 5 ms and concentration of 1 mm, similar to glutamate. Please note that the decay and duration times, as well as the amplitude, of both the ACh and glutamate pulses serve merely as a guide to what types of neurotransmitter profiles we should consider. They are qualitative, and not quantitative, predictions of the synaptic profile of ACh. Copairing of one pulse of ACh (with different synaptic profiles) with one square pulse of glutamate (with a duration of 5 ms and amplitude of 1 mm) for a relative pairing time Δt of 10 and 100 ms. A, Left, One square pulse of ACh with a duration of 1 ms and concentration of 0.5 mm followed 10 ms after by a square pulse of glutamate produces no changes in the maximal conductance of AMPAR, g¯AMPA. Right, Similarly, If the pulse of glutamate arrives 100 ms after, no changes are induced. B, Left, One square pulse of ACh with a duration of 5 ms and a concentration of 0.5 mm followed 10 ms after by a pul [file enu-eN-NWR-0389-21-s04.tif]

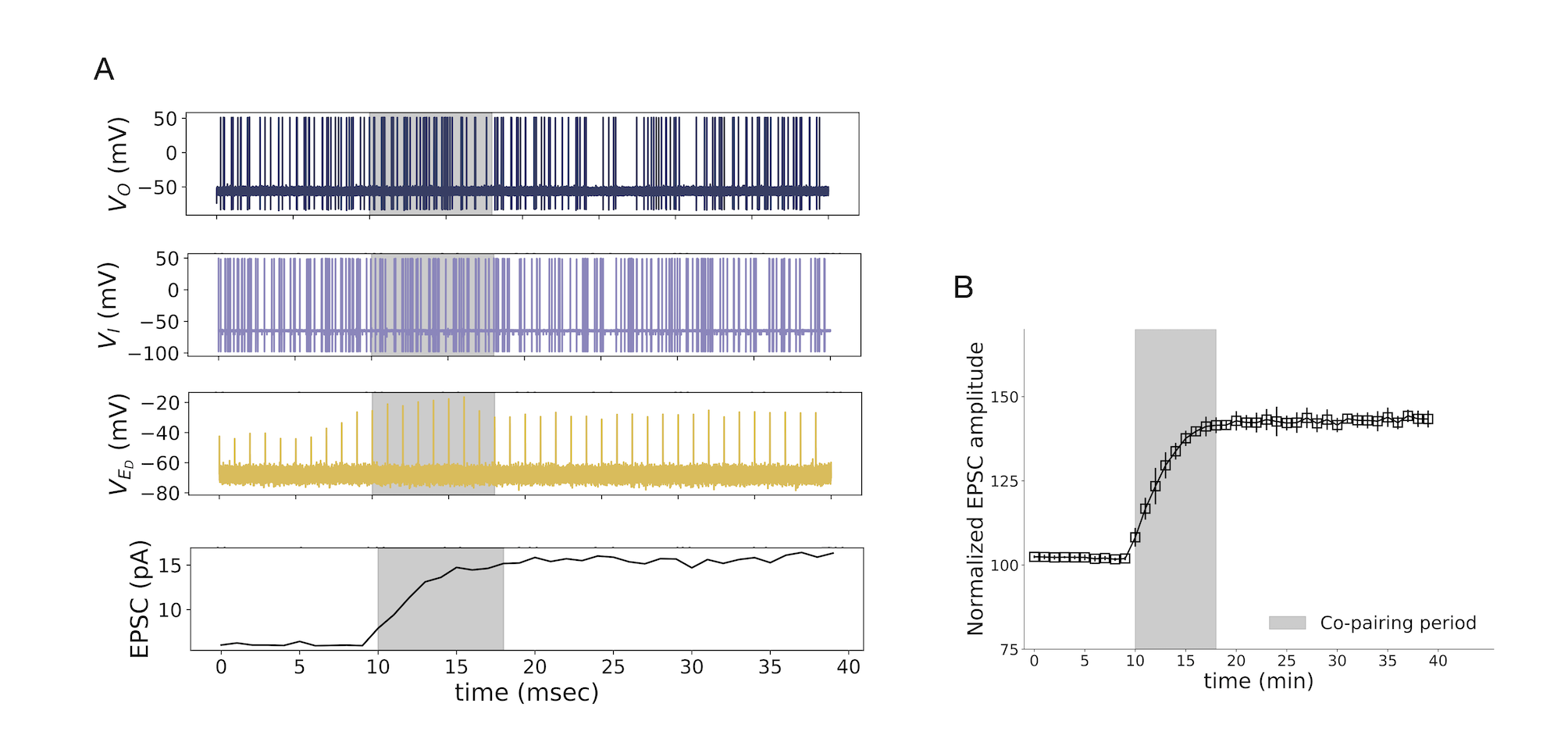

Supplement: Figure 2-4 — A, Time evolution of the membrane potential of the O-cell, I-cell, and ED with noisy background currents when cholinergic inputs are paired with SC inputs, and resultant EPSCs. B, Mean trace of normalized EPSCs after 10 simulations. Adding a noisy background current to the O-cell and I-cell induces spontaneous spiking. Copairing cholinergic and glutamatergic inputs from t = 10 min to t = 18 min induces potentiation of the pyramidal cell EPSC. The O-cell releases GABA when the intracellular calcium concentration is high enough (Eq. 16) and when the cell spikes (Eq. 15). All the remaining parameters are identical to the ones used to produce Figure 6. Noise was incorporated by adding a stochastic term dtζ, where ζ is a random Gaussian variable with a mean of μ = 0 and an SD of σ (=1.1, 0.1, and 0.2 for the O-cells, I-cells, and ED, respectively), to the Euler equations describing the Vx. Normalization of the results was calculated according with the expression (100 + (EPSC – EPSCmin) · (150 – 100))/(EPSCmax – EPSCmin). Download Figure 2-4, TIF file. [file enu-eN-NWR-0389-21-s01.tif]

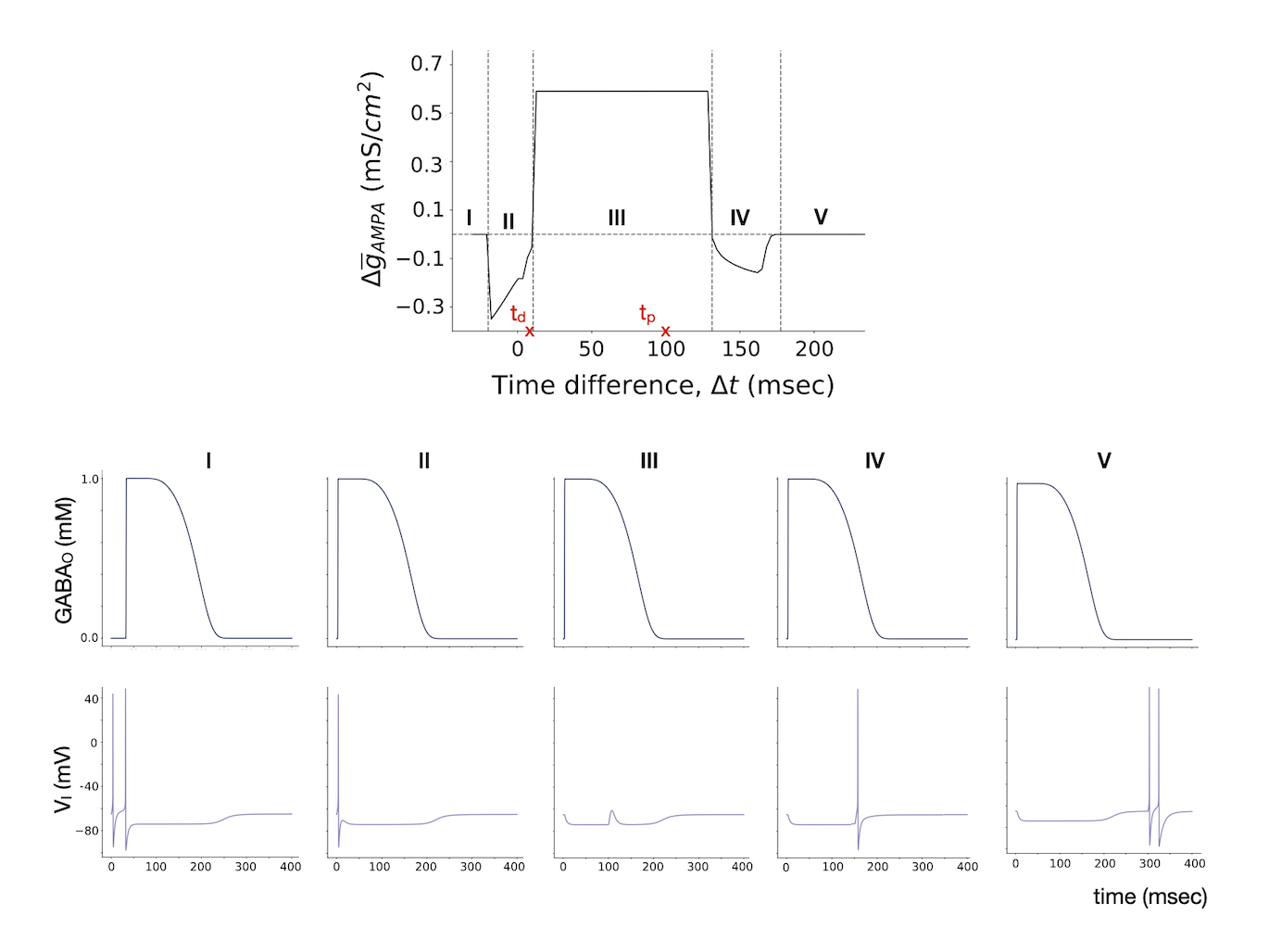

Supplement: Figure 3-1 — Tightly timed pairing of cholinergic to glutamatergic inputs can cancel the I-cell feedforward inhibition. For Δt = –30 ms (Region I), a pulse of glutamate activates the I-cell. When the OLM cell receives a pulse of ACh 30 ms after and releases GABA, the I-cell already emitted two spikes and inhibit ED, no plasticity is induced. For Δt =0 ms (Region II), the I-cell and OLM receive a pulse of glutamate and ACh, respectively, simultaneously. Due to its fast dynamics, the I-cell manages to emit one spike before being inhibited by GABAO. The I-cell inhibits ED only moderately and depression is induced. For Δt = 100 ms (Region III), OLM receives an ACh pulse at t = 0 ms and releases GABAO into the I-cell. When the I-cell receives glutamate 100 ms after, it is hyperpolarized and cannot spike; potentiation is induced. For Δt = 150 ms (Region IV), the hyperpolarization of the I-cell is starting to wear off and the cell manages to emit one spike, sending moderate inhibition to ED; depression is induced. For Δt = 300 ms (Region V), the I-cell can emit two spikes when it receives glutamate 300 ms after cholinergic activation; no plasticity is induced. Download Figure 3-1, TIF file. [file enu-eN-NWR-0389-21-s05.tif]

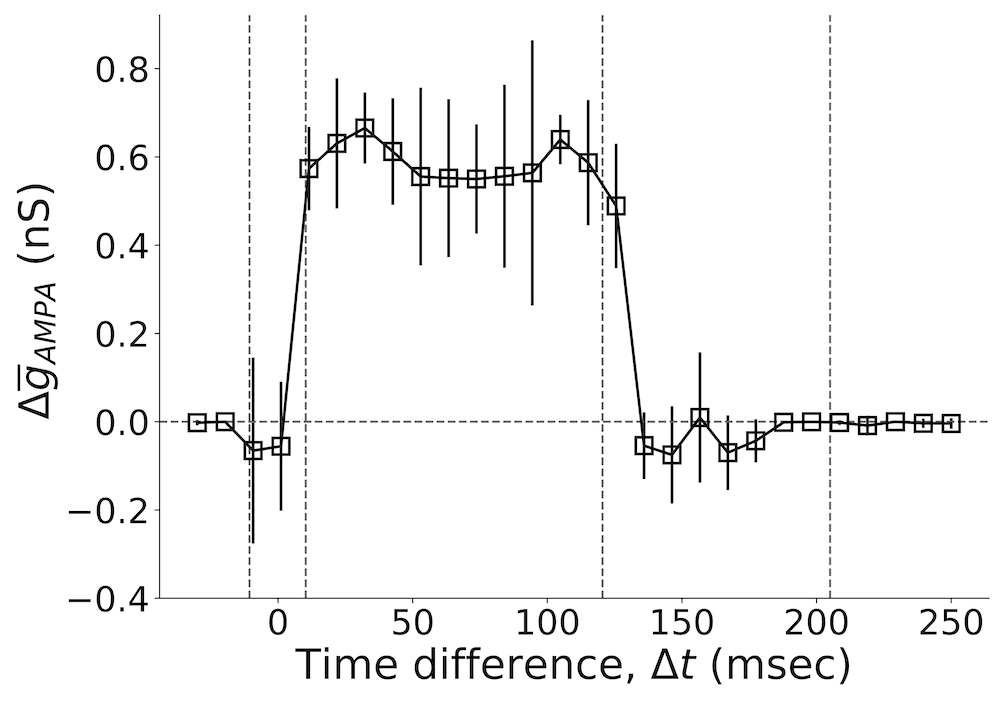

Supplement: Figure 3-2 — Mean relative pairing timing of single pulses of ACh and glutamate with noisy membrane potential of ED after 10 simulations. Noise was incorporated by adding a stochastic term dtζ, where ζ a random Gaussian variable with a mean of μ = 0 and an SD of σ = 0 to the Euler equations describing the VED. The mean trace of normalized EPSCs after 10 simulations. When a noisy membrane potential is considered, the transition between the depression and potentiation windows is less sharp (Fig. 3C, comparison). Download Figure 3-2, TIF file. [file enu-eN-NWR-0389-21-s06.tif]

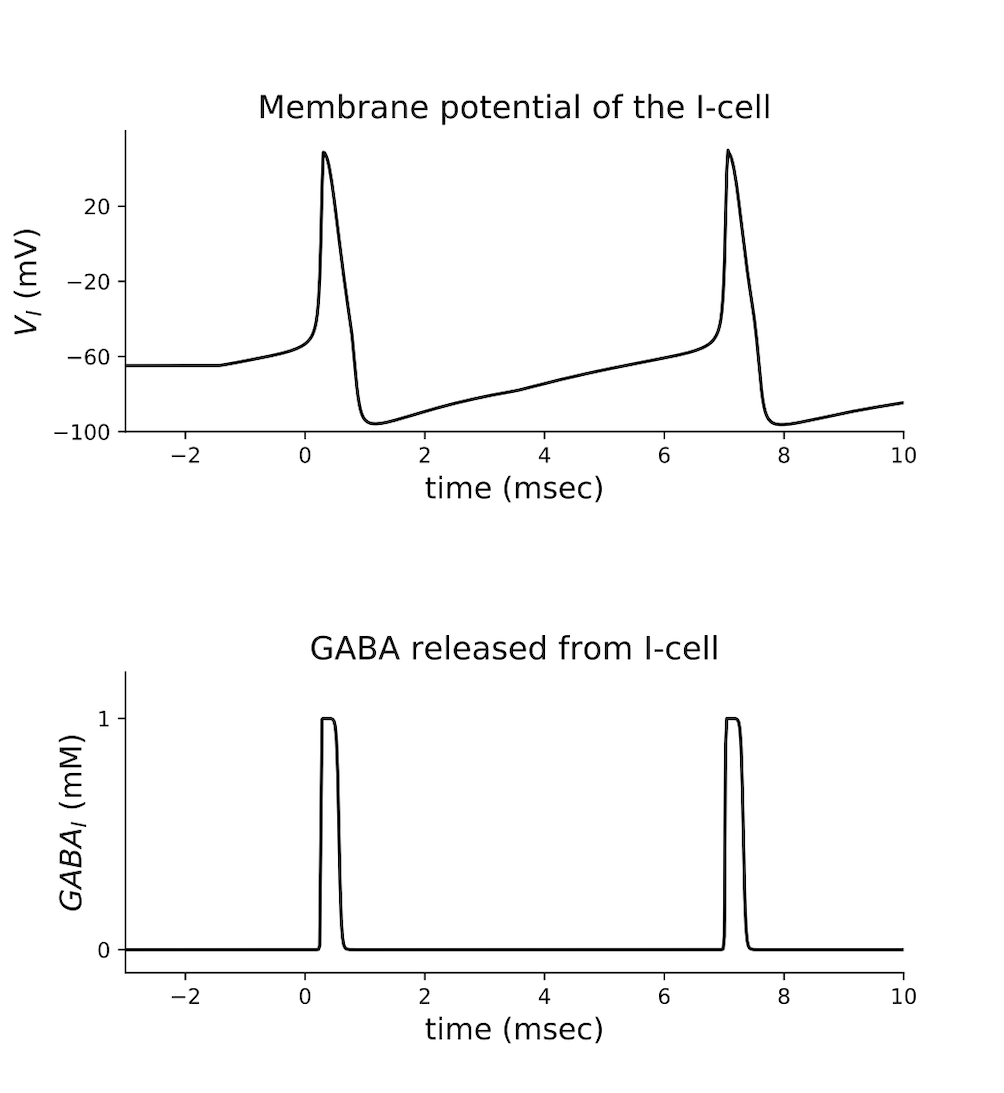

Supplement: Figure 4-1 — I-cell GABA release evoked can be approximated by a square function. A, Membrane potential of the I-cell when it receives two pulses of glutamate (with an amplitude of 1 mm and a duration of 3 ms) with a frequency of 0.2 ms. B, GABA release from I-cell when it receives the action potentials described in A, calculated using Equation 15. Download Figure 4-1, TIF file. [file enu-eN-NWR-0389-21-s07.tif]

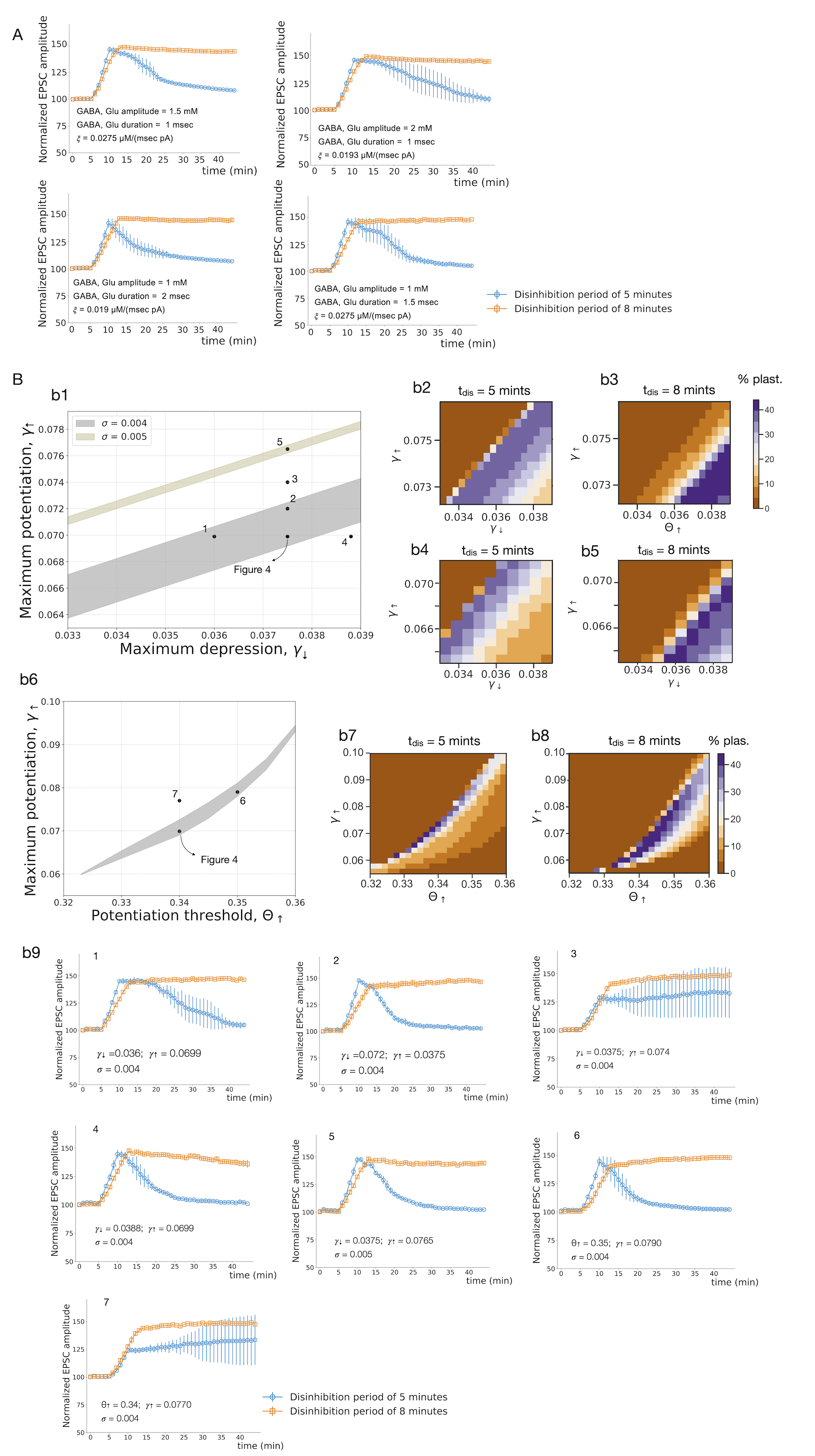

Supplement: Figure 4-2 — Sets of parameters that qualitatively reproduce Figure 4D. A, Numerical simulations of normalized EPSCs of ED for varying the amplitude and duration of the glutamate and GABA pulses. B, Parameters of maximum depression (γ↓), maximum potentiation (γ↑), synaptic plasticity decay constant (σ), and potentiation threshold (θ↑) from the shaded areas qualitatively reproduce Figure 4D. The quality of EPSC traces generated with different parameters was evaluated by measuring the relative variations of EPSC amplitude (in non-normalized and non-noisy simulations) from 5 to 30 min after the disinhibition period was over for a 5 and 8 min disinhibition period. Simulations were the variation (percentage of plasticity) was<4% and >22% for the long and short disinhibition periods, respectively, and were considered to conserve the shape of the experimental EPSC trace. This ensures that, for the long disinhibition period, the EPSCs do not decay faster than the experimental EPSCs observed, or slower, for the case of the short period, and therefore have a similar shape. Experimental measures describe the relative increase in EPSC amplitude from the baseline value to 5 min (%(5-B)) and 30 min (%(30-B)) after the disinhibition period is over (see the Results section for the values of %(5-B) and %(30-B) for 5 and 8 min disinhibition periods). This allows us to derive the relative changes from 5 to 30 min [%(30-5) = (%(30-B) – %(5-B))/(100 + %(5-B)) × 100]. By considering the relative changes between 5 and 30 min after the disinhibition period instead of the changes between the baseline and 5 and 30 min, we decrease the number of conditions to evaluate and the computational cost of performing the parameter exploration. The gray and beige areas represent the parameter space where both conditions are met. Note that increasing the synaptic plasticity decay constant σ decreases the robustness of the model to variations of the maximum depression and potentiation, γ↓ and γ↑ (B, beige area). On t [file enu-eN-NWR-0389-21-s10.tif]

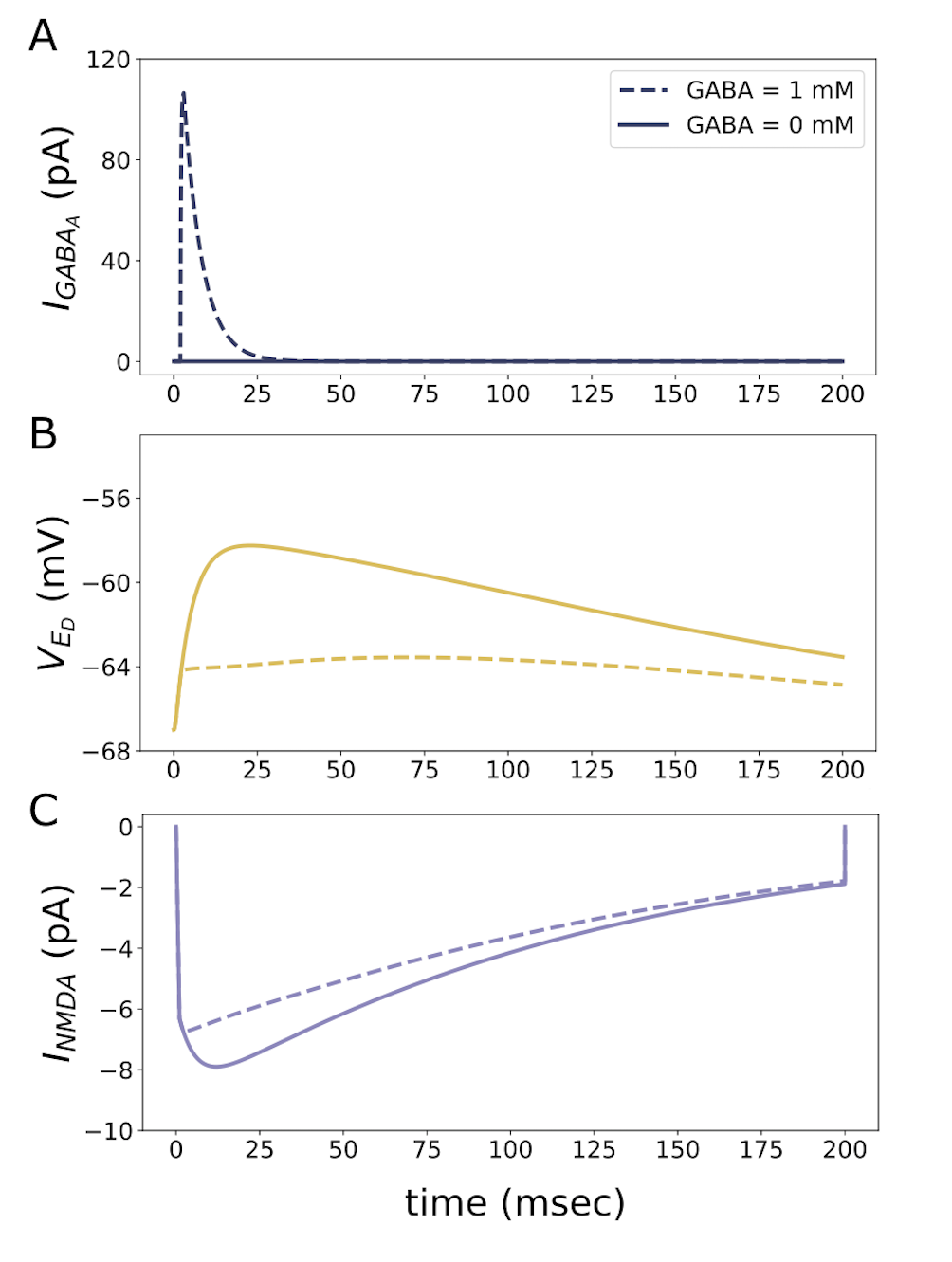

Supplement: Figure 4-3 — A square GABA pulse with 1 mm amplitude and 1 ms of duration evokes a GABAA current at ED, and decrease NMDA current and depolarization. A, One square pulse of GABA with 1 mm amplitude and 1 ms of duration evokes an inhibitory GABAA current at ED (IGABAA). B, When ED receives a GABA square pulse, glutamatergic activation of ED only evokes a depolarization of –63.56 mV (dashed line). C, When ED does not receive GABA inputs, glutamate inputs evoke a depolarization of –58.25 mV (solid line). When ED does not receive GABA inputs, glutamatergic activation evokes a NMDA current of 7.90 pA (solid line). When it receives a GABA square pulse, the evoked NMDA current is 6.75 pA (dashed line). Download Figure 4-3, TIF file. [file enu-eN-NWR-0389-21-s08.tif]

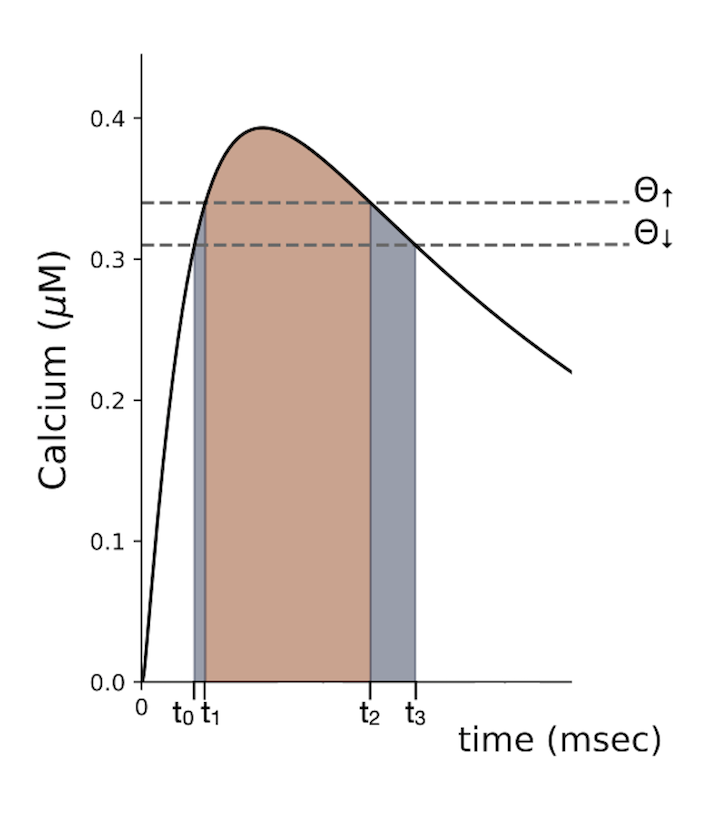

Supplement: Figure 6-1 — Area of potentiation (orange) and area of depression (gray) considered to calculate the (A↑/A↓)w. For the description of the labels, please refer to Figure 6 in the main text. From t0 to t1 and t2 to t3, calcium is above θ↓ and below θ↑. These regions constitute the area of depression A↓. From t1 to t2, calcium is above θ↑. This region constitutes the area of potentiation A↑. While the calcium concentration is above the depression onset θ↓ (but below the potentiation onset θ↑), the maximal conductance of the AMPARs g¯AMPA is decreasing. On the other hand, when the calcium concentration is above θ↑,< g¯AMPA is increasing. The induction of plasticity at the excitatory synapse depends on the net result of these changes of g¯AMPA. The more time calcium spends above θ↑/θ↓, the more likely it is that potentiation/depression is induced at the synapse. Furthermore, the more time calcium spends above θ↑/θ↓, the bigger the area underneath the calcium curve in this region of insertion/removal of AMPARs. Therefore, the ratio between the area of insertion and the area of removal (A↑/A↓) can be used as a measure of induction of plasticity (Fig. 6, main text). There is an optimal ratio for which the decrease of g¯AMPA resultant from time spent in the removal region and the increase of g¯AMPA resultant from time spent in the insertion region will cancel each other and no plasticity is induced. If the ratio A↑/A↓ is below this value, depression is induced; if the ratio is above this value, potentiation is induced. The ratio A↑/A↓ is given by ∫t1t2Ca dt∫t0t1Ca dt+ ∫t2t3Ca dt. Because the decrease and increase of g¯AMPA is not the same in the whole removal and insertion region, we need to calculate the calcium integral weighted by the calcium-dependent learning rate η. The (A↑/A↓)w is then given by ∫t1t2Ca.η dt∫t0t1Ca.η dt+ ∫t2t3Ca.η dt . To calculate (A↑/A↓)w, we use the trapezoidal rule to perform numerical integration of the potentiation and depression area. Download Figure 6-1, TI [file enu-eN-NWR-0389-21-s09.tif]
